# Human liver-derived organoids recapitulate Oropouche virus infection and manifestation, enabling antiviral drug discovery

**DOI:** 10.1016/j.xcrm.2026.102646

**Published:** 2026-03-09

**Authors:** Jiajing Li, Xin Wang, Yibo Ding, Fang Qin, Shirlene T.S. de Lima, Lito Papamichail, Rick Schraauwen, Julia Forato, Ingra M. Claro, Xinyi Hua, Leda M. Simões Mello, Dewy Mae Offermans, Monique M.A. Verstegen, Marjan Boter, Maikel P. Peppelenbosch, Anna Barbiero, Elisabetta Pagani, Harry L.A. Janssen, José A. Telmos Silva, Magnun N.N. dos Santos, Eder C. Pincinato, José Luiz Proenca-Modena, Pengfei Li, Adam A. Anas, Luc J.W. van der Laan, Concetta Castilletti, Bas B. Oude Munnink, William M. de Souza, Wenshi Wang, Qiuwei Pan

**Affiliations:** 1 Department of Gastroenterology and Hepatology, Erasmus MC-University Medical Center, Rotterdam, the Netherlands; 2 Department of Pathogen Biology and Immunology, Jiangsu Key Laboratory of Immunity and Metabolism, Jiangsu International Laboratory of Immunity and Metabolism, Xuzhou Medical University, Xuzhou 221004, China; 3 Department of Microbiology, Immunology, and Molecular Genetics, College of Medicine, University of Kentucky, Lexington, KY, USA; 4 Laboratório Central de Saúde Pública do Ceará, Fortaleza, Brazil; 5 Laboratory of Emerging Viruses, Department of Genetics, Microbiology and Immunology, Institute of Biology, University of Campinas, Campinas, Brazil; 6 Department of Surgery, Erasmus MC Transplant Institute, University Medical Center Rotterdam, Rotterdam, the Netherlands; 7 Department of Internal Medicine, Erasmus MC Transplant Institute, University Medical Center Rotterdam, Rotterdam, the Netherlands; 8 Department of Pathology, Erasmus MC-University Medical Center, Rotterdam, the Netherlands; 9 Department of Viroscience, Erasmus MC-University Medical Center, Rotterdam, the Netherlands; 10 Experimental and Clinical Medicine Department, University of Florence, Florence, Italy; 11 Laboratory of Microbiology and Virology, Provincial Hospital of Bolzano (SABES-ASDAA), Lehrkrankenhaus der Paracelsus Medizinischen Privatuniversität, Bolzano, Italy; 12 Toronto Centre for Liver Disease, Toronto General Hospital, University Health Network, Toronto, ON, Canada; 13 Laboratório do Hospital da Mulher de Fortaleza, Fortaleza, Brazil; 14 Department of Clinical Pathology, School of Medical Sciences, University of Campinas, Campinas, Brazil; 15 Department of Medical Microbiology and Infectious Diseases, Erasmus MC-University Medical Center, Rotterdam, the Netherlands; 16 Department of Internal Medicine, Section of Infectious Diseases, Erasmus MC-University Medical Center, Rotterdam, the Netherlands; 17 Department of Infectious-Tropical Diseases and Microbiology, IRCCS Sacro Cuore Don Calabria Hospital, Negrar di Valpolicella, 37024 Verona, Italy

**Keywords:** Oropouche emergence, liver, organoids, therapeutic discovery

## Abstract

Oropouche virus (OROV) is a neglected, re-emerging arbovirus that typically causes self-limiting febrile illness but can also lead to severe complications. With no approved vaccines or treatments available, we integrate clinical data with human liver-derived organoids to assess liver involvement in OROV infection and identify antiviral candidates through drug repurposing. Patient blood tests show elevated liver enzymes, indicating OROV-associated hepatic dysfunction. OROV isolates productively infect liver organoids and induce severe cellular damage. Transcriptomic profiling reveals strong virus-host interactions, including activation of interferon-stimulated genes and cell death pathways. Pharmacological inhibition of the interferon pathway enhances OROV replication, whereas treatment with therapeutic interferon-α suppresses the infection. Molnupiravir, a clinically approved antiviral drug targeting viral RNA-dependent RNA polymerase, markedly inhibits OROV replication and mitigates virus-induced cytopathology. Combining molnupiravir with interferon-α results in synergistic antiviral activity, indicating the complementarity of virus-targeted and host-directed strategies. These findings strengthen preparedness and response to OROV emergence.

## Highlights

• Elevated liver enzymes are detected in a subset of Oropouche patients
• OROV infects human liver organoids, triggering interferon response and cellular injury
• Molnupiravir strongly inhibits OROV infection and restores cellular homeostasis
• The combination of molnupiravir with interferon-alpha exhibits robust synergy

## Graphical abstract

**Figure:**
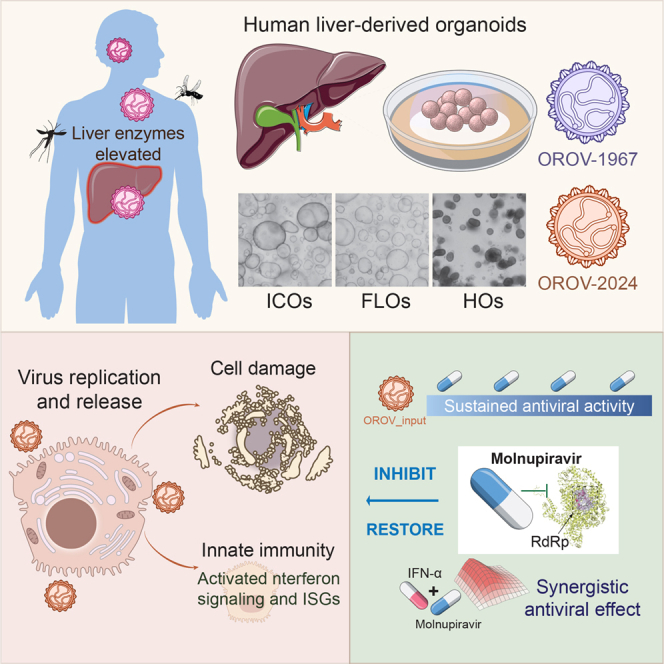

## Teaser

Li et al. integrate liver function data from Oropouche virus (OROV)-infected patients with human liver-derived organoids to demonstrate hepatic infection and resulting cellular injury. Using organoids, they further show that molnupiravir exerts strong antiviral activity against OROV and synergizes with interferon-alpha.

## Introduction

Oropouche virus (OROV) is a neglected vector-borne pathogen that causes Oropouche fever, first identified in Latin America and the Caribbean in 1955.^1^ In late 2023, it re-emerged in the Brazilian Amazon and quickly spread eastward, establishing transmission across all regions of Brazil by 2024.^2^^,^^3^^,^^4^ Endemic circulation has also been reported in Barbados, Bolivia, Colombia, Cuba, the Dominican Republic, Ecuador, Guyana, Panama, and Peru.^5^^,^^6^ Travel-related cases have been reported in Canada, the Cayman Islands, Germany, Italy, Spain, the Netherlands, and the United States.^7^^,^^8^^,^^9^ Oropouche fever is characterized by usually mild and self-limiting febrile illness. However, severe complications have been reported including neurological manifestations, pregnancy complications, vertical transmission associated with stillbirth and congenital anomalies, and death.^10^^,^^11^^,^^12^^,^^13^^,^^14^ As of July 2025, no licensed vaccines or antiviral drugs are available to prevent or treat OROV infection.

OROV is a tripartite, negative-sense RNA virus comprising three genome segments, namely, small (S), medium (M), and large (L). It is classified into *Orthobunyavirus oropoucheense* within the *Orthobunyavirus* genus, Peribunyaviridae family.^15^ OROV is primarily transmitted by *Culicoides paraensis* midges in an enzootic cycle involving pale-throat sloths (*Bradypus tridactylus*), non-human primates, and likely other wild mammals.^16^ In urban settings, a human-amplified transmission cycle may occur through midges.^17^ Additionally, mosquito species such as *Culex quinquefasciatus* may contribute to urban transmission, although further studies are needed to confirm their vector competence for OROV.^18^

After transmission through the bite of an infected arthropod, arboviruses enter the bloodstream and initially replicate in organs such as the liver and spleen and then spread to other tissues and organs.^19^ Previous studies have shown that OROV replicates efficiently in human hepatic cell lines *in vitro*.^2^^,^^20^ Similarly, experimental infections in hamster and mouse models revealed high OROV levels in the liver, accompanied by pronounced liver damage. OROV-triggered liver damage and dysfunction were defined by histopathological findings such as hepatocyte necrosis, Kupffer cell hyperplasia, and elevated levels of liver enzymes.^21^^,^^22^^,^^23^^,^^24^^,^^25^ Clinical evidence also links Oropouche fever to liver dysfunction. For example, jaundice, a condition commonly associated with hepatic impairment, has been documented in several cases.^26^^,^^27^ In 2024, two fatal cases without underlying health conditions showed elevated liver enzyme levels.^10^ Additionally, immunohistochemistry detected abundant OROV antigens in the liver of another fatal case.^28^ Despite these observations, how OROV infection affects the human liver remains poorly understood.

In this study, we analyzed liver enzyme levels in patients with OROV infection from the 2023–2024 outbreak and employed primary organoids derived from adult and fetal human livers to investigate OROV infection, cytopathogenesis, and potential antiviral therapeutics.

## Results

### Liver function tests indicate hepatic involvement in patients with Oropouche fever

To investigate potential liver involvement during OROV infection in humans, we analyzed liver function tests from six imported Oropouche cases in Europe.^8^^,^^9^^,^^29^^,^^30^ In this analysis, we assessed the levels of alanine aminotransferase (ALT), a marker for hepatocyte injury; gamma-glutamyl transferase (GGT), a marker for bile duct injury; and aspartate aminotransferase (AST), an enzyme highly expressed in both hepatocytes and cardiac muscle and whose elevation is generally considered a marker of liver injury when interpreted along with ALT.^31^ We found that 50% (3 of 6) exhibited elevated levels of both ALT and AST, whereas 1 of 3 patients tested showed elevated GGT levels (Figures S1A–S1C; Table S1).

**Figure 1:**
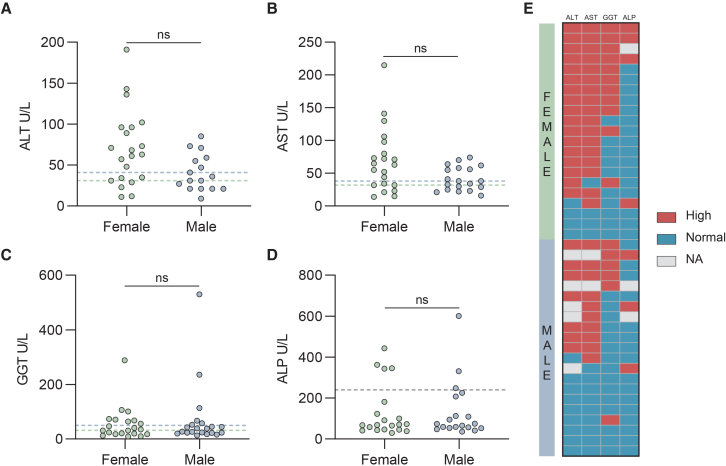
Liver function tests in a cohort of patients with OROV infection (A) ALT levels in OROV-infected patients recruited from a cohort in Brazil with a threshold (the upper reference limit) of 31 U/L in females (green dotted line) and 41 U/L in males (blue dotted line). (B) AST levels with a threshold of 32 U/L in females (female, green) and 38 U/L in males (male, blue). (C) GGT levels with a threshold of 32 U/L in females (female, green) and 50 U/L in males (male, blue). (D) ALP levels with a threshold of 240 U/L (gray dotted line). (E) A heatmap depicts elevated or normal liver enzymes in 42 enrolled patients based on the upper reference limit. The threshold references were determined by the kit manufacturers. NA, values not available. Data represent the values from individual patients; statistical analysis by Mann-Whitney U test; ns, not significant.

To further examine hepatic involvement during OROV infection, we evaluated serum levels of ALT, GGT, and AST in 42 patients with Oropouche fever confirmed by quantitative reverse-transcription PCR (RT-qPCR) during the 2024 outbreak in Brazil (Table S2).^2^ To minimize confounding factors, patients were matched by age, sex, and the time elapsed between symptom onset and sample collection. We found that 64.9% (24 of 37) of patients exhibited elevated levels of ALT (Figure 1A) and 67.5% (27 of 40) showed elevated levels of AST (Figure 1B). Next, the analysis was stratified by sex groups due to slight disparities in physiological ranges of liver enzymes between males and females.^32^ Among female patients, 81.0% (17 of 21) of cases exhibited elevated levels of either ALT or AST, with 76.2% (16 of 21) of cases showing concurrent elevations of both enzymes (Figures 1A and 1B). In male patients, 52.6% (10 of 19) presented higher levels of AST, and 43.8% (7 of 16) showed elevated levels of ALT (Figures 1A and 1B). Elevated GGT levels were observed in 52.3% (11 of 21) of female patients and 28.6% (6 of 21) of male patients (Figure 1C).

Furthermore, we also tested serum levels of alkaline phosphatase (ALP), a marker of liver damage or bone diseases.^33^ We found that 15.8% (3 of 19) of males and 20.0% (4 of 20) of females exhibited higher levels of ALP (Figure 1D). Bilirubin levels remained within the physiological range in both sexes (Figures S1D–S1F). Collectively, these findings demonstrate that patients with Oropouche fever exhibited elevated levels of key liver function markers (AST, ALT, GGT, and ALP), suggesting liver damage or dysfunction. Notably, female patients appeared to have a higher incidence of liver enzyme abnormalities compared to male patients (Figure 1E).

### OROV productively infects human liver-derived organoids

**Figure 2:**
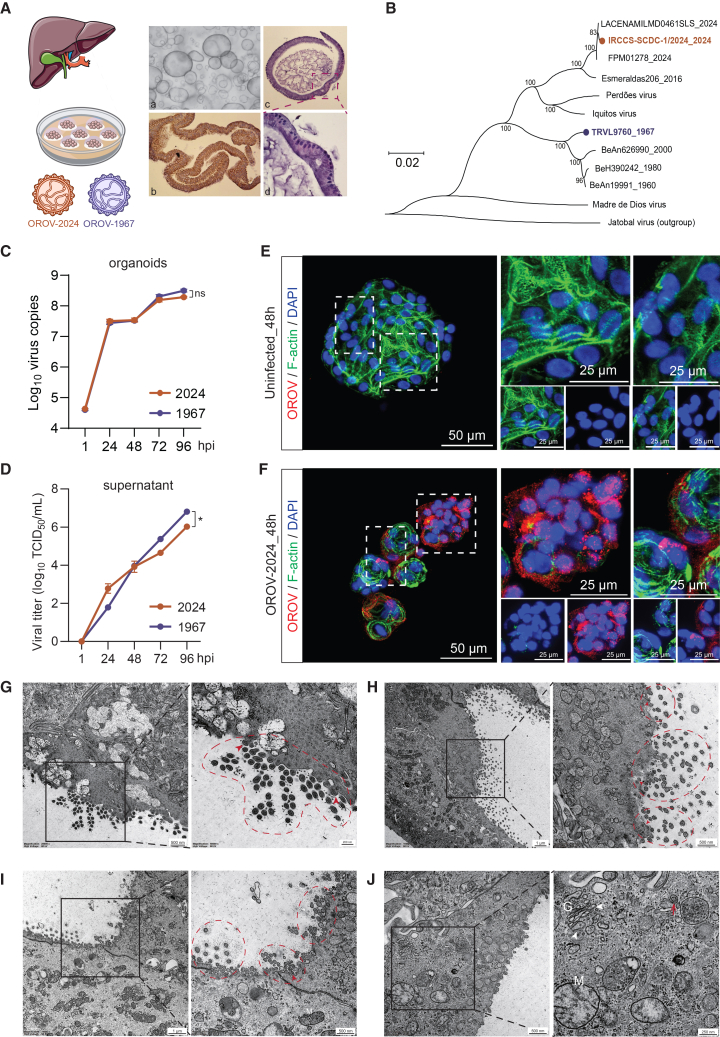
Productive infection of OROV in human liver-derived organoids (A) Schematic representation of adult human liver-derived organoids inoculated with OROV-2024 or OROV-1967. A representative bright-field image shows typical morphology of cultured ICOs (a). Hematoxylin and eosin (H&E) staining of an formalin-fixed paraffin-embedded (FFPE) 4-μm section reveals polarized epithelial architecture with a single-cell-layered structure (b). Immunohistochemistry staining for KRT19 confirms uniform expression of this cholangiocyte marker in organoids (c), with high-magnification detail shown in (d). (B) Phylogenetic analysis of the OROV L segment highlights the evolutionary divergence between the two strains. A neighbor-joining tree was constructed using the p-distance model, with branch support assessed by 1,000 bootstrap replicates. Only bootstrap values greater than 70% are shown. The tree is midpoint rooted. (C) Quantification of viral RNA levels in organoids post-inoculation with the two isolates (*n* = 4 biological replicates). (D) Quantification of infectious titers in culture medium (0 in *x* axis represents 1 h post-infection; *n* = 4 biological replicates). (E and F) Representative images of uninfected organoids (E) and organoids 48 h post-inoculation with OROV-2024 (F) by immunostaining with the antibody against OROV Gc glycoprotein (red), phalloidin for actin (green), and DAPI for nuclei (blue). Scale bars: 50 and 25 μm. (G–I) Representative TEM images of organoids 96 h post-inoculation with OROV-2024, showing extracellular mature OROV virions (red frames) and virions being secreted from clathrin-coated vesicles (red arrowheads). Scale bars: 500 nm and 200 nm (magnification) in (G), 1 μm and 500 nm (magnification) in (H and I). (J) Representative TEM images of organoids 96 h post-inoculation with OROV-2024, showing an immature OROV particle (red arrow) and modified Golgi complexes with swollen sacculi (white arrowheads). Scale bars: 500 nm and 250 nm (magnification). M, mitochondria; G, Golgi complex. Data represent mean ± SEM.; statistical analysis by mixed-effects model with restricted maximum likelihood (REML); ∗*p* < 0.05; ns, not significant.

To investigate whether human livers are susceptible to OROV infection, we utilized primary organoids cultured from adult human liver as an *in vitro* model. These 3D organoids are derived from residential stem cells/progenitors mainly located within the bile duct compartment of human livers and are named intrahepatic cholangiocyte organoids (ICOs).^34^^,^^35^ These organoids exhibited a polarized, epithelial structure with a single-cell layer and expressed the cholangiocyte marker KRT19, as shown by immunohistochemistry staining (Figure 2A). To assess the susceptibility of ICOs to OROV infection, we utilized two isolates, the historical TRVL9760 strain (OROV-1967) and the IRCCS-SCDC_1/2024 strain (OROV-2024) that represents the reassortant strain associated with the re-emergence of OROV in South America between 2023 and 2024 (Figures 2A, 2B, S2A, and S2B). Amino acid differences in the viral proteins encoded by L, M, and S segments between the two strains were also compared, with RNA-dependent RNA polymerase (RdRp) domain annotations based on the La Crosse virus polymerase structure (PDB 6Z6B) (Figures S2C–S2E).^36^

We first inoculated ICOs cultured from a female donor with both isolates and quantified OROV genomic RNA levels by RT-qPCR and infectious virus titers by 50% tissue culture infectious dose (TCID_50_) assay based on the induction of cytopathic effect (Figure S2F). For the OROV-2024 strain, intracellular virus genome copies showed a continuous increase from 4.6 log_10_ at 1 h post-inoculation to 8.3 log_10_ at 96 h (Figure 2C), while the fold change of RNA levels peaked at nearly 2,000 at 96 h (Figure S2G). Consistently, the titers of OROV infectious particles were undetectable at 1 h in the culture medium and reached 6 log_10_ TCID_50_/mL at 4 days post-inoculation (Figure 2D). Similar results were observed for the OROV-1967 strain (Figures 2C, 2D, and S2G), with OROV infectious titer reaching 6.8 log_10_ TCID_50_/mL at 4 days post-inoculation (Figure 2D). Overall, both strains robustly replicate in organoids, albeit showing subtle differences in the kinetics. We also confirmed the susceptibility of ICOs cultured from a male donor to OROV-1967 infection, by observing an increase of extracellular viral RNA copies into the culture medium (Figure S2H). We verified that both OROV-1967 and OROV-2024 strains replicated efficiently in human hepatoma-derived Huh7 cells, showing time-dependent increases in intracellular RNA levels (Figure S2I).

We further demonstrated viral replication of both OROV isolates by immunostaining the viral surface Gc glycoprotein in infected organoids at 48 h post-inoculation (Figures 2F and S2J), while no Gc signal was detected in the uninfected control group (Figure 2E). Next, our transmission electron microscopy (TEM) analysis of ICOs at 96 h post-OROV infection revealed many extracellular, mature virions near the plasma membrane, along with evidence of virion budding and release (Figures 2G–2I). These virions displayed the characteristic spherical morphology of orthobunyaviruses, with electron-dense cores and surface projections likely corresponding to the glycoprotein spikes. We also observed modified Golgi complexes with swollen sacculi and the presence of immature, annular-shaped OROV particles (Figure 2J). These intracellular and extracellular viral particles displayed morphological features similar to previously reported description of OROV.^37^^,^^38^ Notably, some extracellular virions exhibited uneven distribution of surface spikes, which may reflect dynamic glycoprotein organization during viral egress. Altogether, these findings demonstrate that human liver-derived organoids are susceptible to infection by both historical and contemporary OROV strains.

### OROV infection triggers a strong antiviral innate immune response in organoids

**Figure 3:**
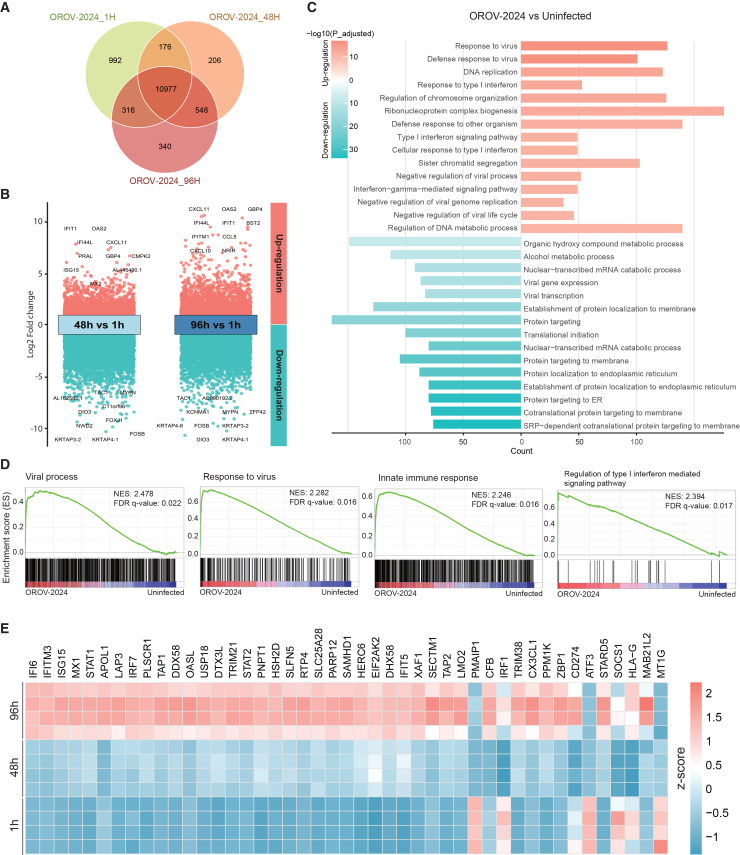
Transcriptomic analyses characterize OROV-host interactions (A) Venn diagram of overlapped differentially expressed genes in the OROV-2024 strain infected organoids at different time points. (B) Volcano plot analysis of differentially expressed genes at 48 and 96 h compared to 1 h after OROV-2024 inoculation. (C) Top 15 up-regulated and top 15 down-regulated pathways identified by Gene Ontology analysis in OROV-2024-inoculated organoids at 96 h post-inoculation compared to the uninfected controls cultured at the same condition for 96 h. (D) Gene set enrichment analysis (GSEA) in OROV-2024-infected organoids at 96 h post-inoculation compared to uninfected controls. (E) Heatmap showing the expression of selected ISGs in OROV-2024-infected organoids at 1, 48, and 96 h post-inoculation. ISGs were sourced from a published large-scale ISG screen^40^ and mapped to our RNA-seq dataset. Data are representative of four biological replicates from one RNA-seq experiment using two independent OROV isolates with multiple time points. The color scale represents row-wise *Z* score of gene expression across samples.

To investigate virus-host interactions in human liver cells, we inoculated ICOs with the two OROV strains and performed a genome-wide transcriptomic analysis. We set 1-h inoculation as a baseline control, as the eclipse period for OROV infection occurs around 3 h post-exposure.^39^ We mapped the host responses at 48 and 96 h post-inoculation when robust viral replication is established. A comparison of the differentially expressed genes among the different time points post-inoculation showed that at least 165 genes were expressed exclusively within each group, while slightly over 10,000 genes were shared among the three groups (Figures 3A and S3A). Volcano plot analysis demonstrated robust host responses to OROV infection at 48 and 96 h compared to 1 h (baseline), with over 5,000 of the genes significantly up- or down-regulated (Figures 3B and S3B). We found that many up-regulated genes are related to innate immune response such as *OAS2*, *IFIT1*, *IFI44L*, and *CXCL11*, which are interferon-stimulated genes (ISGs). These results are consistent for both OROV strains at 48 and 96 h post-inoculation. Furthermore, Gene Ontology analysis revealed that pathways related to response to virus and the interferon signaling pathway were significantly up-regulated, while several host transcriptional and protein targeting pathways were notably down-regulated (Figures 3C and S3C), suggesting the activation of innate antiviral defenses along with a broad suppression of host biosynthetic and metabolic functions. Additionally, gene set enrichment analysis revealed that transcriptional signatures related to viral process, antiviral response, innate immunity, and type I interferon were significantly enriched in organoids at 96 h post-inoculation with both OROV isolates compared to the uninfected controls cultured at the same condition for 96 h (Figures 3D, S3D, and S3E). To further characterize the interferon-mediated antiviral response, we curated a list of ISGs based on a large-scale ISG screen reported in a previous study.^40^ We then mapped these genes to our RNA sequencing dataset and selected those that were consistently detected across time points following OROV infection for visualization. To ensure comparability across conditions, the uninfected group was used as a baseline reference at each time point. Heatmap visualization revealed a distinct temporal pattern of ISG expression, with most genes exhibiting low basal levels at 1 h and progressively increasing expression at 48 and 96 h post-inoculation (Figures 3E and S3F). Notably, several representative ISGs including *IFI6*, *IFIT1*, *ISG15*, and *MX1* showed marked upregulation. Overall, these findings demonstrate that OROV replication elicits strong host responses in organoids, particularly activating innate immune pathways.

### OROV infection in organoids triggers severe cellular pathogenesis

**Figure 4:**
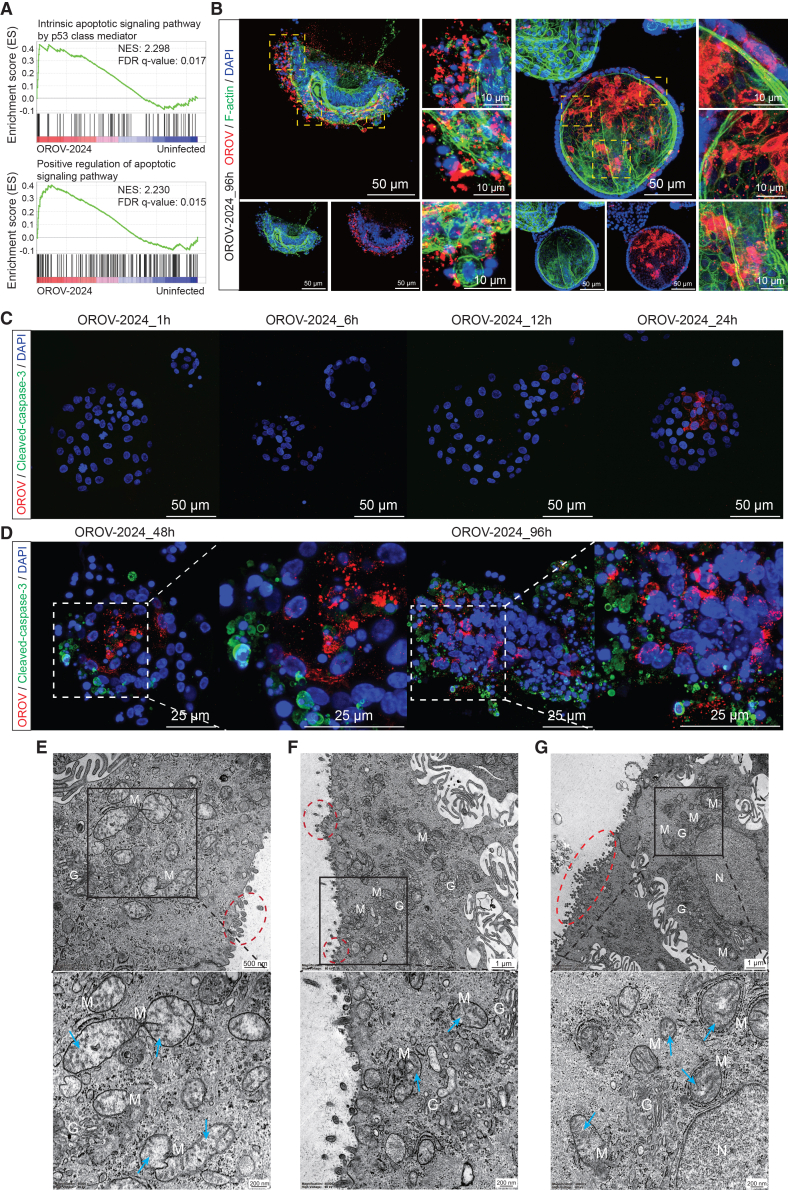
Cellular pathogenesis induced by OROV infection in organoids (A) GSEA analysis shows the activation of apoptotic signaling pathways in OROV-infected organoids at 96 h post-inoculation compared to the uninfected controls cultured at the same condition for 96 h. Data are representative of four biological replicates from one RNA-seq experiment. (B) Representative images of organoids 96 h post-inoculation with the OROV-2024 by immunostaining with the antibody against OROV Gc glycoprotein (red), phalloidin for F-actin (green), and DAPI for nuclei (blue). Scale bars, 50 and 10 μm. (C) Representative images of organoids at 1, 6, 12, and 24 h post-inoculation with OROV-2024 by immunostaining with the antibodies against OROV Gc protein (red), cleaved-caspase-3 (green), and DAPI (blue). Scale bars, 50 μm. (D) Representative images of organoids at 48 and 96 h post-inoculation with OROV-2024 by immunostaining with the antibodies against OROV Gc protein (red), cleaved-caspase-3 (green), and DAPI (blue). Scale bars, 25 μm. (E–G) Transmission electron microscopy images of OROV-infected organoids at 96 h post-inoculation, showing damaged mitochondria (M) with disorganized and disintegrating cristae (blue arrows), modified Golgi complexes (G), and extracellular viral particles (red circles). Insets show magnified views. Scale bars: 500 nm and 200 nm (E) and 1 μm and 200 nm (F and G). N, nucleus.

We next examined whether OROV infection in organoids activates cell death pathways to trigger severe cellular pathogenesis. Gene set enrichment analysis revealed significant upregulation of biological processes related to apoptotic signaling at 96 h post-inoculation compared to uninfected controls (Figure 4A). Depolymerization of the actin cytoskeleton is a hallmark of programmed cell death.^41^^,^^42^^,^^43^ Immunofluorescence staining of OROV-infected organoids at this time point revealed severe cytopathological changes in Gc-positive cells, including nuclear fragmentation, chromatin condensation, and actin cytoskeleton disassembly (Figures 4B and S4B), consistent with morphologies of severely damaged or late-stage apoptotic cells. Concurrently, we observed extensive extracellular viral signals indicating viral release upon cellular damage. Such patterns were absent in uninfected organoids (Figure S4A).

To investigate the temporal dynamics of OROV infection and cell death, we performed immunostaining at multiple time points from 1 to 96 h post-inoculation. Signals for the OROV Gc protein became detectable by 12 h and increased markedly by 24 h, while staining for the apoptotic marker cleaved caspase-3 remained absent during this early phase (Figure 4C). By 48 and 96 h post-inoculation, Gc protein was broadly distributed throughout the infected organoids, indicating robust viral replication. Interestingly, cleaved caspase-3 signals predominantly localized to the periphery of the organoids and were frequently associated with fragmented nuclei, consistent with apoptotic cell death. Notably, viral and apoptotic markers did not always co-localize, potentially reflecting viral release from disintegrating cells.

Electron microscopy further confirmed organelle damage in infected organoids at 96 h post-inoculation. Infected cells showed swollen mitochondria with disorganized and disintegrating cristae, as well as modified Golgi complexes and extracellular viral particles (Figures 4E–4G). However, organoids with moderate or lower levels of infection retained relatively intact cellular morphology (Figures 4B and S4C–S4F).

### Molnupiravir potently inhibits OROV replication

**Figure 5:**
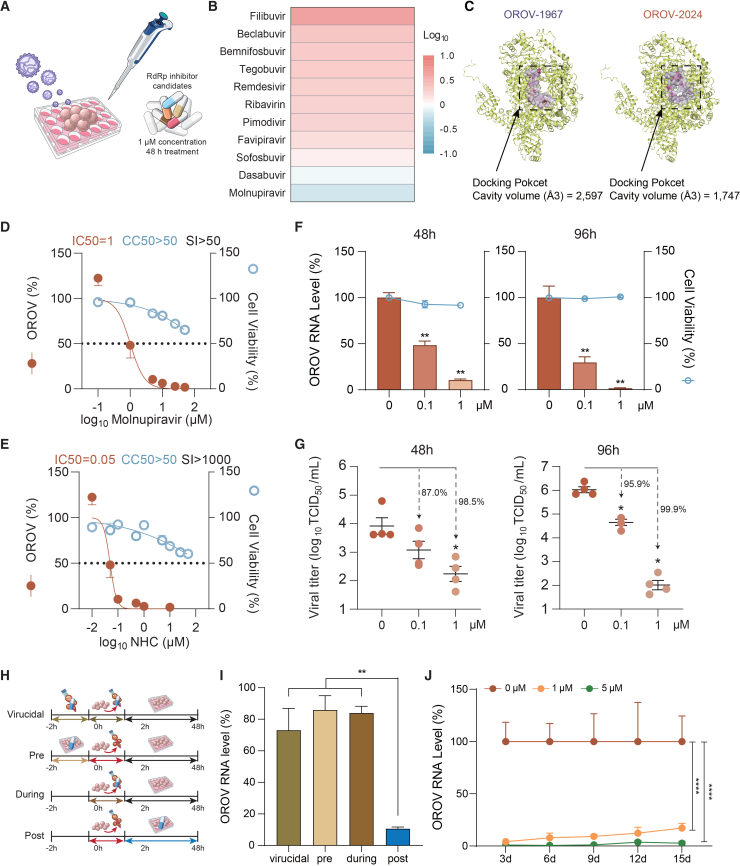
Antiviral drug profiling identified molnupiravir as a potent inhibitor of OROV infection (A) Schematic diagram of profiling RdRp inhibitors, prioritizing clinically used drugs, in OROV-infected liver organoids. (B) Fold changes of OROV RNA levels in infected organoids after 48-h treatment with the selected RdRp inhibitors. (C–E) (C) Potential docking pocket of RdRp protein predicted by CurPocket. RNA-protein contact active site cavity is indicated as a dotted circle (D and E). Dose-response curves of molnupiravir (D) and NHC (E) in infected organoids, showing the 50% inhibitory concentration (IC_50_) and 50% cytotoxic concentration (CC_50_) values (*n* = 4 biological replicates). (F) Intracellular OROV RNA levels (*n* = 5 biological replicates) and corresponding cell viability (*n* = 8 biological replicates) following 48- and 96-h treatment with NHC (0.1 or 1 μM). (G) Infectious viral titers in culture supernatant from organoids treated with NHC at 48 and 96 h (*n* = 4 biological replicates). (H) Schematic of the time-of-addition experiment to assess the timing of NHC-mediated antiviral activity. (I) OROV RNA levels under virucidal treatment (virucidal, *n* = 7 biological replicates), pre-treatment (pre, *n* = 6 biological replicates), treatment during virus inoculation (during, *n* = 7 biological replicates), and post-treatment conditions (post, *n* = 5 biological replicates). Data are shown as relative levels compared to matched untreated controls in each group. The post-treatment group is same as the condition of 48-h treatment with 1 μM concentration in (F). (J) OROV RNA levels in the supernatant of cultured organoids without or with NHC treatment (1 or 5 μM) over a 15-day period. Data are shown as relative levels normalized to untreated controls (*n* = 5 biological replicates). Statistical comparisons between treatment groups were performed using two-tailed Mann-Whitney U tests (F, G, and I). Statistical analysis for longitudinal data were performed using mixed-effects model (REML) (J). Data represent mean ± SEM; ∗*p* < 0.05, ∗∗*p* < 0.01, ∗∗∗∗*p* < 0.0001.

RdRp is responsible for the replication of RNA viruses and is an important antiviral target.^44^ To meet the urgent clinical needs, we aimed to identify effective anti-OROV candidates by profiling and repurposing the clinically used RdRp inhibitors (Figure 5A). To this end, we screened a panel of 11 RdRp inhibitors in ICOs infected with the OROV-1967 isolate. To minimize cytotoxic effects on host cells, we used a relatively low concentration (1 μM) and treated for 48 h. Among the tested compounds, only molnupiravir demonstrated effective inhibition of viral replication at this concentration (Figure 5B). Molnupiravir is an orally administered pro-drug of the ribonucleoside analog β-*d*-N4-hydroxycytidine (NHC), which is taken up into cells and phosphorylated to a competitive substrate for viral RdRp, resulting in accumulation of errors in the viral genome and inhibition of viral replication.^45^^,^^46^^,^^47^

To map the site-specific binding modes of molnupiravir, NHC, and NHC-5′-triphosphate (Figure S5A), we predicted the RdRp structures (encoded by the L segment) of both strains using AlphaFold3,^48^ achieving a high predicted template modeling score (pTM = 0.83), indicating high structural accuracy (Figure S5B). Next, we predicted the potential docking pockets within the RdRp structures (Figures 5C and S5C). Molecular docking analysis revealed that molnupiravir, NHC, and NHC-5′-triphosphate bind to OROV RdRp (Figures S5D–S5J). Consistently, NHC-5′-triphosphate, the active component of molnupiravir and NHC,^49^ exhibited a lower Vina score (−7.9) (Figures S5F and S5J), suggesting a more stable and stronger interaction with OROV RdRp.^50^ To further support these findings, we performed comparative docking analysis of NHC-5′-triphosphate to the crystal structure of the SARS-CoV-2 RdRp (PDB: 7OZV) (Figure S6A).^51^ As expected, ATP and GTP were found to engage the same catalytic residues predicted to interact with NHC-5′-triphosphate (Figures S6B and S6C). Comparative docking of all tested compounds (Figure 5B) revealed that although NHC-5′-triphosphate did not have the strongest predicted binding score, it showed the highest number of predicted interactions with catalytic residues within the NTP-binding pocket. A few other compounds with stronger predicted affinities bound outside the catalytic region did not show antiviral activity in OROV-infected organoids (Figures S7 and 5B). These results indicate that the superior antiviral efficacy of NHC observed in organoids did not directly correlate with docking scores, highlighting the importance of integrating structural predictions with functional validation.

Subsequently, we evaluated a range of concentrations (0.01–50 μM) of molnupiravir and NHC in organoids infected with OROV. Both compounds exhibited dose-dependent inhibition of viral replication of the OROV-2024 isolate, although higher concentrations (e.g., 10–50 μM) moderately affected organoid growth. The half-maximal inhibitory concentration (IC_50_) values were determined to be 1 μM for molnupiravir and 0.05 μM for NHC (Figures 5D and 5E). Similar results were observed with the OROV-1967 strain, where both compounds demonstrated dose-dependent antiviral activity. The IC_50_ values for molnupiravir and NHC against this strain were 2.2 and 0.05 μM, respectively (Figures S8A and S8B).

Next, we determined the time- and dose-dependent antiviral activity of the active drug NHC in OROV-infected ICOs. Treatment with 0.1 and 1 μM NHC for 48 and 96 h significantly reduced intracellular viral RNA levels (*n* = 4, *p < 0.01*), with no measurable cytotoxicity observed at either concentration (Figure 5F). At 1 μM, RNA levels were reduced by ∼90% at 48 h and ∼98% at 96 h. Consistently, infectious virus production was also strongly inhibited: 1 μM NHC led to 98.5% and 99.9% reduction in viral titers at 48 and 96 h (*n* = 4, *p < 0.05*), respectively (Figure 5G). To further understand the mode of action of NHC, we performed a time-of-addition assay (Figure 5H).^52^ NHC treatment was most effective when administered 2 h post-inoculation with OROV, whereas virucidal treatment, pre-treatment, or treatment during virus inoculation resulted in minimal inhibition (Figure 5I). To assess the effects of prolonged treatment, OROV-infected organoids were continuously exposed to 1 or 5 μM NHC for up to 15 days. Both concentrations maintained robust viral suppression without clear evidence of viral rebound during the treatment period (Figure 5J). Subsequently, we performed an additional 15-day regimen using a stepwise dose-escalation approach, starting at 0.01 μM and gradually increasing to 10 μM. Quantification of viral RNA levels in the supernatant showed progressive inhibition in parallel with the increasing NHC concentrations (Figure S8C).

### Molnupiravir treatment partially restores host cellular homeostasis

**Figure 6:**
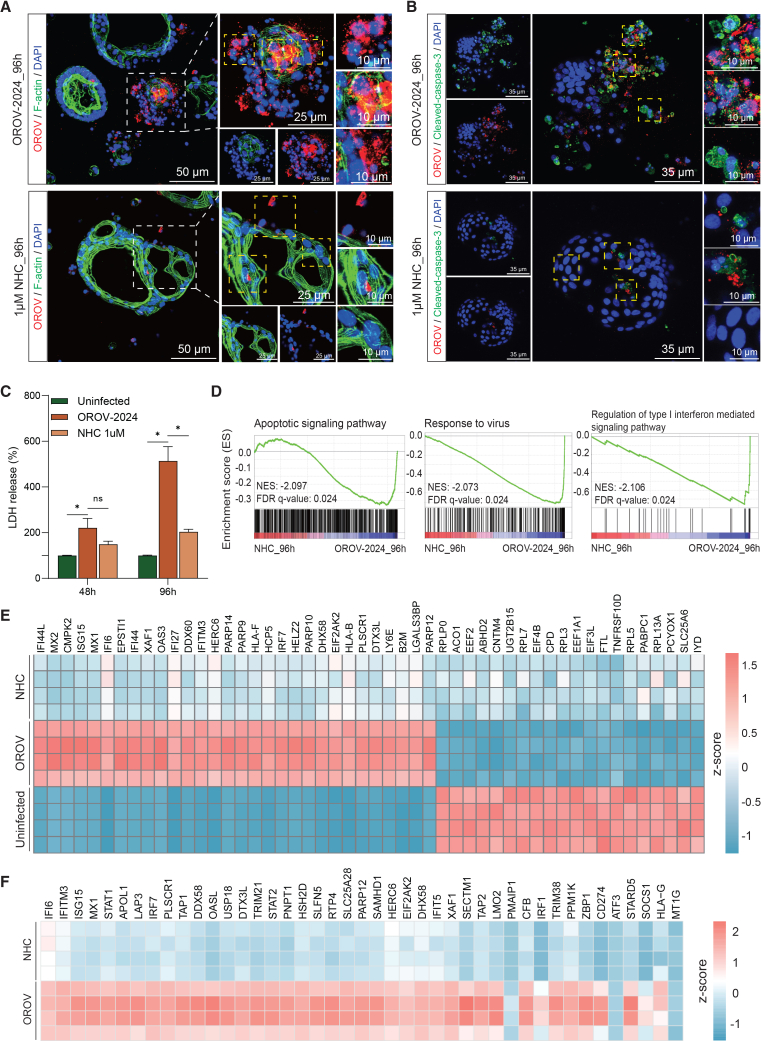
Molnupiravir attenuated virus-induced cell death and modulates host antiviral responses in OROV-infected liver organoids (A) Representative images of OROV-2024-infected organoids without (top) or with (bottom) 96-h NHC (1 μM) treatment by immunostaining with the antibody against OROV Gc glycoprotein (red), phalloidin for F-actin (green), and DAPI for nuclei (blue). Scale bars: 50 , 25, and 10 μm. (B) Representative images of OROV-2024 infected organoids without (top) or with (bottom) 96-h NHC (1 μM) treatment by immunostaining with the antibody against OROV Gc glycoprotein (red), cleaved-caspase-3 (green), and nuclei (DAPI, blue). Scale bars: 35 and 10 μm. (C) LDH release in culture supernatant of organoids at 48 and 96 h post-inoculation, comparing uninfected, OROV-infected, and NHC-treated groups (1 μM) (*n* = 4 biological replicates). (D) GSEA in OROV-infected organoids with NHC treatment for 96 h compared to organoids with OROV infection alone. (E) Heatmap of the top 50 significantly regulated genes comparing uninfected controls, OROV-infected organoids at 96 h post-inoculation, and OROV-infected organoids with 96-h NHC treatment. (F) Heatmap of selected ISGs comparing OROV-infected organoids at 96 h post-inoculation and OROV-infected organoids with 96-h NHC treatment. ISGs were sourced from a published large-scale ISG screen^40^ and mapped to our RNA-seq dataset. The color scale represents row-wise *Z* score of gene expression across samples. Data are representative of four biological replicates from one RNA-seq experiment (D–F). Data represent mean ± SEM; statistical analysis by Mann-Whitney U test; ∗*p* < 0.05; ns, not significant.

Immunofluorescence staining showed a clear reduction of viral Gc glycoprotein in NHC-treated organoids, confirming effective inhibition of OROV replication (Figure 6A). NHC treatment also rescued virus-induced cytopathic changes including extensive actin cytoskeleton disruption and nuclear fragmentation. Cleaved-caspase-3 staining further confirmed that NHC treatment mitigated virus-induced apoptotic and lytic cell death at both 48 h and 96 h post-inoculation (Figures 6B, S8C, and S8D). Lactate dehydrogenase (LDH) release assays, which measure membrane damage associated with cell death, showed a similar trend, with NHC treatment significantly lowering virus-induced cytotoxicity at both 48 and 96 h (Figure 6C). Transcriptomic analysis showed that NHC treatment for 96 h substantially alleviated rewiring of the host transcriptome by OROV infection and reversed the activation of viral response and apoptotic signaling pathways, while restoring the balance in host immune responses (Figures 6D, 6E, and S8E). Notably, OROV-mediated activation of multiple ISGs was attenuated upon treatment (Figure 6F), suggesting restoration of host cellular homeostasis. We also found that OROV infection disturbed the transcription of mitochondrial-related genes, including upregulation of genes involved in apoptosis and mitophagy, as well as dysregulation of genes essential for mitochondrial respiration and morphology dynamics. These abnormalities were largely restored by NHC treatment (Figure S8F). Collectively, these findings provide preclinical evidence supporting the potential of molnupiravir as an antiviral therapeutic candidate for treating OROV infection.

### Human fetal liver-derived organoids are susceptible to OROV infection but potently inhibited by molnupiravir

Given that OROV RNA was detected in liver tissue of a stillborn infant caused by vertical transmission during the 2023–2024 outbreak,^53^ we assessed viral susceptibility of primary organoids cultured from human fetal liver. These organoids, which also carry a cholangiocyte phenotype,^54^ were then inoculated with the OROV-2024 isolate (Figure S9A). Intracellular and extracellular viral genome copy numbers increased by 7.7 log_10_ and 4 log_10_ at 3 days compared to 1 h post-OROV infection, respectively (Figures S9B and S9C). Subsequently, we evaluated the antiviral activity of NHC, the active form of molnupiravir, in OROV-infected fetal organoids. Treatment with 1 μM NHC for 72 h resulted in 80% inhibition of intracellular (*p < 0.05*), with no measurable cytotoxicity observed (Figure S9D), and a 96.9% reduction in extracellular viral titers in the culture supernatant (*p < 0.05*) (Figure S9E). Consistently, immunofluorescence staining showed substantial reduction of Gc glycoprotein in NHC-treated organoids compared to the untreated group, confirming the potent antiviral effect of NHC against OROV infection (Figures S9F–S9H). These data demonstrate that human fetal liver-derived organoids are susceptible to OROV infection, which can be effectively inhibited by NHC treatment.

### Hepatocyte-differentiated organoids support OROV replication and respond to molnupiravir treatment

To further confirm our findings in a liver-relevant model, we differentiated ICOs into hepatocyte-like organoids using a well-defined protocol (Figure S10A).^35^^,^^54^ After a 15-day process of differentiation, the resulting organoids showed increased expression levels of the hepatocyte markers albumin and CYP3A4, while the expression level of the progenitor cell marker LGR5 decreased, acquiring hepatocyte morphology of polygonal cell shapes (Figure S10B). Hepatocyte-differentiated organoids supported robust replication of the OROV-2024 strain, with viral RNA copy numbers increasing from 5.8 log_10_ at 1 h post-inoculation to 7.5 log_10_ at 72 h in organoids and from 2.7 log_10_ at 1 h post-inoculation to 6.1 log_10_ at 72 h in culture supernatants (Figures S10C and S10D). Treatment with 1 μM NHC significantly inhibited intracellular viral RNA levels by 80.4% without affecting cell viability and reduced infectious virus titers in the supernatant by 81.1% (Figures S10E and S10F). NHC treatment also reduced both OROV Gc protein and cleaved-caspase-3 signals in infected hepatocyte-differentiated organoids and attenuated LDH release (Figures S10G and S10H). These findings demonstrate that OROV can productively infect and replicate in hepatocyte-differentiated organoids and further confirm the efficacy of molnupiravir in inhibiting viral replication and virus-induced cell damage.

### Harnessing host antiviral interferon response to develop molnupiravir-based combination therapy

**Figure 7:**
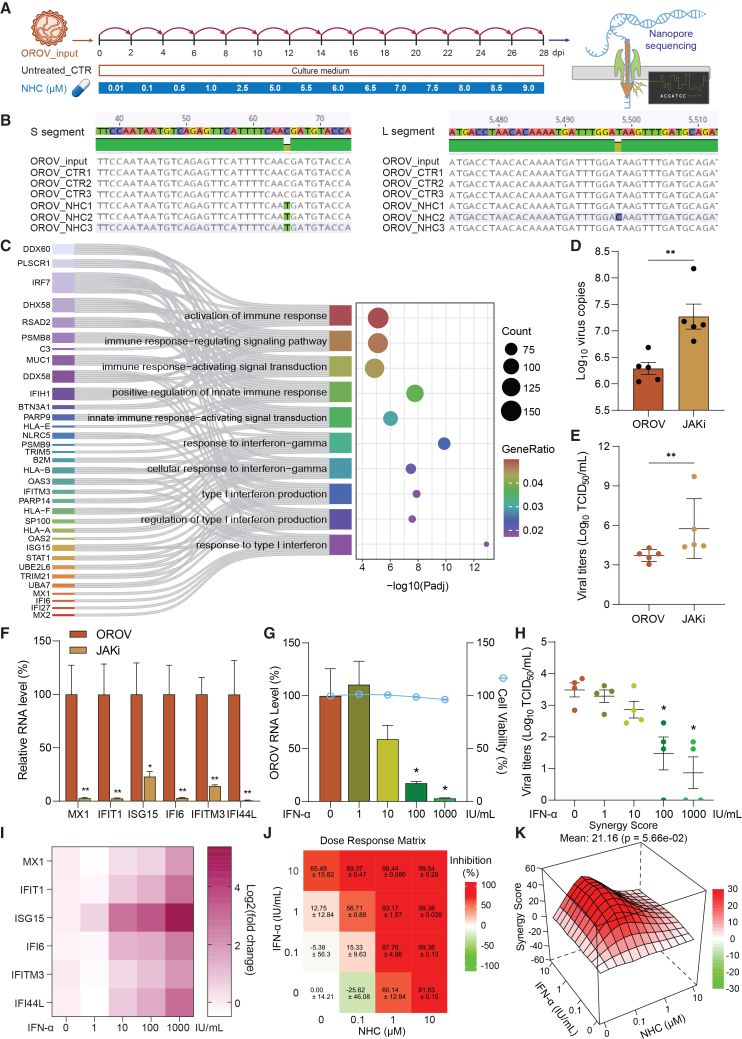
Harnessing host interferon response for developing molnupiravir-based antiviral combination (A) Schematic illustration of serial passaging with (*n* = 3 biological replicates) or without (*n* = 3 biological replicates from one passaging experiment series) NHC treatment of escalating concentrations in Vero cells. (B) Identification of mutations by whole-genome sequencing of OROV using Nanopore sequencing technology. (C) Sankey-bubble plot showing significantly enriched immune and interferon-related pathways in OROV-infected human adult liver-derived organoids at 96 h post-inoculation. Data are representative of four biological replicates from one RNA-seq experiment. (D) Quantification of OROV RNA levels in organoids with or without JAK inhibitor treatment (*n* = 5 biological replicates). (E) Infectious viral titers in culture medium from organoids treated with or without JAK inhibitor (*n* = 5 biological replicates). (F) Relative expression of selected ISGs in OROV-infected organoids treated with or without 10 μM JAK inhibitor (*n* = 4 biological replicates). (G) Intracellular OROV RNA levels and corresponding cell viability following IFN-α treatment for 48 h with increasing concentrations (0, 1, 10, 100, 1,000 IU/mL) (*n* = 4 biological replicates). (H) Infectious viral titers in culture medium following IFN-α treatment (*n* = 4 biological replicates). (I) Heatmap showing induced expression of representative ISGs after 48-h treatment of IFN-α at different concentrations. Values represent log_2_(fold change) relative to untreated controls. (J and K) Dose-response matrix (J) and ZIP synergy landscape (K) of IFN-α and NHC combination treatment in OROV-infected organoids, generated by SynergyFinder 3.0. Data represent mean ± SEM; statistical analysis by two-tailed Mann-Whitney U test unless otherwise indicated; ∗*p* < 0.05, ∗∗*p* < 0.01.

Given the general concern that direct-acting antivirals such as molnupiravir may induce drug-resistance mutations,^55^ we analyzed the genomic sequences of OROV following serial passaging in Vero cells with or without escalating concentrations of NHC treatment (Figure 7A). By Nanopore-based whole-genome sequencing, one mutation was found in the consensus sequence of one treated sample (T5498C, segment L, nonsynomynous) and one mutation was found in the consensus sequence of all three treated samples (C66T, segment S, nonsynomynous) (Figure 7B). However, both mutations were present already as minor variant in either the input or in the control passaged viruses (Table S3). Furthermore, given that both are nonsynomynous mutations, there are no clear functional implications. In addition, a deletion of three nucleotides (N621Δ, segment M) was found in all cultured viruses which might be a culture-based adaption since it was not found in the input virus stock.

Although our findings suggest a relatively high barrier to resistance development, combining antivirals with complementary mechanisms remains a common clinical strategy to enhance efficacy and reduce the risk of drug resistance. Transcriptomic analysis revealed robust activation of interferon responses, indicated by the induction of a panel of ISGs, in organoids infected with OROV for 96 h (Figure 7C). Pharmacological inhibition of the interferon pathway using a JAK inhibitor markedly increased viral RNA levels and infectious viral titers (Figures 7D and 7E) while suppressing ISG expression (Figure 7F). These findings demonstrate that the endogenous interferon response plays a functional role in restricting OROV replication. Conversely, treatment with recombinant interferon-alpha (IFN-α), a clinically approved therapy for several viral infections,^56^ dose-dependently inhibited intracellular viral RNA level (Figure 7G) and infectious virus production (Figure 7H), accompanied by ISG induction (Figure 7I).

Finally, to leverage complementary antiviral mechanisms, we tested the combination of NHC and IFN-α in OROV-infected organoids. A range of concentrations was applied individually or in combination, followed by viral RNA quantification and synergy modeling using the SynergyFinder 3.0 tool.^57^ The analysis revealed a strong synergistic antiviral effect, indicated by a high synergy score (mean = 21.16) (Figures 7J and 7K). These findings support a combination strategy that integrates virus-targeting and host-directed antiviral approaches to achieve more effective control of OROV infection.

## Discussion

Previous experimental studies in murine models demonstrated that OROV infection causes significant liver damage, especially targeting hepatocytes.^21^^,^^22^^,^^23^^,^^24^^,^^25^ Our study provides clear evidence that OROV infection can induce hepatic alterations in patients with Oropouche fever. Elevated liver transaminases (ALT, AST, and GGT) observed in some patients point to possible injury of both hepatocyte and cholangiocyte compartments.^31^ We hypothesize that OROV’s liver tropism may be linked to high expression of low-density lipoprotein receptor-related protein 1 (LRP1) in liver cells,^58^^,^^59^ which has been proposed as an OROV entry factor.^60^ Notably, similar mechanisms of LRP1-dependent liver tropism have been observed in Rift Valley fever virus, a related tri-segmented RNA virus.^61^^,^^62^

We demonstrated that human liver organoids derived from both fetal and adult livers support productive OROV infection. The cholangiocyte phenotype of these organoids corresponds with elevated GGT levels observed in some patient cases. In addition to cholangiocyte-like ICOs, we showed that hepatocyte-differentiated organoids^35^ are also permissive to OROV and exhibit virus-induced cytopathic effects. Following infection, viral loads progressively increased, accompanied by clear signs of cell injury, including cytoskeletal disassembly, membrane damage, LDH release, and cleaved caspase-3, consistent with a lytic infection cycle. While these cellular responses represent injuries at the epithelial cell level rather than organ pathology, clinical observations have also shown that liver involvement can occur during OROV infection. A recent autopsy report described abundant viral antigens in the liver together with severe hepatic injury.^28^ In addition, transcriptomic profiling revealed activation of apoptotic pathways and engagement of host antiviral responses, reflecting a strong inflammatory state that likely acts together with direct viral damage to drive cellular- and tissue-level injury. These findings point to the need for targeted therapeutic strategies to mitigate the OROV-induced severe consequences.

We discovered that molnupiravir potently inhibits OROV infection in human liver-derived organoids. Originally developed for influenza, molnupiravir was later repurposed for COVID-19 treatment in several countries.^47^^,^^63^ Previous studies have shown its broad-spectrum antiviral activity against SARS-CoV-2^64^ and seasonal coronaviruses.^52^ As a prodrug, molnupiravir is metabolized into its active form, NHC, which inhibits viral RNA replication.^65^ Its antiviral mechanism involves incorporation of NHC ribonucleoside into viral RNA by RdRp, leading to mispairing with cytidine or uridine, inducing transition mutations and suppressing viral replication.^47^^,^^51^ Consistent with this mode of action, our data showed that molnupiravir treatment after OROV inoculation significantly reduced viral replication in liver organoids, with even greater inhibition observed using the active NHC metabolite. The time-of-addition analysis^52^ revealed that NHC was most effective when administered post-infection, whereas pre-treatment, co-treatment during inoculation, or direct incubation with virus particles (virucidal) had limited effects. These findings support its role as an RdRp inhibitor targeting OROV replication. Notably, molnupiravir/NHC treatment also alleviated OROV-induced cytopathology and apoptotic cell death.

One potential concern with molnupiravir is its ability to introduce mutations into viral RNA, which could increase the risk of drug resistance, especially during prolonged treatment. However, since Oropouche fever is typically an acute, self-limiting illness with symptoms lasting 2–7 days,^12^ antiviral therapy would likely be short term, prescribed for only a few days. In our liver organoid models, both short-term (2–4 days) and extended (15-day) treatment with molnupiravir effectively suppressed OROV replication without clear evidence of resistance. Additionally, Nanopore sequencing of the complete OROV genome after continuous passaging under NHC pressure in Vero cells identified two mutations. However, both were nonsynonymous without clear functional implications, suggesting no evidence for the emergence of drug-resistant variants in this experimental setup. Nevertheless, the potential risk of resistance emergence should be thoroughly assessed in future studies. Lessons from real-world use of molnupiravir for COVID-19, even with standard 5-day regimen, show that a distinct drug-associated mutational signature has appeared in global SARS-CoV-2 genomes.^55^ Therefore, if molnupiravir advances to clinical use for treating Oropouche, careful monitoring of viral evolution in treated individuals as well as in the broader population will be essential to detect and prevent the emergence of resistant variants.

In clinical settings, the maximum serum concentration of NHC, the active metabolite of molnupiravir, can reach approximately 8,000 ng/mL (∼30 μM).^65^ In our study, NHC inhibited OROV replication with an IC_50_ value as low as 0.05 μM, and we confirmed potent antiviral activity at concentrations of 0.1, 1, and 5 μM. Consistent with a previous study,^66^ we also demonstrated that ribavirin, a commonly used antiviral, lacks efficacy against OROV. In addition to direct-acting antivirals, we also evaluated host-directed strategies based on the interferon response. While pre-treatment with IFN-α has shown some anti-OROV activity in mouse models, post-infection administration was not effective.^67^ In human liver-derived organoids, we found that OROV infection induced robust interferon responses which functionally restrict viral replication. Importantly, treatment with IFN-α significantly inhibited OROV infection and the combination of molnupiravir with IFN-α resulted in strong synergistic antiviral activity. These findings highlight the strong potential of repurposing molnupiravir, and its combination with host-directed antivirals such as IFN-α, for OROV treatment. However, our antiviral evidence is primarily based on organoid-based experiments. Further *in vivo* studies are recommended to confirm the definitive therapeutic efficacy and pharmacodynamics of these agents. Clinical safety data on molnupiravir use during pregnancy remain unavailable,^47^ and potential risks should be carefully evaluated before clinical application.

In summary, our study showed the liver as a key target organ during OROV infection in humans. We demonstrated that primary organoids derived from adult and fetal human livers are highly permissive to OROV, leading to severe cellular pathology and establishing a robust, physiologically relevant *in vitro* model for OROV research. OROV infection elicits a strong antiviral interferon response, and treatment with IFN-α effectively inhibits viral replication. Finally, we identified molnupiravir as a promising antiviral candidate against OROV, and its combination with IFN-α exerts strong synergistic effects. These findings provide a framework for further *in vivo* studies to enable targeted interventions and rapid responses to this emerging public health threat.

### Limitations of the study

This study has several limitations. First, available liver function data were limited to patients with mild OROV infections and lacked information on pre-existing liver conditions. Liver biopsies from patients with severe or fatal cases are needed for a comprehensive understanding of OROV-associated liver pathology. Second, while we used both historical and recent viral strains, the study was not designed to compare them. A rigorous comparison would require a larger panel of strains and a dedicated study design. Third, our liver organoids consist solely of epithelial cells and lack blood flow and multicellular tissue architecture; therefore, they model cellular injury rather than organ-level pathology. Incorporating immune components in future models will be essential to elucidate the immunopathogenesis of OROV-induced liver injury. Additionally, although organoids from both male and female donors were used, the study was not specifically designed to analyze sex-specific differences in infection and treatment responses. Fourth, while our *in vitro* data demonstrate strong antiviral activity of molnupiravir against OROV, *in vivo* studies are necessary to validate its efficacy. Finally, the precise mechanism by which molnupiravir inhibits OROV replication remains to be fully elucidated.

## Resource availability

### Lead contact

Further information and requests for resources and reagents should be directed to and will be fulfilled by the lead contact, Qiuwei Pan (q.pan@erasmusmc.nl).

### Materials availability

No novel, unique reagents were developed as part of this research.

### Data and code availability

• Data: RNA sequencing data have been deposited to in the National Center for Biotechnology Information (NCBI) Gene Expression Omnibus (GEO) database (GEO: GSE316780). The accession number is also listed in the key resources table.
• Code: This paper does not report original code.
• Other items: Any additional information required to reanalyze the data reported in this work paper is available from the lead contact (Qiuwei Pan, q.pan@erasmusmc.nl) upon request.

## Acknowledgments

Q.P. is supported by a VIDI personal grant (no. 91719300) from the Netherlands Organisation for Health Research and Development (ZonMw). W.M.d.S. is supported by Burroughs Wellcome Fund—Climate Change and Human Health Seed Grants (grant number 1022448) and a Wellcome Trust—Digital Technology Development Award in Climate Sensitive Infectious Disease Modeling (grant number 226075/Z/22/Z). J.L.P.-M. is supported by the São Paulo Research Foundation (grant number 2022/10442-0) and National Council for Scientific and Technological Development (grant number 309971/2023-3). We are grateful to the staff of the CASA clinics in Leiden and Rotterdam for collecting fetal tissues. We thank Dr. Ron Smits from the Department of Gastroenterology and Hepatology, Erasmus MC, the Netherlands, for providing technical assistance. We thank Dr. Sven Reiche from Federal Research Institute for Animal Health, Germany, for providing the anti-OROV Gc antibody and Dr. Chantal Reusken from the National Institute for Public Health and the Environment, the Netherlands, for providing the OROV strain TRVL9760.

## Author contributions

J.L. and X.W., conceptualization, methodology, formal analysis, investigation, data curation, writing – original draft, writing – review & editing, and visualization; Y.D. and F.Q., methodology, formal analysis, investigation, data curation, writing – original draft, writing – review & editing, and visualization; S.T.S.d.L., methodology, formal analysis, investigation, data curation, writing – review & editing, and visualization; L.P., methodology, formal analysis, investigation, and writing – review and editing; R.S., M.M.A.V., and M.B., methodology and investigation; J.F., I.M.C., X.H., and L.M.S.M., investigation and formal analysis; D.M.O., M.P.P., A.B., E.P., H.L.A.J., J.A.T.S., M.N.N.d.S., E.C.P., J.L.P.-M., P.L., A.A.A., L.J.W.v.d.L., and C.C., investigation; B.B.O.M., methodology, formal analysis, investigation, and data curation; W.M.d.S. and W.W., conceptualization, methodology, resources, writing – review & editing, and supervision; Q.P., conceptualization, resources, writing – original draft, writing– review & editing, supervision, project administration, and funding acquisition.

## Declaration of interests

The authors declare no competing interests.

## STAR★Methods

### Key resources table

**Table:** 

REAGENT or RESOURCE	SOURCE	IDENTIFIER
**Antibodies**		

KRT19	Agilent Dako	Cat#GA61561-2
Envision Flex HRP	Agilent Dako	Cat#GV90011-2
Anti-Oropouche orthobunyavirus Gc (1:100, mouse monoclonal antibody; clone 2B5B1)	provided by Dr. Sven Reiche from Federal Research Institute for Animal Health, Germany	N/A
Human Active Caspase-3 Antibody	Bio-Techne	Cat#AF835; AB_2243952
anti-mouse immunoglobulin G (IgG) (H + L, Alexa Fluor 594)	Invitrogen	Cat#A32742; AB_2762825
Phalloidin (Alexa Fluor 488)	Invitrogen	Cat#A12379
Antifade Mounting Medium with DAPI	Vector Laboratories VECTASHIELD	Cat#H-1200-10

**Bacterial and virus strains**		

Oropouche virus, human, strain RIVM/OROV/TRVL9760/09/11/1967	the National Institute for Public Health and the Environment, The Netherlands	N/A
OROV strain IRCCS-SCDC_1/2024_OROV-2024	IRCCS Sacro Cuore Don Calabria (SCDC) Hospital in Italy	N/A

**Biological samples**		

Human adult liver organoids	Erasmus Medical Center	N/A
Human fetal liver organoids	Erasmus Medical Center	N/A
Vero cell line	Historically maintained at Laboratory of Gastroenterology and Hepatology, Erasmus Medical Center; also available at ATCC	CRL-1586

**Chemicals, peptides, and recombinant proteins**		

Matrigel Matrix	Corning	Cat#356231
Molnupiravir (EIDD-2801)	MedChemExpress	Cat#HY-135853
EIDD-1931 (Synonyms: β-D-N4-hydroxycytidine; NHC)	MedChemExpress	Cat#HY-125033
Interferon alpha (IFN-α) 2B human	Sigma-Aldrich	Cat#H6166
Filibuvir	MedChemExpress	Cat#HY-10118
Beclabuvir	MedChemExpress	Cat#HY-12429
Bemnifosbuvir	MedChemExpress	Cat#HY-137958A
Tegobuvir	MedChemExpress	Cat#HY-10544
Remdesivir	MedChemExpress	Cat#HY-104077
Ribavirin	Sigma-Aldrich	Cat#R9644
Pimodivir	MedChemExpress	Cat#HY-12353A
T-705 (Favipiravir)	BioVision	Cat#2778-5
Sofosbuvir	MedChemExpress	Cat#HY-15005
Dasabuvir	MedChemExpress	Cat#HY-13998

**Critical commercial assays**		

NucleoSpin RNA Kit	Macherey-Nagel	Cat#740955.250
PrimeScript™ RT Master Mix	Takara Bio	Cat#RR036A
SYBR Green PCR Master Mix	Thermo Fisher Scientific	A46109
CytoTox 96® Non-Radioactive Cytotoxicity Assay	Promega	Cat#G1780
AlamarBlue reagent	Invitrogen	Cat#DAL1100

**Deposited data**		

RNA-seq raw and processed data	This paper	GEO: GSE316780

**Oligonucleotides**		

OROV S segment for RT-qPCR: forward GACAAGTSCTCAATGCTGGTGT, reverse CGTTGTCCGGSACTGGATT	This paper	In this study
Human ALB for RT-qPCR: forward CTGCCTGCCTGTTGCCAAAGC, reverse GGCAAGGTCCGCCCTGTCATC	This paper	In this study
Human CYP3A4 for RT-qPCR: forward AGCAAAGAGCAACACAGAGCTGAA, reverse CAGAGGTGTGGGCCCTGGAAT	This paper	In this study
Human LGR5 for RT-qPCR: forward GTCAGCTGCTCCCGAATCCC, reverse TGAAACAGCTTGGGGGCACA	This paper	In this study
Human MX1 for RT-qPCR: forward GGCTGTTTACCAGACTCCGACA, reverse CACAAAGCCTGGCAGCTCTCTA	This paper	In this study
Human IFIT1 for RT-qPCR: forward GCCTTGCTGAAGTGTGGAGGAA, reverse ATCCAGGCGATAGGCAGAGATC	This paper	In this study
Human ISG15 for RT-qPCR: forward CTCTGAGCATCCTGGTGAGGAA, reverse AAGGTCAGCCAGAACAGGTCGT	This paper	In this study
Human IFI6 for RT-qPCR: forward TGATGAGCTGGTCTGCGATCCT, reverse GTAGCCCATCAGGGCACCAATA	This paper	In this study
Human IFITM3 for RT-qPCR: forward GGTCTTCGCTGGACACCAT, reverse TGTCCCTAGACTTCACGGAGTA	This paper	In this study
Human IFI44L for RT-qPCR: forward TGCACTGAGGCAGATGCTGCG, reverse TCATTGCGGCACACCAGTACAG	This paper	In this study

**Software and algorithms**		

GraphPad Prism8 statistics software	GraphPad Software	https://www.graphpad.com/
MEGA 11	MEGA Software	https://www.megasoftware.net/
AlphaFold3	AlphaFold Server	https://alphafoldserver.com/
CB-Dock2	Liu et al.^50^	https://cadd.labshare.cn/cb-dock2/
AutoDock Vina	Dr. Oleg Trott in the Molecular Graphics Lab^68^^,^^69^	https://vina.scripps.edu/
BIOVIA Discovery Studio Visualizer (v24.1.0.23298)	Dassault Systèmes	https://www.3ds.com/products/biovia/discovery-studio/structure-based-design/

### Experimental models and study participant details

#### Infection diagnosis and liver function tests in human participants

Liver enzyme data (ALT, AST, GGT) were collected from six imported Oropouche cases in Europe for a pilot analysis (Table S1). Next, serum samples were collected from 42 patients with confirmed OROV infection in Ceará State, Brazil, between July and August 2024 (Table S2). Patients were matched by age, sex, and the time interval between symptom onset and sample collection. All cases were confirmed by real-time RT-qPCR targeting OROV with a limit of detection of 2–20 copies, corresponding to Ct values of 34.8–38.1.^70^ The positivity was confirmed in all follow-up clinical samples. All the patients showed OROV-specific antibody seroconversion confirmed by seroneutralization test. Serum levels of liver transaminases (i.e., ALT, AST, GGT, ALP, and bilirubin) were quantified using commercial colorimetric kits from (Wiener Laboratories, Argentina; Cat numbers: 867031000, 861267524, 870580000, 870600000, 867046100, 867013010). Reference ranges for liver function tests were provided by local hospitals or the kit manufacturers: ALT ≤41 U/L for males and ≤31 U/L for females, AST ≤38 U/L for males and ≤32 U/L for females, γ-GT 11–50 U/L for males and 7–32 U/L for females, and for both sexes: ALP ≤240 U/L, total bilirubin ≤1.2 mg/dL, direct bilirubin ≤0.3 mg/dL and indirect bilirubin ≤0.8 mg/dL. All serum samples were stored at −80°C prior to analysis, following manufacturer instructions. Basic clinical and demographic data of the patients are presented in Table S2. Sex as a biological variable. Sex (female/male) was obtained from clinical records; gender was not assessed. Liver function parameters were compared between females and males (Figures 1 and S1). These sex-stratified analyses are exploratory.

#### Culture of human liver-derived organoids

Human fetal (*n* = 1 donor) and adult (*n* = 4 donors, 2 females and 2 males; sex was not specifically analyzed) human liver-derived organoids (ICOs) were isolated by collagenase digestion from tissue samples (≤0.5 cm^3^) of donor liver biopsies.^35^^,^^54^ ICOs were embedded in Matrigel (Corning, USA) and cultured in organoid expansion medium (EM), consisting of advanced DMEM/F12 (Invitrogen, USA), supplemented with 1% penicillin/streptomycin (Life Technologies, USA), 1 M HEPES (Life Technologies, USA), 200 mM ultraglutamine (Life Technologies, USA), 1% (v/v) of N2 (Gibco, USA), 2% (v/v) of B27 (Gibco, USA), 1 mM N-acetylcysteine (Sigma-Aldrich, USA), 10 mM nicotinamide (Sigma-Aldrich, USA), 5 μM A83.01 (Tocris Bioscience, UK), 10 μM forskolin (Tocris Bioscience, UK), 10 nM gastrin (Sigma-Aldrich, USA), epidermal growth factor (EGF) (50 ng/mL; PeproTech, USA), 10% (v/v) of R-spondin-1 (conditioned medium), fibroblast growth factor 10 (FGF10) (100 ng/mL; PeproTech, USA), hepatocyte growth factor (HGF) (25 ng/mL; PeproTech, USA), and 10 μM Y27632 (Sigma-Aldrich, USA). To ensure the consistent quality and the reproducibility, only early-passage organoids (up to 12 passages) were used in this study. All adult liver organoids used in this study were derived from donor livers originally obtained for transplantation. Use of liver tissues for research purposes was approved by the Erasmus MC Medical Ethical Council, and informed consent was given (MEC2006-202 for fetal liver tissues and MEC-2014-060 for adult liver tissues).

#### Cell culture

Vero cells were maintained in Dulbecco’s modified Eagle medium (DMEM; Lonza) supplemented with 10% fetal calf serum (Hyclone, Logan, USA) and 100 U/mL penicillin-streptomycin, and cultured in a humidified incubator at 37°C and 5% CO_2_. All cell cultures were confirmed to be mycoplasma negative through regularly testing by GATC Biotech (Konstanz, Germany). The laboratory implemented a policy of authentication of all cell lines using the short tandem repeat genotyping assay performed at the Department of Pathology, Erasmus Medical Center Rotterdam. However, this assay is specific for human species, while Vero cells are of monkey origin, and therefore genotyping was not performed.

### Method details

#### Immunohistochemistry

Organoids were formalin-fixed (4%) for 24 h, paraffin-embedded and sectioned (4 μm) for histological examination. To assess overall morphology, sections were stained with hematoxylin and eosin (H&E) and imaged by bright-field microscopy. Additional sections were processed for immunohistochemistry (IHC) using a primary antibody against cytokeratin 19 (KRT19; clone RCK108, Agilent Dako). After deparaffinization and antigen retrieval in citrate buffer (pH 6.0) at boiling temperature for 10 min, sections were incubated with the primary antibody overnight at 4°C, followed by 1 h incubation with secondary antibody (Envision Flex HRP, Agilent Dako) at room temperature, and finally developed using DAB substrate. Slides were imaged using a standard bright-field microscope.

#### Hepatocyte differentiation of organoids

ICOs cultured from adult liver were expanded for 5–7 days before initiation of hepatocyte differentiation. Differentiation toward hepatocyte fate was initiated with the addition of 25 ng/mL BMP7 (25 ng/mL; PeproTech) to the EM and lasted 5 days. Subsequently, organoids were passaged with a 1:1.5 split ratio and medium was changed to human hepatocyte differentiation medium (DM): AdDMEM/F12 medium supplemented with 1% penicillin/streptomycin (Life Technologies), 1 M HEPES (Life Technologies), 200 mM ultraglutamine (Life Technologies), 1% N2 and 1% B27 (with vitamin A) and containing EGF (50 ng/mL; PeproTech), 10 nM gastrin (Sigma-Aldrich), HGF (25 ng/mL; PeproTech), FGF19 (100 ng/mL; PeproTech), 500 nM A8301 (Tocris), 10 μM DAPT (Sigma-Aldrich), BMP7 (25 ng/mL; PeproTech), and 30 μM dexamethasone (Sigma-Aldrich). DM was refreshed every 2 to 3 days for a period of 12 days^35^^,^^54^

#### OROV strains and viral inoculation

The historical OROV strain TRVL9760 (OROV-1967) was propagated through nine successive passages in mice by intracerebral inoculation. Subsequently, virus stocks were produced by infecting Vero cells with homogenized brain tissue from the infected mice. This strain was obtained by the National Institute for Public Health and the Environment, The Netherlands. The OROV strain IRCCS-SCDC_1/2024_OROV-2024 (OROV-2024) was isolated from an imported case in Italy.^30^ Human ICOs, fetal liver-derived organoids, and hepatocyte-differentiated organoids were all infected using the same standardized protocol. Organoids were mechanically fragmented and then exposed to OROV at a dose of 10^6^ PFU per ∼10,000 organoids for 2 h at 37°C. To enhance the infection efficiency, the virus-organoid suspension was gently resuspended every 30 min during the incubation. Subsequently, fragmented organoids were centrifuged at 300 g for 5 min at 4°C, and the supernatant was discarded. Organoids were then washed three times with advanced DMEM/F12 to remove residual viruses, embedded in Matrigel, and cultured in EM. Each experimental group included at least four biological replicates, defined as independent organoid cultures.

#### Antiviral compound treatment and IC_50_/CC_50_ analysis

To evaluate antiviral efficacy, OROV-infected human liver organoids were treated with single-dose or serial dilutions of each compound. Organoids were first inoculated with OROV for 2 h, then were washed, re-embedded in Matrigel, and transferred into 48-well plates containing expansion medium supplemented with the respective compounds. Treated organoids were then incubated for 48, 72, or 96 h depending on the experimental design. After treatment, infected organoids and supernatants were collected for following analyses. For IC_50_ and CC_50_ analysis, relatively OROV RNA levels in organoids were quantified by RT-qPCR after 48h treatment, and cell viability was assessed by AlamarBlue assay in parallel. Dose-response curves were fitted using nonlinear regression, and IC_50_ and CC_50_ values were calculated accordingly.^52^

#### Phylogenic analysis

We selected 1–2 representative strains from each cluster to construct a concise phylogenetic tree that clearly distinguishes between the clusters.^71^^,^^72^ Specifically, the complete genomes (L, M and S segment) of OROV-2024, OROV-1967, and six representative OROV strains (strain ID: BeAn19991, BeH 390242, BeAn 626990, Esmeraldas/206, FPM01278, LACENAM_ILMD_0461SLS), along with one Perdões virus (strain ID: BeAn789726), one Madre de Dios virus strain (strain ID: FMD 1303), and one Iquitos virus strain (strain ID: MIS-0397) were included in the phylogenetic analysis. The Jatobal virus (strain ID: BeAn 423380) was used as the outgroup. Sequences were aligned using the MAFFT 7.^73^ The phylogenetic tree was reconstructed using the neighbor-joining (NJ) method with branch support evaluated using the Bootstrap method (1000 replicates) in MEGA 11.^74^

The evolutionary distances were computed using the p-distance method and are in the units of the number of base differences per site. The rate variation among sites was modeled with a gamma distribution (shape parameter = 4). The resulting phylogenetic tree was visualized and edited using MEGA 11, with bootstrap values greater than 70% shown below the branches to indicate significant support. The tree was rooted using the midpoint method, and branch lengths were scaled to represent evolutionary distances. To enhance interpretability, visual adjustments were made, including color-coding the strains from our study.

#### Molecular docking

We conducted molecular docking experiments following the workflow illustrated in Figure S5A. Briefly, AlphaFold3 was used to predict the RdRp protein structures of OROV-1967 and OROV-2024, with a predicted template modeling (pTM) score above 0.8, indicating high-quality structural predictions.^48^ Next, the CurPocket tool within the CB-Dock2v,^50^ which uses a web-based protein surface curvature cavity detection method, was employed to identify potential cavities on the predicted OROV RdRp proteins. Amino acid sites on the OROV RdRp were annotated based on previous research,^36^ and the cavity affecting RNA-protein contact was selected as docking configurations. Subsequently, docking experiments between molnupiravir, NHC, NHC-5′-triphosphate and the OROV RdRp models were conducted using CB-Dock2, a protein-ligand blind docking web server powered by AutoDock Vina (version 1.1.2),^68^^,^^69^ to verify the accuracy of the pocket detection process.^50^ The 2D schematic diagram illustrating the intermolecular interactions between receptor and ligand molecules was generated using BIOVIA Discovery Studio Visualizer (v24.1.0.23298).

#### RNA extraction and RT-qPCR detection

Total RNA was extracted using the NucleoSpin RNA Kit (MACHEREY-NAGEL, Germany) and quantified by Nanodrop ND-1000 (Wilmington, USA). cDNA was synthesized using a cDNA synthesis kit (Takara Bio, Japan). Viral DNA levels were quantified by SYBR Green-based RT-qPCR (Applied Biosystems SYBR Green PCR Master Mix; Thermo Fisher Scientific) with the QuantStudio Real-Time PCR Systems (Applied Biosystems by Thermo Fisher Scientific). Relative gene expression was normalized to GAPDH using the formula 2^−ΔΔCT^ (ΔΔCT = ΔCTsample − ΔCTcontrol). Primers used for quantitative reverse transcription PCR (qRT-PCR) were synthesized by Sigma-Aldrich (USA).

#### Quantification of virus genome copy numbers

To determine viral genome copy numbers, cDNA from OROV infected organoids was used as template to generate the insert DNA, which was then cloned into the vector to generate the plasmid containing OROV S segment sequence. The plasmid was serially diluted 10-fold from 10^2^ to 10^8^ copies and quantified by RT-qPCR to establish a standard curve. The linear regression equation was determined as Ct = −3.323 × log_10_[copies] + 35.47, with an R^2^ of 0.9989. Based on the standard deviation of the residuals (Sy.x = 0.2406), the limit of detection (LOD) and limit of quantification (LOQ) were calculated as 1.65 copies and 5.31 copies per reaction, respectively. These correspond to Ct values of approximately 34.9 (LOD) and 33.1 (LOQ).

#### TCID_50_ assay

Infectious OROV titers were determined using a 50% tissue culture infectious dose (TCID50) assay. Briefly, serial 10-fold dilutions of harvested supernatant were inoculated onto monolayers of Vero cells seeded at a density of 2,000 cells per well in 96-well plates (*n* = 8 wells per dilution). Plates were incubated at 37°C for 3–4 days and monitored daily for CPE using light microscopy. CPE was scored as present or absent in each well based on the changes of cell morphology, including cell rounding, detachment, and syncytia formation.^75^ The TCID_50_ value was calculated by using the Reed-Muench method.^76^

#### Immunofluorescence assay

Organoids were fixed in 4% paraformaldehyde solution at 4°C overnight. The slides containing organoids were then rinsed three times with phosphate-buffered saline (PBS) for 5 min each time, followed by permeabilizing with PBS containing 0.2% (v/v) Triton X-100 for 5 min. Then, the slides were twice rinsed with PBS for 5 min, followed by incubation with blocking solution (5% donkey serum, 1% bovine serum albumin, and 0.2% Triton X-100 in PBS) at room temperature for 1 h. Next, the slides were incubated in a humidity chamber with primary antibody diluted in blocking solution at 4°C overnight. Primary antibodies used in this study are as follows: Anti-Oropouche orthobunyavirus Gc (1:100, mouse monoclonal antibody; clone 2B5B1; provided by Dr. Sven Reiche from Federal Research Institute for Animal Health, Germany) and Human Active Caspase-3 Antibody (R&D Systems, Bio-Techne, USA). Slides were washed three times for 5 min each in PBS before 1-h incubation with 1:1000 dilutions of the anti-mouse immunoglobulin G (IgG) (H + L, Alexa Fluor 594, Invitrogen, USA) secondary antibodies and 20-min incubation with 1:200 Phalloidin (Alexa Fluor 488, Invitrogen, USA). Nuclei were stained with DAPI (VECTASHIELD Vector Laboratories, USA). Images were obtained using Leica SP5 cell imaging system.

#### LDH release assay

Organoid culture supernatants were collected at the indicated time points, and LDH release was quantified using the CytoTox 96 Non-Radioactive Cytotoxicity Assay (Promega, USA) following the manufacturer’s instructions. Briefly, 50 μL of each sample was transferred to a 96-well plate and incubated with 50 μL of reconstituted substrate mix at room temperature for 30 min in the dark. The reaction was stopped by adding 50 μL of stop solution, and absorbance was measured at 490 nm using a microplate reader (Thermo Fisher Scientific, USA).

#### AlamarBlue assay

Culture supernatant was discarded, and the organoids were incubated with a 1:20 dilution of AlamarBlue reagent (Invitrogen, USA) in culture medium for 2 h at 37°C. Subsequently, 100 μL of the medium was collected to assess cell metabolic activity, with each sample being measured in triplicate. Absorbance measurements were obtained using a CytoFluor Series 4000 plate reader (Thermo Fisher Scientific, USA) at an excitation wavelength of 530/25 nm and an emission wavelength of 590/35 nm.

#### Genome-wide RNA sequencing and data analysis

Organoids cultured from adult human liver tissue were infected with both OROV isolates as described above. Infected organoids were harvested at 1- 48-, and 96-h post-infection. A treatment group was established using organoids infected with the OROV-2024 isolate and treated with 1 μM NHC treatment from 1 h to 96 h post-inoculation. As negative controls, uninfected organoids were cultured under same conditions for 96 h. Total RNA was extracted from all samples using the MachereyNagel NucleoSpin RNA II Kit (Bioke, Netherlands) and quantified with the Bioanalyzer RNA 6000 Picochip. Briefly, mRNA was purified from total RNA using poly-T oligo-attached magnetic beads for cDNA synthesis and library construction by Novogene. The library was checked with Qubit and real-time PCR for quantification and bioanalyzer for size distribution detection. RNA sequencing was conducted using a paired-end 150 bp (PE 150) sequencing strategy via Illumina platforms.^77^ RNA sequencing datasets have been deposited to NCBI GEO: GSE316780 at https://www.ncbi.nlm.nih.gov/geo/query/acc.cgi?acc=GSE316780.

Novogene provided a standard bioinformatic analysis for the host (human) mRNA sequencing data of with a well-annotated reference genome. Gene expression levels were quantified as fragments per kilobase of transcript per million mapped reads (FPKM). The coexpression Venn diagram showed the number of genes uniquely or commonly expressed across groups. Differential gene expression analysis was performed for comparison between two groups,^78^ and the results were visualized using volcano plots. Functional annotation was conducted using the clusterProfiler package for gene ontology (GO) enrichment analysis,^79^ and gene set enrichment analysis (GSEA) was used to evaluate pathway-level transcriptional changes. For heatmap visualization, gene expression values were row-wise *Z* score normalized across different groups, and we used the same uninfected group as a background reference to ensure comparability across conditions.

#### Passaging OROV under NHC pressure and nanopore sequencing

Vero cells were seeded in 6-well plates on the day prior to inoculation with OROV. Independent triplicate cultures were infected with OROV and in the presence of NHC and triplicates with culture medium only served as the control. OROV was inoculated into each well at 10^5^ TCID_50_ for the initial culture at the final volume of 2 mL per well, and NHC was added starting at 0.01 μM. Every two days, infected cultures were harvested by freeze-thawing the cells together with the supernatant. 100 μL of the collected virus-containing medium was then used to infect fresh Vero cells, and the NHC concentration was increased at each passage. Passaging continued until the drug concentration reached 9 μM, after which samples from the final passage was collected for Nanopore sequencing.

Briefly, whole genome sequencing of the OROV stock and cultured isolates was performed using amplicon-based sequencing as previously described on the Nanopore platform.^80^ Consensus sequence analysis was performed using a custom build Nanopore analysis pipeline (https://github.com/EMC-Viroscience/nanopore-amplicon-analysis-manual). Minor variant analysis was performed using Genenious Prime version 2026.0.1 using a minimum of 30x coverage and a minimum of 20% variant frequency.

### Quantification and statistical analysis

Statistical analysis was performed using GraphPad Prism8 statistics software (GraphPad, USA). Patient data are presented as values measured from individual patients. Experimental data are presented as mean ± standard error of the mean (SEM), based on biological replicates from at least 2 independent experiments, unless specified. All statistical details of experiments can be found in the figure legends. Comparison between two groups was analyzed by Mann-Whitney U test. For longitudinal data, a mixed-effects model (restricted maximum likelihood, REML) was used to assess the effects of time, treatment, and their interaction. When applicable, Dunnett’s multiple comparisons test was used for post hoc analysis to compare treatment groups against the control. Asterisks indicated the degree of significant differences compared with the controls (∗*p* < 0.05; ∗∗*p* < 0.01; ∗∗∗*p* < 0.001; ∗∗∗∗*p* < 0.0001).

## Supplemental information

Document S1. Figures S1–S10 and Tables S1–S3

Document S2. Article plus supplemental information

## References

[1] Anderson C.R., Spence L., Downs W.G., Aitken T.H. (1961). Oropouche virus: a new human disease agent from Trinidad, West Indies. Am. J. Trop. Med. Hyg..

[2] Scachetti G.C., Forato J., Claro I.M., Hua X., Salgado B.B., Vieira A., Simeoni C.L., Barbosa A.R.C., Rosa I.L., de Souza G.F. (2024). Re-emergence of Oropouche virus between 2023 and 2024 in Brazil: an observational epidemiological study. Lancet Infect. Dis..

[3] Moreira F.R.R., Dutra J.V.R., de Carvalho A.H.B., Reis C.R., Rios J.S.H., Ribeiro M.d.O., Arruda M.B., Alvarez P., Souza R.P., Voloch C. (2024). Oropouche virus genomic surveillance in Brazil. Lancet Infect. Dis..

[4] Naveca F.G., Almeida T.A.P.d., Souza V., Nascimento V., Silva D., Nascimento F., Mejía M., Oliveira Y.S.d., Rocha L., Xavier N. (2024). Human outbreaks of a novel reassortant Oropouche virus in the Brazilian Amazon region. Nat. Med..

[5] Moutinho S. (2024). Little-known virus is on the rise in South America. Science.

[6] Quaglia S. (2024). Clues emerge about an obscure virus' sudden spread. Science.

[7] Morrison A., White J.L., Hughes H.R., Guagliardo S.A.J., Velez J.O., Fitzpatrick K.A., Davis E.H., Stanek D., Kopp E., Dumoulin P. (2024). Oropouche Virus Disease Among U.S. Travelers - United States, 2024. MMWR Morb. Mortal. Wkly. Rep..

[8] Castilletti C., Huits R., Mantovani R.P., Accordini S., Alladio F., Gobbi F. (2024). Replication-Competent Oropouche Virus in Semen of Traveler Returning to Italy from Cuba, 2024. Emerg. Infect. Dis..

[9] Igloi Z., Soochit W., Munnink B.B.O., Anas A.A., von Eije K.J., van der Linden A., Mandigers M., Wijnans K., Voermans J., Chandler F.D. (2025). Oropouche Virus Genome in Semen and Other Body Fluids from Traveler. Emerg. Infect. Dis..

[10] Bandeira A.C., Pereira F.M., Leal A., Santos S.P.O., Barbosa A.C., Souza M.S.P.L., de Souza D.R., Guimaraes N., Fonseca V., Giovanetti M. (2024). Fatal Oropouche Virus Infections in Nonendemic Region, Brazil, 2024. Emerg. Infect. Dis..

[11] Vernal S., Martini C.C.R., da Fonseca B.A.L. (2019). Oropouche Virus-Associated Aseptic Meningoencephalitis, Southeastern Brazil. Emerg. Infect. Dis..

[12] Wesselmann K.M., Postigo-Hidalgo I., Pezzi L., de Oliveira-Filho E.F., Fischer C., de Lamballerie X., Drexler J.F. (2024). Emergence of Oropouche fever in Latin America: a narrative review. Lancet Infect. Dis..

[13] Martins-Filho P.R., Carvalho T.A., Dos Santos C.A. (2024). Oropouche fever: reports of vertical transmission and deaths in Brazil. Lancet Infect. Dis..

[14] das Neves Martins F.E., Chiang J.O., Nunes B.T.D., Ribeiro B.F.R., Martins L.C., Casseb L.M.N., Henriques D.F., de Oliveira C.S., Maciel E.L.N., Azevedo R.D.S. (2024). Newborns with microcephaly in Brazil and potential vertical transmission of Oropouche virus: a case series. Lancet Infect. Dis..

[15] de Souza W.M., Calisher C.H., Carrera J.P., Hughes H.R., Nunes M.R.T., Russell B., Tilson-Lunel N.L., Venter M., Xia H. (2024). ICTV Virus Taxonomy Profile: Peribunyaviridae 2024. J. Gen. Virol..

[16] Travassos da Rosa J.F., de Souza W.M., Pinheiro F.d.P., Figueiredo M.L., Cardoso J.F., Acrani G.O., Nunes M.R.T. (2017). Oropouche Virus: Clinical, Epidemiological, and Molecular Aspects of a Neglected Orthobunyavirus. Am. J. Trop. Med. Hyg..

[17] Pinheiro F.P., Travassos da Rosa A.P., Gomes M.L., LeDuc J.W., Hoch A.L. (1982). Transmission of Oropouche virus from man to hamster by the midge Culicoides paraensis. Science.

[18] Benitez A.J., Alvarez M., Perez L., Gravier R., Serrano S., Hernandez D.M., Perez M.M., Gutierrez-Bugallo G., Martinez Y., Companioni A. (2024). Oropouche Fever, Cuba, May 2024. Emerg. Infect. Dis..

[19] Peinado R.D.S., Eberle R.J., Arni R.K., Coronado M.A. (2022). A Review of Omics Studies on Arboviruses: Alphavirus, Orthobunyavirus and Phlebovirus. Viruses.

[20] Geddes V.E.V., de Oliveira A.S., Tanuri A., Arruda E., Ribeiro-Alves M., Aguiar R.S. (2018). MicroRNA and cellular targets profiling reveal miR-217 and miR-576-3p as proviral factors during Oropouche infection. PLoS Negl. Trop. Dis..

[21] Rodrigues A.H., Santos R.I., Arisi G.M., Bernardes E.S., Silva M.L., Rossi M.A., Lopes M.B.S., Arruda E. (2011). Oropouche virus experimental infection in the golden hamster (Mesocrisetus auratus). Virus Res..

[22] Araujo R., Dias L.B., Araujo M.T., Pinheiro F., Oliva O.F. (1978). [Ultrastructural changes in the hamster liver after experimental inoculation with Oropouche arbovirus (type BeAn 19991)] Alteracoes ultraestruturais no figado de hamster apos inoculacao experimental com arbovirus Oropouche (tipo BeAn 19991). Rev. Inst. Med. Trop. Sao Paulo.

[23] Proenca-Modena J.L., Sesti-Costa R., Pinto A.K., Richner J.M., Lazear H.M., Lucas T., Hyde J.L., Diamond M.S. (2015). Oropouche virus infection and pathogenesis are restricted by MAVS, IRF-3, IRF-7, and type I interferon signaling pathways in nonmyeloid cells. J. Virol..

[24] Proenca-Modena J.L., Hyde J.L., Sesti-Costa R., Lucas T., Pinto A.K., Richner J.M., Gorman M.J., Lazear H.M., Diamond M.S. (2016). Interferon-Regulatory Factor 5-Dependent Signaling Restricts Orthobunyavirus Dissemination to the Central Nervous System. J. Virol..

[25] da Silva Menegatto M.B., Ferraz A.C., Lima R.L.S., Almeida L.T., de Brito R.C.F., Reis A.B., Carneiro C.M., de Lima W.G., de Mello Silva B., de Magalhães J.C., Magalhães C.L.d.B. (2023). Oropouche virus infection induces ROS production and oxidative stress in liver and spleen of mice. J. Gen. Virol..

[26] da Costa V.G., de Rezende Féres V.C., Saivish M.V., de Lima Gimaque J.B., Moreli M.L. (2017). Silent emergence of Mayaro and Oropouche viruses in humans in Central Brazil. Int. J. Infect. Dis..

[27] Alva-Urcia C., Aguilar-Luis M.A., Palomares-Reyes C., Silva-Caso W., Suarez-Ognio L., Weilg P., Manrique C., Vasquez-Achaya F., Del Valle L.J., Del Valle-Mendoza J. (2017). Emerging and reemerging arboviruses: A new threat in Eastern Peru. PLoS One.

[28] Co A.C.G., de Mendonca G.C., Gatti F.D., Sousa T.J., Tavares E.A., Nodari J.Z., de Moura R.G., Lopes P.O., Pereira J.D.P., Alves L.N.R. (2025). Unravelling the pathogenesis of Oropouche virus. Lancet Infect. Dis..

[29] Barbiero A., Formica G., Mantovani R.P., Accordini S., Gobbi F., Spinicci M., Colao M.G., Pollini S., Ciccone N., Rossolini G.M. (2024). Persistent Oropouche virus viremia in two travellers returned to Italy from Cuba, July 2024. J Travel Med.

[30] Deiana M., Malago S., Mori A., Accordini S., Matucci A., Passarelli Mantovani R., Gianesini N., Huits R., Piubelli C., Gobbi F.G. (2024). Full Genome Characterization of the First Oropouche Virus Isolate Imported in Europe from Cuba. Viruses.

[31] van Beek J.H.D.A., de Moor M.H.M., de Geus E.J.C., Lubke G.H., Vink J.M., Willemsen G., Boomsma D.I. (2013). The genetic architecture of liver enzyme levels: GGT, ALT and AST. Behav. Genet..

[32] Lefebvre P., Staels B. (2021). Hepatic sexual dimorphism - implications for non-alcoholic fatty liver disease. Nat. Rev. Endocrinol..

[33] Tamber S.S., Bansal P., Sharma S., Singh R.B., Sharma R. (2023). Biomarkers of liver diseases. Mol. Biol. Rep..

[34] Marsee A., Roos F.J.M., Verstegen M.M.A., Gehart H., de Koning E., Lemaigre F., Forbes S.J., Peng W.C., Huch M., Takebe T. (2021). Building consensus on definition and nomenclature of hepatic, pancreatic, and biliary organoids. Cell Stem Cell.

[35] Huch M., Gehart H., van Boxtel R., Hamer K., Blokzijl F., Verstegen M.M.A., Ellis E., van Wenum M., Fuchs S.A., de Ligt J. (2015). Long-term culture of genome-stable bipotent stem cells from adult human liver. Cell.

[36] Arragain B., Effantin G., Gerlach P., Reguera J., Schoehn G., Cusack S., Malet H. (2020). Pre-initiation and elongation structures of full-length La Crosse virus polymerase reveal functionally important conformational changes. Nat. Commun..

[37] Santos R.I.M., Rodrigues A.H., Silva M.L., Mortara R.A., Rossi M.A., Jamur M.C., Oliver C., Arruda E. (2008). Oropouche virus entry into HeLa cells involves clathrin and requires endosomal acidification. Virus Res..

[38] de Almeida A.L.T., da Costa I.P.S., Garcia M.D.d.N., da Silva M.A.N., Lazzaro Y.G., de Filippis A.M.B., Nogueira F.d.B., Barreto-Vieira D.F. (2025). Oropouche Virus: Isolation and Ultrastructural Characterization from a Human Case Sample from Rio de Janeiro, Brazil, Using an In Vitro System. Viruses.

[39] Concha J.O., Gutierrez K., Barbosa N., Rodrigues R.L., de Carvalho A.N., Tavares L.A., Rudd J.S., Costa C.S., Andrade B.Y.G., Espreafico E.M. (2024). Rab27a GTPase and its effector Myosin Va are host factors required for efficient Oropouche virus cell egress. PLoS Pathog..

[40] Schoggins J.W., Wilson S.J., Panis M., Murphy M.Y., Jones C.T., Bieniasz P., Rice C.M. (2011). A diverse range of gene products are effectors of the type I interferon antiviral response. Nature.

[41] Ahrens S., Zelenay S., Sancho D., Hanč P., Kjær S., Feest C., Fletcher G., Durkin C., Postigo A., Skehel M. (2012). F-actin is an evolutionarily conserved damage-associated molecular pattern recognized by DNGR-1, a receptor for dead cells. Immunity.

[42] Suarez-Huerta N., Mosselmans R., Dumont J.E., Robaye B. (2000). Actin depolymerization and polymerization are required during apoptosis in endothelial cells. J. Cell. Physiol..

[43] Bursch W., Hochegger K., Torok L., Marian B., Ellinger A., Hermann R.S. (2000). Autophagic and apoptotic types of programmed cell death exhibit different fates of cytoskeletal filaments. J. Cell Sci..

[44] Tian L., Qiang T., Liang C., Ren X., Jia M., Zhang J., Li J., Wan M., YuWen X., Li H. (2021). RNA-dependent RNA polymerase (RdRp) inhibitors: The current landscape and repurposing for the COVID-19 pandemic. Eur. J. Med. Chem..

[45] Zibat A., Zhang X., Dickmanns A., Stegmann K.M., Dobbelstein A.W., Alachram H., Soliwoda R., Salinas G., Groß U., Görlich D. (2023). N4-hydroxycytidine, the active compound of Molnupiravir, promotes SARS-CoV-2 mutagenesis and escape from a neutralizing nanobody. iScience.

[46] Strizki J.M., Gaspar J.M., Howe J.A., Hutchins B., Mohri H., Nair M.S., Kinek K.C., McKenna P., Goh S.L., Murgolo N. (2024). Molnupiravir maintains antiviral activity against SARS-CoV-2 variants and exhibits a high barrier to the development of resistance. Antimicrob. Agents Chemother..

[47] Maas B.M., Strizki J., Miller R.R., Kumar S., Brown M., Johnson M.G., Cheng M., De Anda C., Rizk M.L., Stone J.A. (2024). Molnupiravir: Mechanism of action, clinical, and translational science. Clin. Transl. Sci..

[48] Abramson J., Adler J., Dunger J., Evans R., Green T., Pritzel A., Ronneberger O., Willmore L., Ballard A.J., Bambrick J. (2024). Accurate structure prediction of biomolecular interactions with AlphaFold 3. Nature.

[49] Toots M., Yoon J.J., Cox R.M., Hart M., Sticher Z.M., Makhsous N., Plesker R., Barrena A.H., Reddy P.G., Mitchell D.G. (2019). Characterization of orally efficacious influenza drug with high resistance barrier in ferrets and human airway epithelia. Sci. Transl. Med..

[50] Liu Y., Yang X., Gan J., Chen S., Xiao Z.X., Cao Y. (2022). CB-Dock2: improved protein-ligand blind docking by integrating cavity detection, docking and homologous template fitting. Nucleic Acids Res..

[51] Kabinger F., Stiller C., Schmitzová J., Dienemann C., Kokic G., Hillen H.S., Höbartner C., Cramer P. (2021). Mechanism of molnupiravir-induced SARS-CoV-2 mutagenesis. Nat. Struct. Mol. Biol..

[52] Wang Y., Li P., Solanki K., Li Y., Ma Z., Peppelenbosch M.P., Baig M.S., Pan Q. (2021). Viral polymerase binding and broad-spectrum antiviral activity of molnupiravir against human seasonal coronaviruses. Virology.

[53] Garcia Filho C., Lima Neto A.S., Maia A.M.P.C., da Silva L.O.R., Cavalcante R.D.C., Monteiro H.D.S., Marques K.C.A., Oliveira R.d.S., Gadelha S.d.A.C., Nunes de Melo D. (2024). A Case of Vertical Transmission of Oropouche Virus in Brazil. N. Engl. J. Med..

[54] Li P., Li Y., Wang Y., Liu J., Lavrijsen M., Li Y., Zhang R., Verstegen M.M.A., Wang Y., Li T.C. (2022). Recapitulating hepatitis E virus-host interactions and facilitating antiviral drug discovery in human liver-derived organoids. Sci. Adv..

[55] Sanderson T., Hisner R., Donovan-Banfield I., Hartman H., Løchen A., Peacock T.P., Ruis C. (2023). A molnupiravir-associated mutational signature in global SARS-CoV-2 genomes. Nature.

[56] Hoofnagle J.H., Seeff L.B. (2006). Peginterferon and ribavirin for chronic hepatitis C. N. Engl. J. Med..

[57] Zheng S., Wang W., Aldahdooh J., Malyutina A., Shadbahr T., Tanoli Z., Pessia A., Tang J. (2022). SynergyFinder Plus: Toward Better Interpretation and Annotation of Drug Combination Screening Datasets. Genom. Proteom. Bioinform..

[58] Chinnarasu S., Alogaili F., Bove K.E., Jaeschke A., Hui D.Y. (2021). Hepatic LDL receptor-related protein-1 deficiency alters mitochondrial dynamics through phosphatidylinositol 4,5-bisphosphate reduction. J. Biol. Chem..

[59] Hamlin A.N., Chinnarasu S., Ding Y., Xian X., Herz J., Jaeschke A., Hui D.Y. (2018). Low-density lipoprotein receptor-related protein-1 dysfunction synergizes with dietary cholesterol to accelerate steatohepatitis progression. J. Biol. Chem..

[60] Schwarz M.M., Price D.A., Ganaie S.S., Feng A., Mishra N., Hoehl R.M., Fatma F., Stubbs S.H., Whelan S.P.J., Cui X. (2022). Oropouche orthobunyavirus infection is mediated by the cellular host factor Lrp1. Proc. Natl. Acad. Sci. USA.

[61] Schwarz M.M., Ganaie S.S., Feng A., Brown G., Yangdon T., White J.M., Hoehl R.M., McMillen C.M., Rush R.E., Connors K.A. (2023). Lrp1 is essential for lethal Rift Valley fever hepatic disease in mice. Sci. Adv..

[62] Bermudez-Mendez E., Angelino P., van Keulen L., van de Water S., Rockx B., Pijlman G.P., Ciuffi A., Kortekaas J., Wichgers Schreur P.J. (2023). Transcriptomic Profiling Reveals Intense Host-Pathogen Dispute Compromising Homeostasis during Acute Rift Valley Fever Virus Infection. J. Virol..

[63] Jayk Bernal A., Gomes da Silva M.M., Musungaie D.B., Kovalchuk E., Gonzalez A., Delos Reyes V., Martín-Quirós A., Caraco Y., Williams-Diaz A., Brown M.L. (2022). Molnupiravir for Oral Treatment of Covid-19 in Nonhospitalized Patients. N. Engl. J. Med..

[64] Li P., Wang Y., Lavrijsen M., Lamers M.M., de Vries A.C., Rottier R.J., Bruno M.J., Peppelenbosch M.P., Haagmans B.L., Pan Q. (2022). SARS-CoV-2 Omicron variant is highly sensitive to molnupiravir, nirmatrelvir, and the combination. Cell Res..

[65] Nakamura K., Fujimoto K., Hasegawa C., Aoki I., Yoshitsugu H., Ugai H., Yatsuzuka N., Tanaka Y., Furihata K., Maas B.M. (2022). A phase I, randomized, placebo-controlled study of molnupiravir in healthy Japanese to support special approval in Japan to treat COVID-19. Clin. Transl. Sci..

[66] Livonesi M.C., De Sousa R.L.M., Badra S.J., Figueiredo L.T.M. (2006). In vitro and in vivo studies of ribavirin action on Brazilian Orthobunyavirus. Am. J. Trop. Med. Hyg..

[67] Livonesi M.C., de Sousa R.L.M., Badra S.J., Figueiredo L.T.M. (2007). In vitro and in vivo studies of the Interferon-alpha action on distinct Orthobunyavirus. Antiviral Res..

[68] Eberhardt J., Santos-Martins D., Tillack A.F., Forli S. (2021). AutoDock Vina 1.2.0: New Docking Methods, Expanded Force Field, and Python Bindings. J. Chem. Inf. Model..

[69] Trott O., Olson A.J. (2010). AutoDock Vina: improving the speed and accuracy of docking with a new scoring function, efficient optimization, and multithreading. J. Comput. Chem..

[70] Naveca F.G., Nascimento V.A.D., Souza V.C.d., Nunes B.T.D., Rodrigues D.S.G., Vasconcelos P.F.d.C. (2017). Multiplexed reverse transcription real-time polymerase chain reaction for simultaneous detection of Mayaro, Oropouche, and Oropouche-like viruses. Mem. Inst. Oswaldo Cruz.

[71] Vasconcelos H.B., Nunes M.R.T., Casseb L.M.N., Carvalho V.L., Pinto da Silva E.V., Silva M., Casseb S.M.M., Vasconcelos P.F.C. (2011). Molecular epidemiology of Oropouche virus, Brazil. Emerg. Infect. Dis..

[72] Gutierrez B., Wise E.L., Pullan S.T., Logue C.H., Bowden T.A., Escalera-Zamudio M., Trueba G., Nunes M.R.T., Faria N.R., Pybus O.G. (2020). Evolutionary Dynamics of Oropouche Virus in South America. J. Virol..

[73] Katoh K., Misawa K., Kuma K.i., Miyata T. (2002). MAFFT: a novel method for rapid multiple sequence alignment based on fast Fourier transform. Nucleic Acids Res..

[74] Tamura K., Stecher G., Kumar S. (2021). MEGA11: Molecular Evolutionary Genetics Analysis Version 11. Mol. Biol. Evol..

[75] Berger A., Drosten C., Doerr H.W., Stürmer M., Preiser W. (2004). Severe acute respiratory syndrome (SARS)--paradigm of an emerging viral infection. J. Clin. Virol..

[76] Biacchesi S., Skiadopoulos M.H., Yang L., Murphy B.R., Collins P.L., Buchholz U.J. (2005). Rapid human metapneumovirus microneutralization assay based on green fluorescent protein expression. J. Virol. Methods.

[77] Li P., Pachis S.T., Xu G., Schraauwen R., Incitti R., de Vries A.C., Bruno M.J., Peppelenbosch M.P., Alam I., Raymond K., Pan Q. (2023). Mpox virus infection and drug treatment modelled in human skin organoids. Nat. Microbiol..

[78] Love M.I., Huber W., Anders S. (2014). Moderated estimation of fold change and dispersion for RNA-seq data with DESeq2. Genome Biol..

[79] Yu G., Wang L.G., Han Y., He Q.Y. (2012). clusterProfiler: an R package for comparing biological themes among gene clusters. OMICS.

[80] de Melo Iani F.C., Pereira F.M., de Oliveira E.C., Rodrigues J.T.N., Machado M.H., Fonseca V., Adelino T.E.R., Guimaraes N.R., Tome L.M.R., Gomez M.K.A. (2025). Travel-associated international spread of Oropouche virus beyond the Amazon. J Travel Med.

