# Supplementary material for: Human liver-derived organoids recapitulate Oropouche virus infection and manifestation, enabling antiviral drug discovery
Source: Cell Rep Med. 2026 Mar 9;7(3):102646. doi: 10.1016/j.xcrm.2026.102646 (PMC13006397; doi:10.1016/j.xcrm.2026.102646)
Supplement: Document S1. Figures S1–S10 and Tables S1–S3 [file mmc1.pdf]

Cell Reports Medicine, Volume *7*

Supplemental information

Human liver-derived organoids recapitulate Oropouche virus infection and manifestation, enabling antiviral drug discovery

Jiajing Li, Xin Wang, Yibo Ding, Fang Qin, Shirlene T.S. de Lima, Lito Papamichail, Rick Schraauwen, Julia Forato, Ingra M. Claro, Xinyi Hua, Leda M. Simões Mello, Dewy Mae Offermans, Monique M.A. Verstegen, Marjan Boter, Maikel P. Peppelenbosch, Anna Barbiero, Elisabetta Pagani, Harry L.A. Janssen, José A. Telmos Silva, Magnun N.N. dos Santos, Eder C. Pincinato, José Luiz Proenca-Modena, Pengfei Li, Adam A. Anas, Luc J. W. van der Laan, Concetta Castilletti, Bas B. Oude Munnink, William M. de Souza, Wenshi Wang, and Qiuwei Pan

Supplementary Information

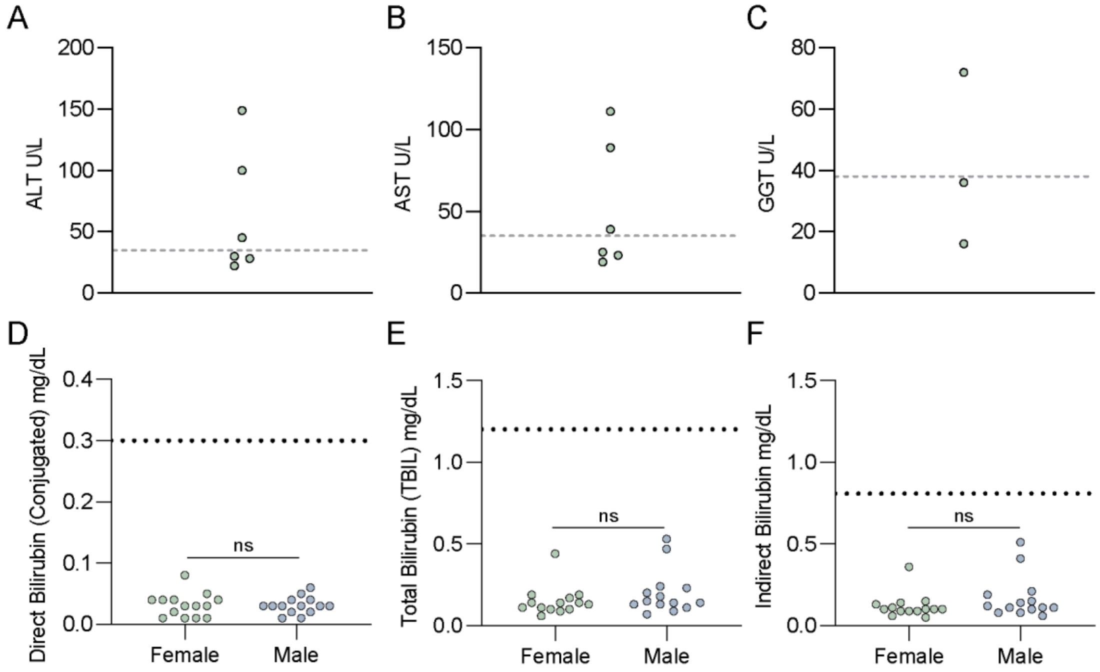

Figure S1. Liver enzyme and bilirubin levels in patients with OROV infection from Europe and Brazil. Related to Figure 1.

(A) ALT levels in OROV-infected cases imported to Europe with a threshold of 35 U/L. (B) AST levels with a threshold of 35 U/L. (C) GGT levels with a threshold of 38 U/L. (D) Direct bilirubin levels in OROV-infected cases recruited from a cohort in Brazil with a threshold of 0.3 mg/dL. (E) Total bilirubin levels with a threshold of 1.2 mg/dL. (F) Indirect bilirubin levels with a threshold of 0.8 mg/dL. The threshold references were showed as dotted line and provided by local hospitals or determined by the kit manufacturers. ALT: alanine aminotransferase; AST: aspartate aminotransferase; GGT: gamma-glutamyl transferase. Data represent measured values from individual patients. P values determined by Mann-Whitney U test. ns, not significant.

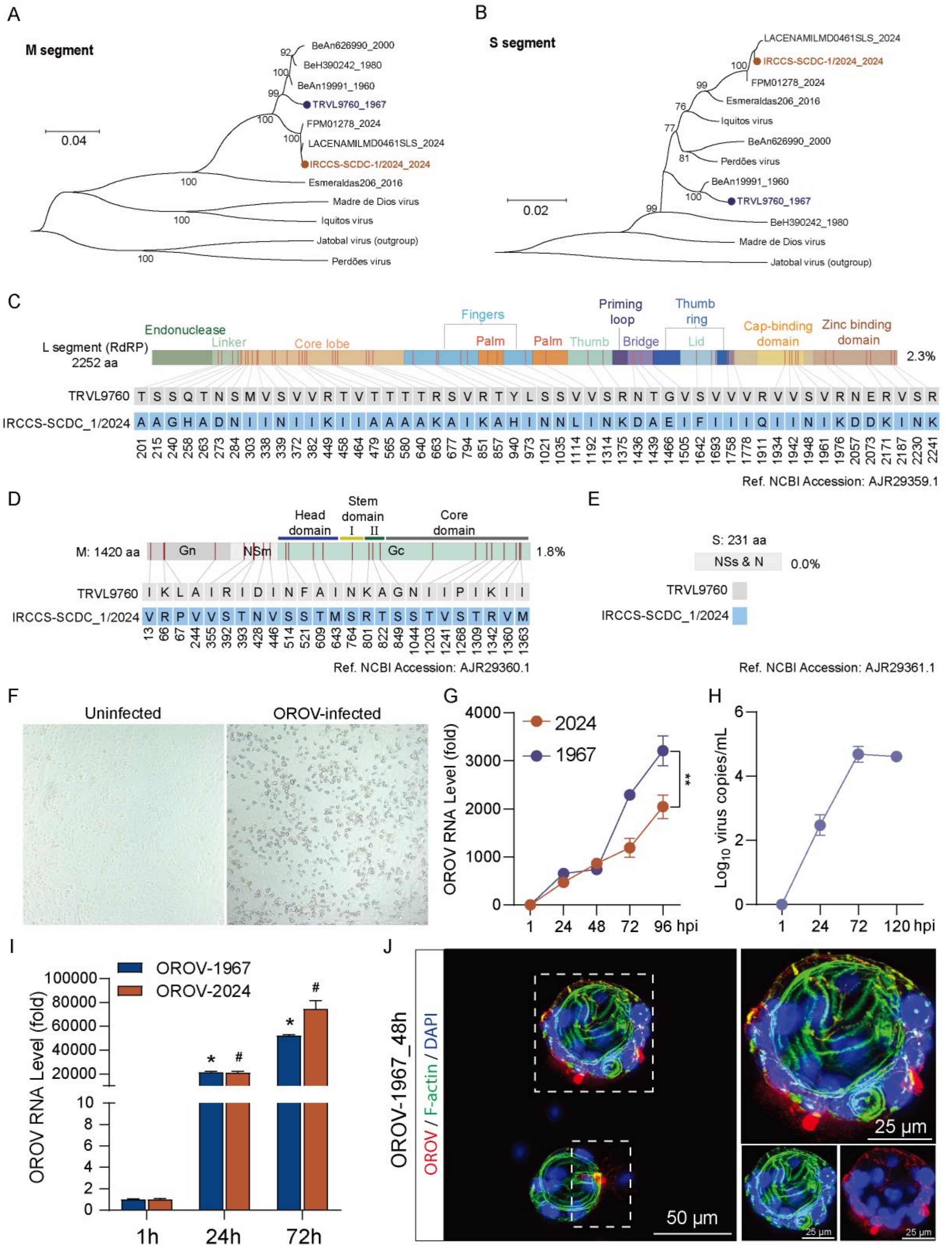

Figure S2. Productive infection of OROV in adult human liver-derived organoids. Related to Figure 2. (A, B) Phylogenetic analysis of OROV M (A) and S (B) segments. The neighbor-joining (NJ) trees were constructed using the p-distance model and evaluated with 1,000 bootstrap replicates. Only bootstrap values greater than 70% are shown. Trees are midpoint-rooted. (C–E) Amino acid substitutions between TRVL9760 and IRCCS-SCDC_1/2024 strains in the L (D), M (D), and S (E) segments. Only differing residues are shown. (F) Representative images of Vero cells showing OROV-induced cytopathic effects compared with uninfected controls. (G) Relative OROV RNA levels in organoids post-inoculation with the OROV-1967 and OROV-2024

isolates (0 in x axis represent 1-hour post-infection; n = 4 biological replicates). (H) Quantification of extracellular OROV RNA levels in cultured medium (n = 4 biological replicates). (I) Intracellular OROV RNA levels in human hepatoma-derived Huh7 cells infected with OROV-1967 or OROV-2024 at 1-, 24-, and 72-hours post-infection (n = 4 biological replicates). (J) Immunofluorescence images of organoids 48 hours post-inoculation with OROV-1967, stained with the antibody against OROV Gc glycoprotein (red), phalloidin for actin (green) and DAPI for nuclei (blue). Scale bar, 50 μm and 25 μm. Data represent mean ± SEM. Statistical analysis for longitudinal data were performed using mixed-effects model with restricted maximum likelihood (REML) (G). Statistical comparisons between different time points were performed using two-tailed Mann-Whitney U tests (I). $*p < 0.05$, $\#p < 0.05$, $**p < 0.01$.

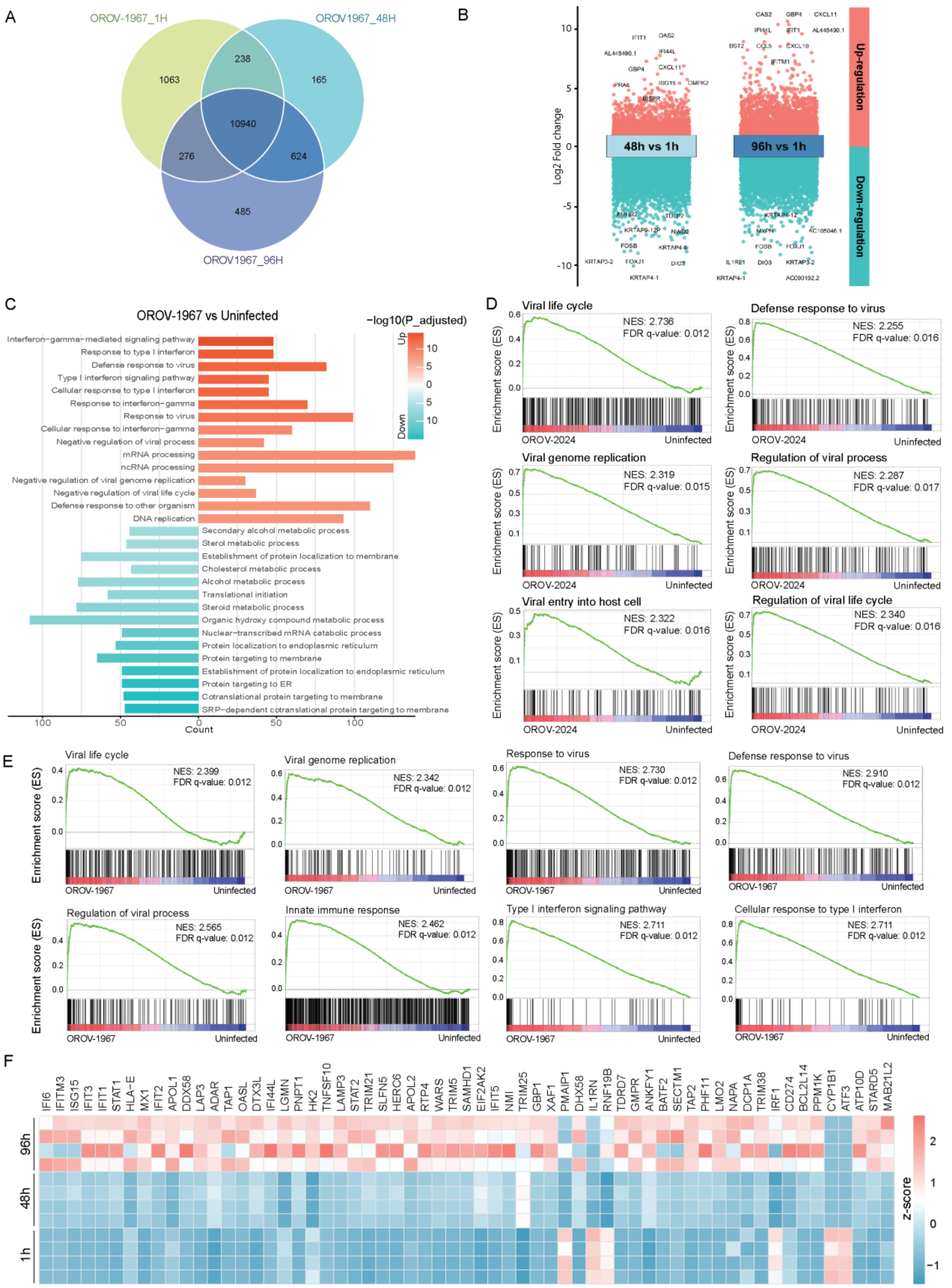

Figure S3. Transcriptomic analyses of host responses to OROV infection. Related to Figure 3.

(A) Venn diagram of overlapped differentially expressed genes in the OROV-1967 strain infected organoids at different time points. (B) Volcano plot analysis of differentially expressed genes at 48 and 96 hours compared to 1 hour after OROV-1967 inoculation. (C) Top 15 up-regulated and top 15 down-regulated pathways identified by gene ontology (GO) analysis in OROV-1967 infected organoids at 96 hours post-inoculation compared to the

uninfected controls cultured at the same condition for 96 hours. (D) Gene set enrichment analysis (GSEA) in OROV-2024 infected organoids at 96 hours post-inoculation compared to uninfected controls. (E) GSEA in OROV-1967 infected organoids at 96 hours post-inoculation compared to uninfected controls. (F) Heatmap showing the expression of selected interferon-stimulated genes (ISGs) in OROV-1967 infected organoids at 1, 48, and 96 hours post-inoculation. Data are representative of four biological replicates from one RNA-seq experiment using two independent OROV isolates and multiple time points. The color scale represents row-wise Z-score of gene expression across samples.

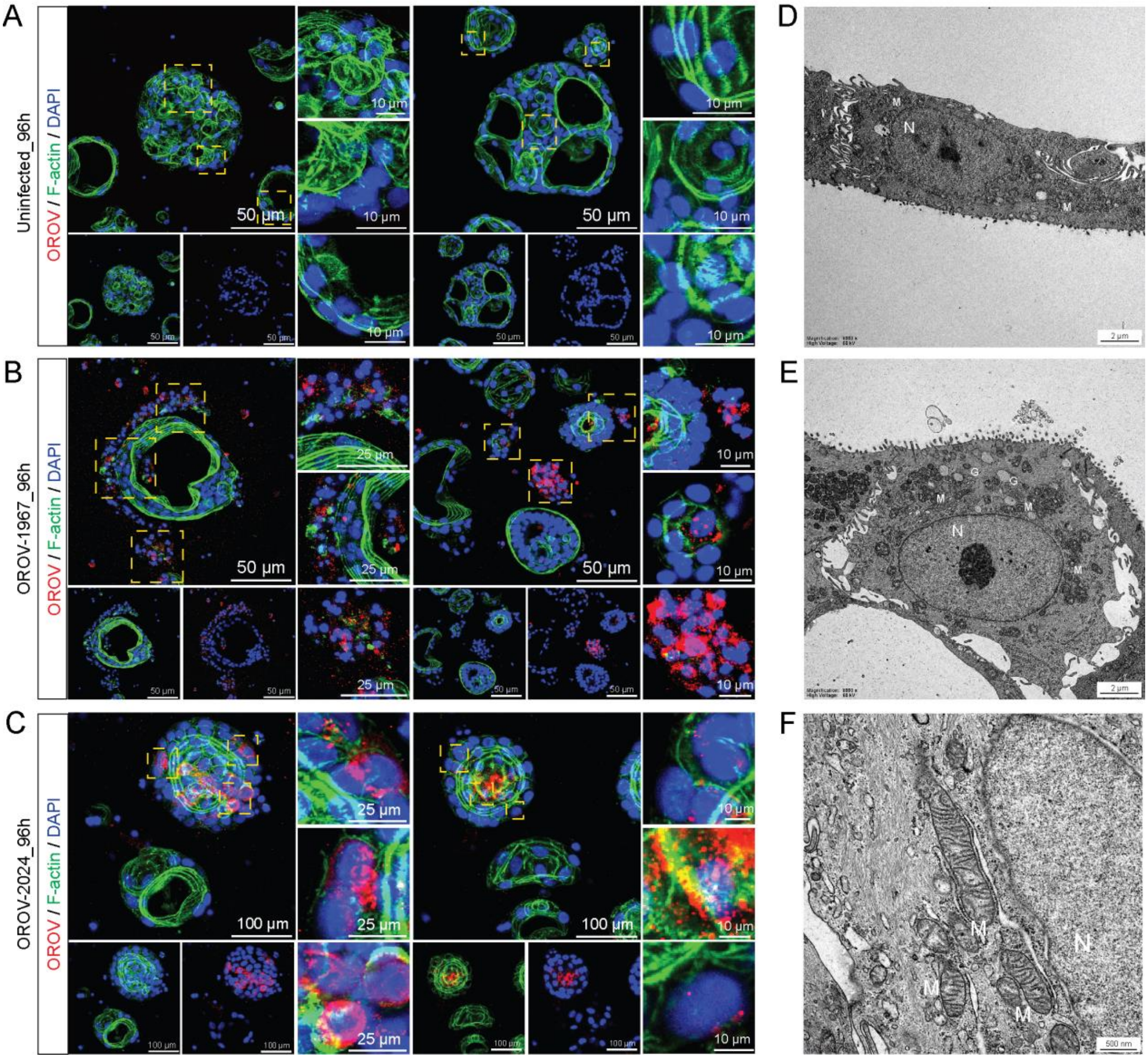

Figure S4. Cellular pathogenic alterations in OROV-infected organoids. Related to Figure 4.

(A-C) Immunofluorescence images of uninfected organoids (A), organoids 96 hours post-inoculation with OROV-1967 (B), and organoids 96 hours post-inoculation with OROV-2024 (C), stained with the antibody against OROV Gc glycoprotein (red), phalloidin (green) and DAPI nuclei staining (blue). Scale bar, 100, 50, 25 and 10 μm. (D-F) Representative TEM images visualized relatively intact mitochondria in organoids 96 hours post-inoculation with OROV-1967. Scale bars, 2 μm in D, E; 500 nm in F. N, nuclear. M, mitochondria. G, Golgi complex.

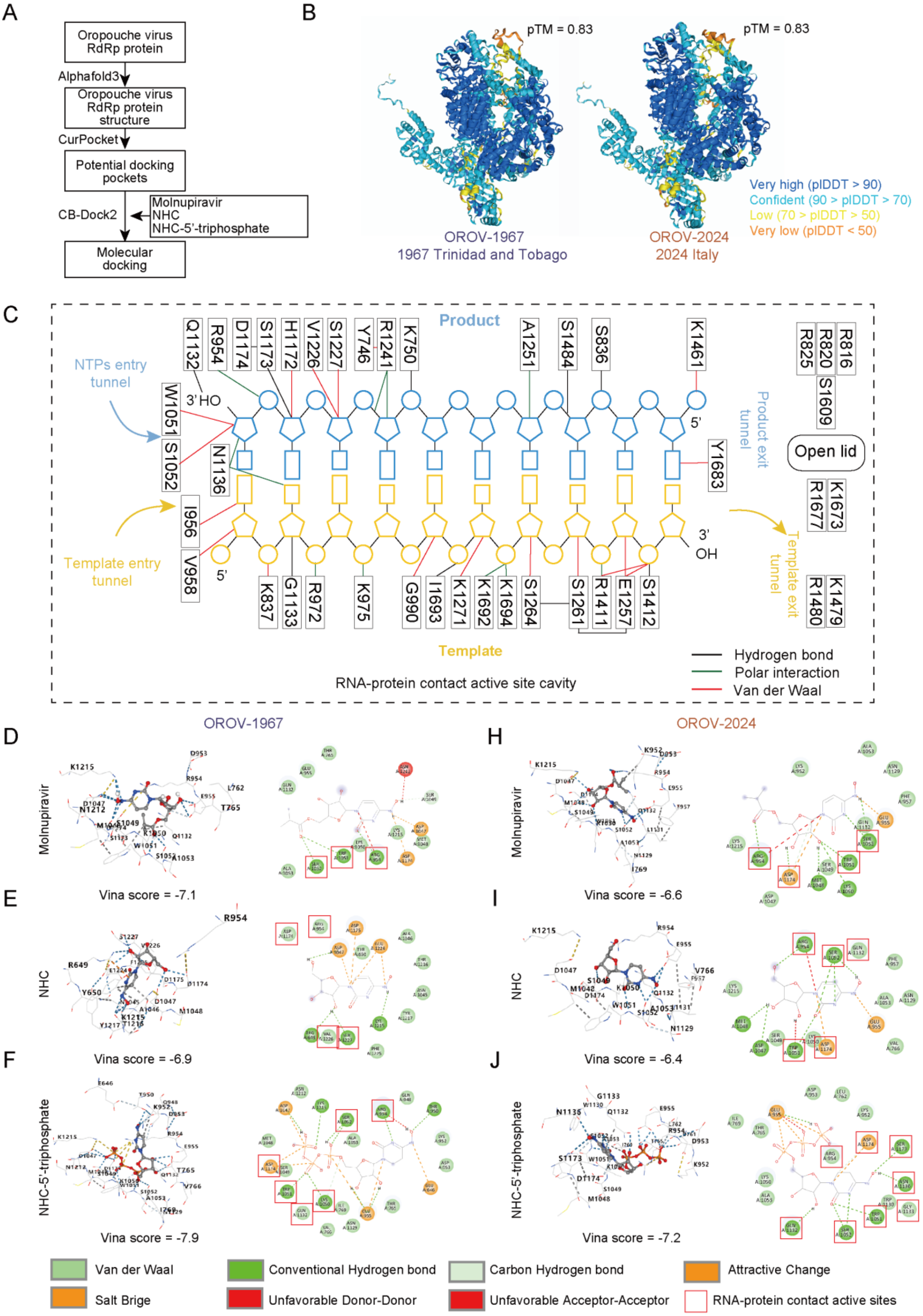

A
Oropouche virus RdRp protein
Alphafold3
Oropouche virus RdRp protein structure
CurPocket
Potential docking pockets
CB-Dock2
Molnupiravir
NHC
NHC-5'-triphosphate
Molecular docking
B
pTM = 0.83
pTM = 0.83
OROV-1967
1967 Trinidad and Tobago
OROV-2024
2024 Italy
Very high (pIDDT > 90)
Confident (90 > pIDDT > 70)
Low (70 > pIDDT > 50)
Very low (pIDDT < 50)
C
Product
NTPs entry tunnel
Template entry tunnel
Product exit tunnel
Template exit tunnel
Open lid
3' HO
5'
3' OH
Q1132
R954
D1174
S1173
H1172
V1226
S1227
Y746
R1241
K750
A1251
S1484
S836
K1461
R825
R820
R816
S1609
W1051
S1052
N1136
Y1683
R1677
K1673
I956
V958
K837
G1133
R972
K975
G990
I1693
K1271
K1692
K1694
S1264
S1261
R1411
E1257
S1412
R1480
K1479
Template
Hydrogen bond
Polar interaction
Van der Waal
RNA-protein contact active site cavity
OROV-1967
OROV-2024
D
H
Molnupiravir
Vina score = -7.1
Vina score = -6.6
E
I
NHC
Vina score = -6.9
Vina score = -6.4
F
J
NHC-5'-triphosphate
Vina score = -7.9
Vina score = -7.2
Van der Waal
Conventional Hydrogen bond
Carbon Hydrogen bond
Attractive Change
Salt Brige
Unfavorable Donor-Donor
Unfavorable Acceptor-Acceptor
RNA-protein contact active sites

Figure S5. Site specific binding mode of molnupiravir, NHC, and NHC-5'-triphosphate to OROV RdRp. Related to Figure 5.

(A) Workflow of this study. (B) The RdRp protein structures predicted by Aphafold3. (C) Schematic representation of RNA–protein contacts in the active site cavity. Drugs bind at the NTPs entry tunnel, interacting with key residues such as R954, W1051, S1052, Q1132, N1136, H1172, D1174. (D-F) The docking poses and binding residues of molnupiravir (D), NHC (E), and NHC-5'-triphosphate (F) with OROV RdRp protein of OROV-1967. (H-J) The docking poses and binding residues of molnupiravir (H), NHC (I), and NHC-5'-triphosphate (J) with OROV RdRp protein of OROV-2024.

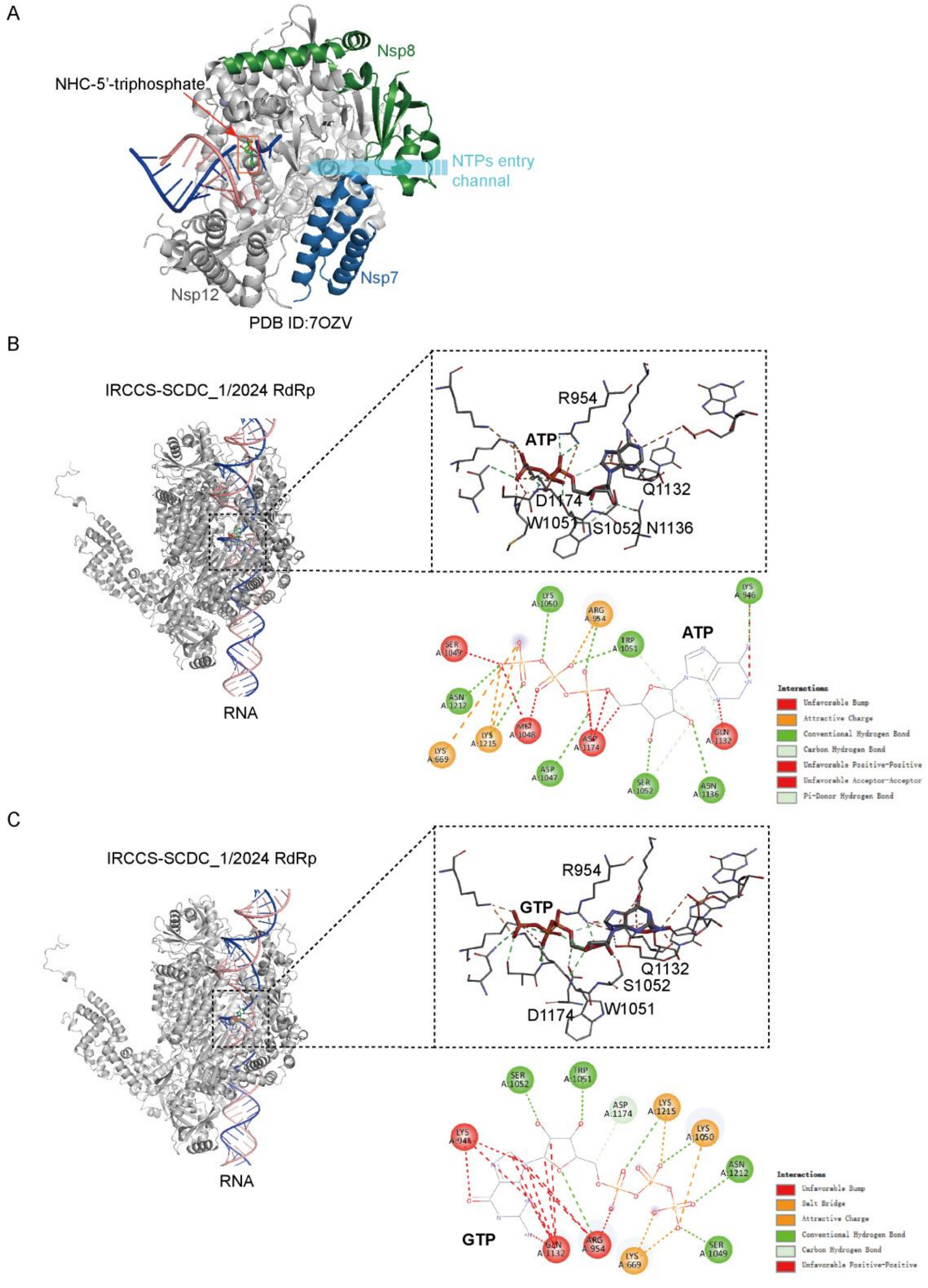

Figure S6. Schematic of molnupiravir's antiviral mechanism. Related to Figure 5.

(A) Crystal structure of SARS-CoV-2 RdRp bound to molnupiravir's active form, NHC-5′-triphosphate, in the template strand paired with G (PDB 7OZV).

(B, C) Docking poses of ATP (B) and GTP (C) in Oropouche virus (OROV) RdRp. AlphaFold3-guided docking of (B) ATP and (C) GTP into the IRCCS-SCDC_1/2024 RdRp key active sites (e.g., R954, W1051, S1052, Q1132, N1136, D1174).

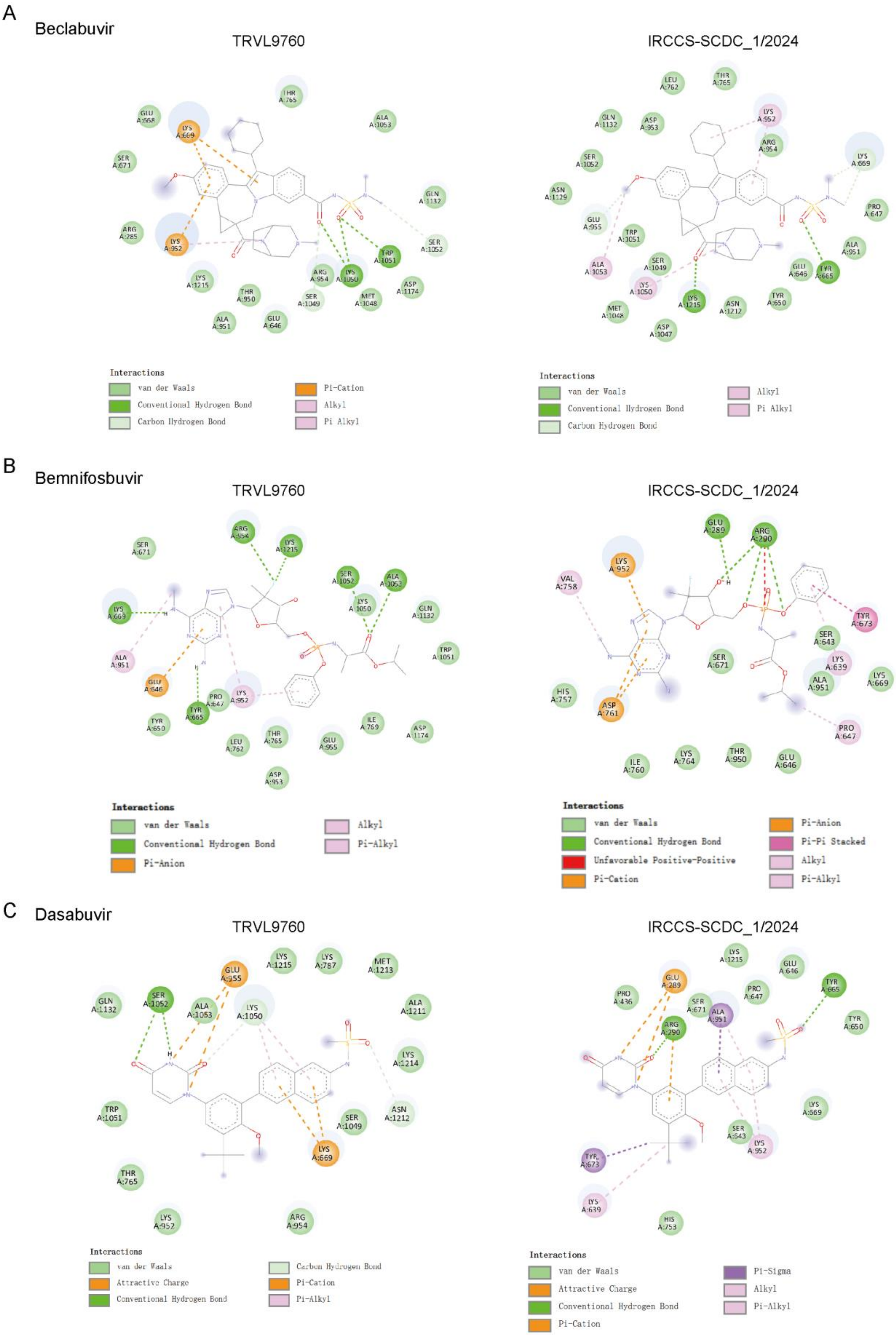

A
Beclabuvir
TRVL9760
IRCCS-SCDC_1/2024
Interactions
van der Waals
Conventional Hydrogen Bond
Carbon Hydrogen Bond
Pi-Cation
Alkyl
Pi Alkyl
Interactions
van der Waals
Conventional Hydrogen Bond
Carbon Hydrogen Bond
Alkyl
Pi Alkyl
B
Bemnifosbuvir
TRVL9760
IRCCS-SCDC_1/2024
Interactions
van der Waals
Conventional Hydrogen Bond
Pi-Anion
Alkyl
Pi-Alkyl
Interactions
van der Waals
Conventional Hydrogen Bond
Unfavorable Positive-Positive
Pi-Cation
Pi-Anion
Pi-Pi Stacked
Alkyl
Pi-Alkyl
C
Dasabuvir
TRVL9760
IRCCS-SCDC_1/2024
Interactions
van der Waals
Attractive Charge
Conventional Hydrogen Bond
Carbon Hydrogen Bond
Pi-Cation
Pi-Alkyl
Interactions
van der Waals
Attractive Charge
Conventional Hydrogen Bond
Pi-Cation
Pi-Sigma
Alkyl
Pi-Alkyl

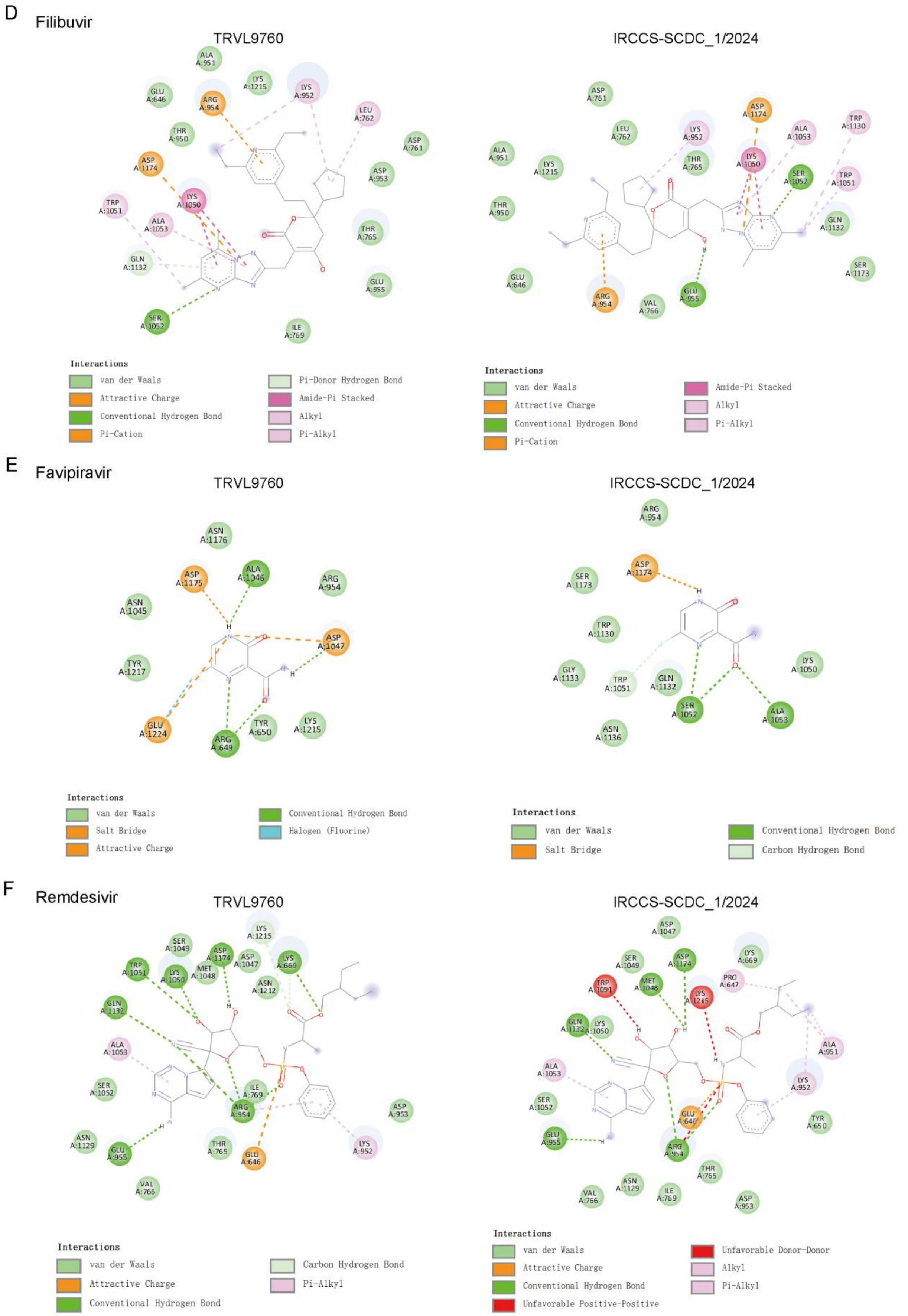
D
Filibuvir
TRVL9760
IRCCS-SCDC_1/2024
Interactions
van der Waals
Attractive Charge
Conventional Hydrogen Bond
Pi-Cation
Pi-Donor Hydrogen Bond
Amide-Pi Stacked
Alkyl
Pi-Alkyl
E
Favipiravir
TRVL9760
IRCCS-SCDC_1/2024
Interactions
van der Waals
Salt Bridge
Attractive Charge
Conventional Hydrogen Bond
Halogen (Fluorine)
Carbon Hydrogen Bond
F
Remdesivir
TRVL9760
IRCCS-SCDC_1/2024
Interactions
van der Waals
Attractive Charge
Conventional Hydrogen Bond
Carbon Hydrogen Bond
Pi-Alkyl
Unfavorable Positive-Positive
Unfavorable Donor-Donor
Alkyl

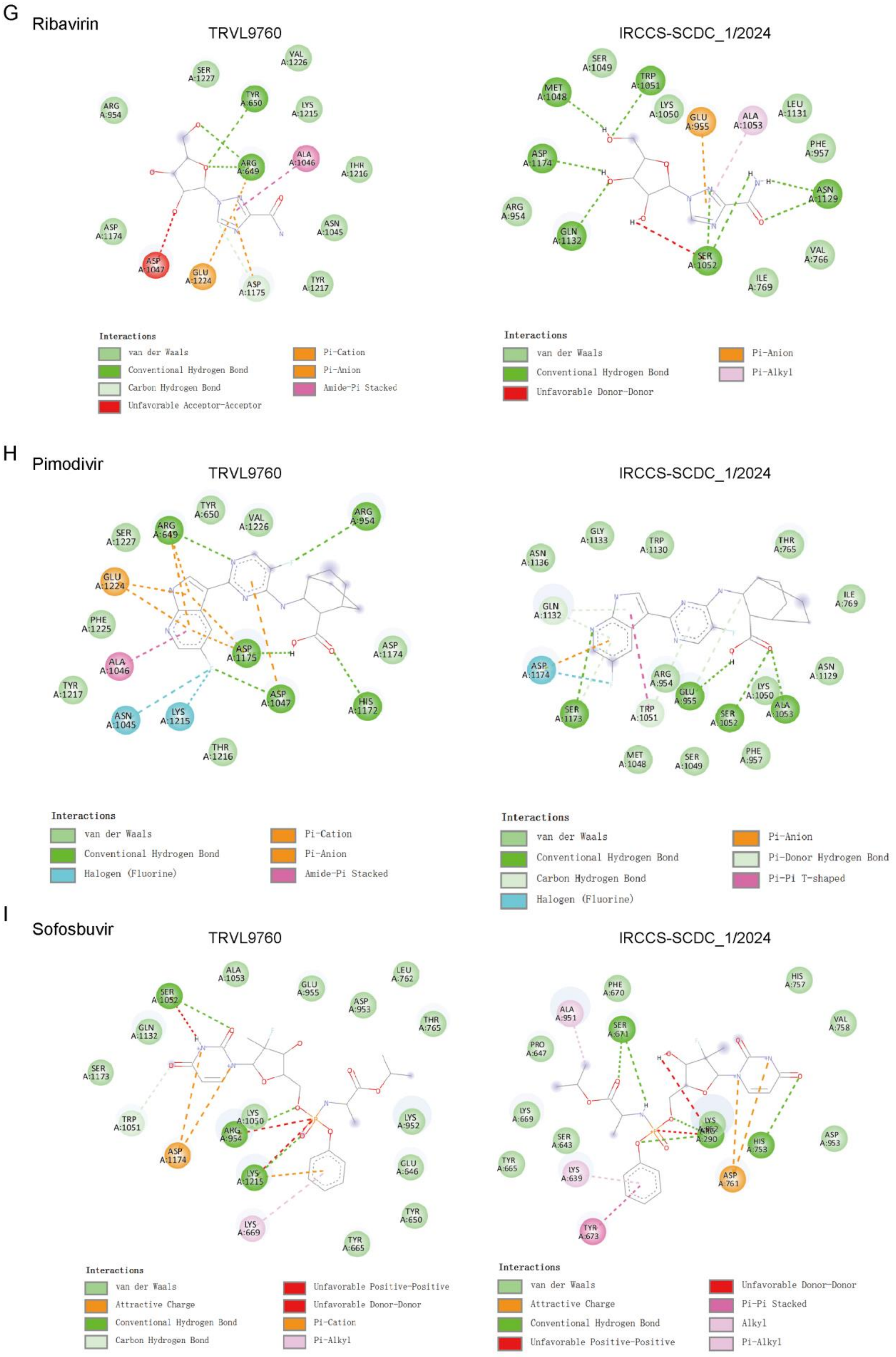

G
Ribavirin
TRVL9760
IRCCS-SCDC_1/2024
Interactions
van der Waals
Conventional Hydrogen Bond
Carbon Hydrogen Bond
Unfavorable Acceptor-Acceptor
Pi-Cation
Pi-Anion
Amide-Pi Stacked
Interactions
van der Waals
Conventional Hydrogen Bond
Unfavorable Donor-Donor
Pi-Anion
Pi-Alkyl
H
Pimodivir
TRVL9760
IRCCS-SCDC_1/2024
Interactions
van der Waals
Conventional Hydrogen Bond
Halogen (Fluorine)
Pi-Cation
Pi-Anion
Amide-Pi Stacked
Interactions
van der Waals
Conventional Hydrogen Bond
Carbon Hydrogen Bond
Halogen (Fluorine)
Pi-Anion
Pi-Donor Hydrogen Bond
Pi-Pi T-shaped
I
Sofosbuvir
TRVL9760
IRCCS-SCDC_1/2024
Interactions
van der Waals
Attractive Charge
Conventional Hydrogen Bond
Carbon Hydrogen Bond
Unfavorable Positive-Positive
Unfavorable Donor-Donor
Pi-Cation
Pi-Alkyl
Interactions
van der Waals
Attractive Charge
Conventional Hydrogen Bond
Unfavorable Positive-Positive
Unfavorable Donor-Donor
Pi-Pi Stacked
Alkyl
Pi-Alkyl

J

Tegobuvir

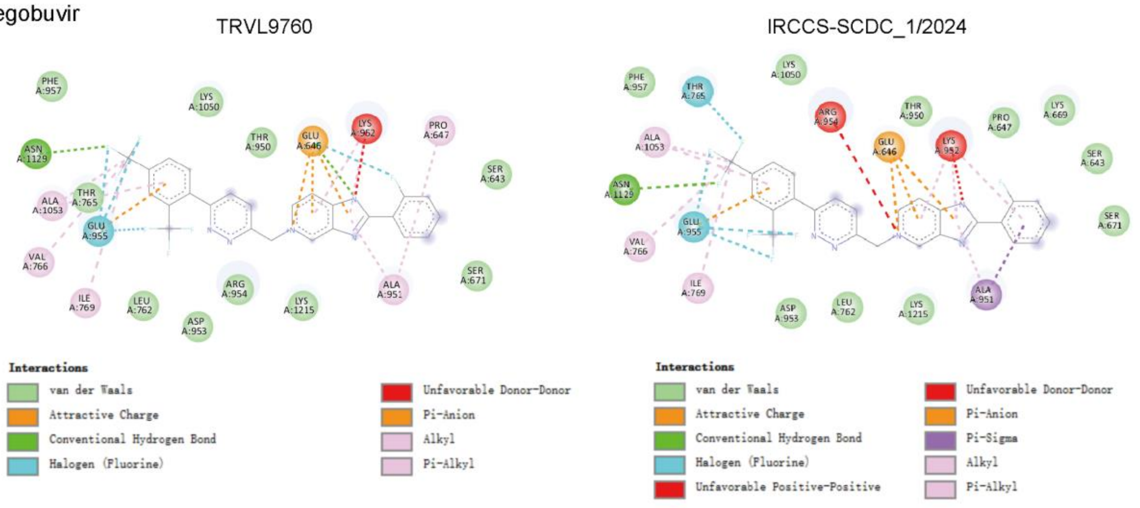

K

Prediction of antiviral drug binding characteristics and affinities to the RdRp of IRCCS-SCDC_1/2024 strain

Drugs	Category	Interaction with key residues*	CB-Dock2 Score	Calculated Affinity (kcal/mol)
NHC-5'-triphosphate	Nucleotide Analogue	R954, W1051, S1052, Q1132, N1136, S1173, D1174	-7.00	-7.06
Beclabuvir	Non-nucleoside RNA polymerase inhibitor	/	-9.00	-7.05
Bemnifosbuvir	Nucleoside RNA polymerase inhibitor	/	-8.00	-7.28
Dasabuvir	Non-nucleoside RNA polymerase inhibitor	/	-9.00	-8.21
Filibuvir	Non-nucleoside RNA polymerase inhibitor	R954, W1051, S1052, D1174	-8.90	-8.36
Favipiravir	Nucleotide Analogue	W1051, S1052, D1174	-5.90	-6.02
Remdesivir	Nucleotide Analogue	R954, W1051, Q1132, D1174	-8.10	-6.95
Ribavirin	Nucleotide Analogue	W1051, S1052, Q1132, D1174	-6.60	-6.36
Pimodivir	Non-nucleoside RNA polymerase inhibitor	W1051, S1052, Q1132, S1173, D1174	-8.50	-8.50
Sofosbuvir	Nucleoside RNA polymerase inhibitor	/	-7.80	-7.32
Tegobuvir	Non-nucleoside RNA polymerase inhibitor	R954	-9.30	-10.03

*Interactions listed represent specific contacts (e.g., hydrogen bonds, π–π stacking, salt bridges, or metal coordination) predicted by molecular docking. Non-specific van der Waals interactions were not listed.

Figure S7. Docking of antiviral drug candidates to OROV RdRp. Related to Figure 5.

(A-J) Molecular docking of (A) Beclabuvir, (B) Bemnifosbuvir, (C) Dasabuvir, (D) Filibuvir, (E) Favipiravir, (F) Remdesivir, (G) Ribavirin, (H) Pimodivir, (I) Sofosbuvir, and (J) Tegobuvir into the active site of Oropouche virus (OROV) RdRp. Explicit interactions, including hydrogen bonds, π–π stacking, salt bridges and metal coordination are indicated by dashed lines; residues depicted without dashed lines are within van der Waals contact distance (≤4 Å) of the ligand. (K) Summary of each compound's drug class, interaction with key residues, CB-Dock2 score (unitless), and calculated binding affinity (kcal/mol, from SwissDock using the AutoDock Vina engine) against IRCCS-SCDC_1/2024 OROV RdRp.

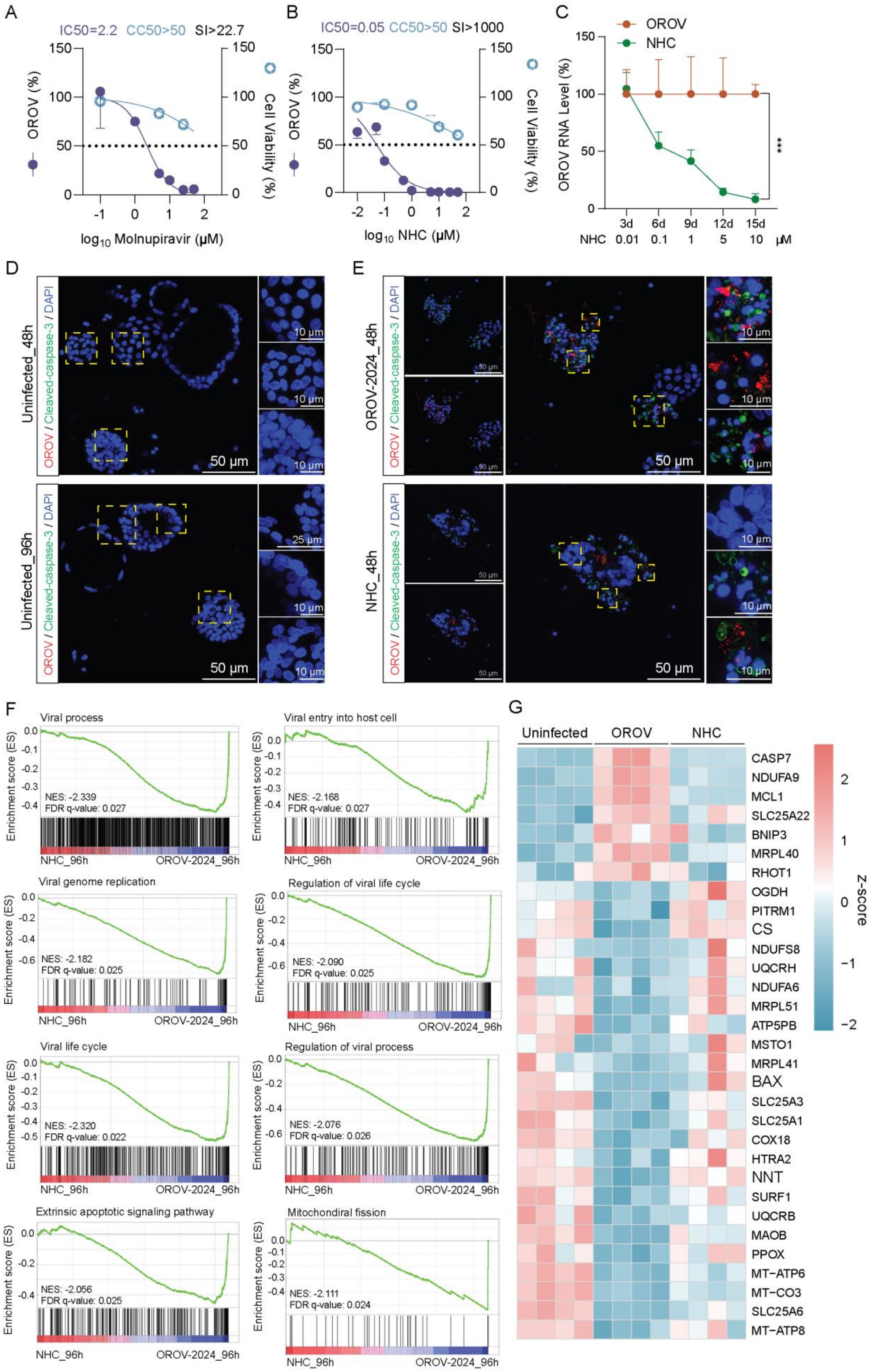
A
IC50=2.2 CC50>50 SI>22.7
OROV (%)
Cell Viability (%)
log10 Molnupiravir (μM)
B
IC50=0.05 CC50>50 SI>1000
OROV (%)
Cell Viability (%)
log10 NHC (μM)
C
OROV
NHC
OROV RNA Level (%)
***
3d 6d 9d 12d 15d
NHC 0.01 0.1 1 5 10 μM
D
Uninfected_48h
Uninfected_96h
OROV / Cleaved-caspase-3 / DAPI
50 μm
10 μm
25 μm
E
OROV-2024_48h
NHC_48h
OROV / Cleaved-caspase-3 / DAPI
50 μm
10 μm
F
Viral process
NES: -2.339
FDR q-value: 0.027
Viral entry into host cell
NES: -2.168
FDR q-value: 0.027
Viral genome replication
NES: -2.182
FDR q-value: 0.025
Regulation of viral life cycle
NES: -2.090
FDR q-value: 0.025
Viral life cycle
NES: -2.320
FDR q-value: 0.022
Regulation of viral process
NES: -2.076
FDR q-value: 0.026
Extrinsic apoptotic signaling pathway
NES: -2.056
FDR q-value: 0.025
Mitochondrial fission
NES: -2.111
FDR q-value: 0.024
Enrichment score (ES)
NHC_96h
OROV-2024_96h
G
Uninfected
OROV
NHC
CASP7
NDUFA9
MCL1
SLC25A22
BNIP3
MRPL40
RHOT1
OGDH
PITRM1
CS
NDUFS8
UQCRH
NDUFA6
MRPL51
ATP5PB
MSTO1
MRPL41
BAX
SLC25A3
SLC25A1
COX18
HTRA2
NNT
SURF1
UQCRB
MAOB
PPOX
MT−ATP6
MT−CO3
SLC25A6
MT−ATP8
z-score

Figure S8. Antiviral effect of molnupiravir in organoids inoculated with OROV. Related to Figure 5 & 6. (A, B) The 50% inhibitory concentration (IC50) and 50% cytotoxic concentration (CC50) of molnupiravir (A) or NHC (B) determined in the OROV-1967 strain infected organoids (n = 4 biological replicates). (C) Viral RNA levels in the supernatant of OROV-infected organoids during a 15-day NHC treatment using a stepwise dose-escalation approach, starting at 0.01 μM and increasing to 10 μM (n = 4 biological replicates). (D) Representative images of uninfected organoids at 48 (top) and 96 (bottom) hours by immunostaining with the antibody against OROV Gc glycoprotein (red), cleaved-caspase-3 (green) and nuclei (DAPI, blue). Scale bar, 50, 25 and 10 μm. (E) Representative images of OROV-2024 infected organoids without (top) or with (bottom) 48 hours NHC (1 μM) treatment by immunostaining with the antibody against OROV Gc glycoprotein (red), cleaved-caspase-3 (green) and nuclei (DAPI, blue). Scale bar, 50 μm. (F) Gene set enrichment analysis (GSEA) in OROV-2024 infected organoids with NHC treatment for 96 hours compared to organoids with OROV infection alone. (G) Heatmap showing the expression of selected mitochondrial-related genes comparing uninfected controls, OROV-infected organoids at 96 hours post-inoculation and OROV-infected organoids with 96 hour NHC treatment. Data are representative of four biological replicates from one RNA-seq experiment (F, G). Mitochondrial-related genes were sourced from an online-published gene set (GSEA-MSigDB) and NCBI gene. The color scale represents row-wise Z-score of gene expression across samples. Statistical analysis for longitudinal data were performed using mixed-effects model (REML). Data represent mean ± SEM; $***p < 0.001$.

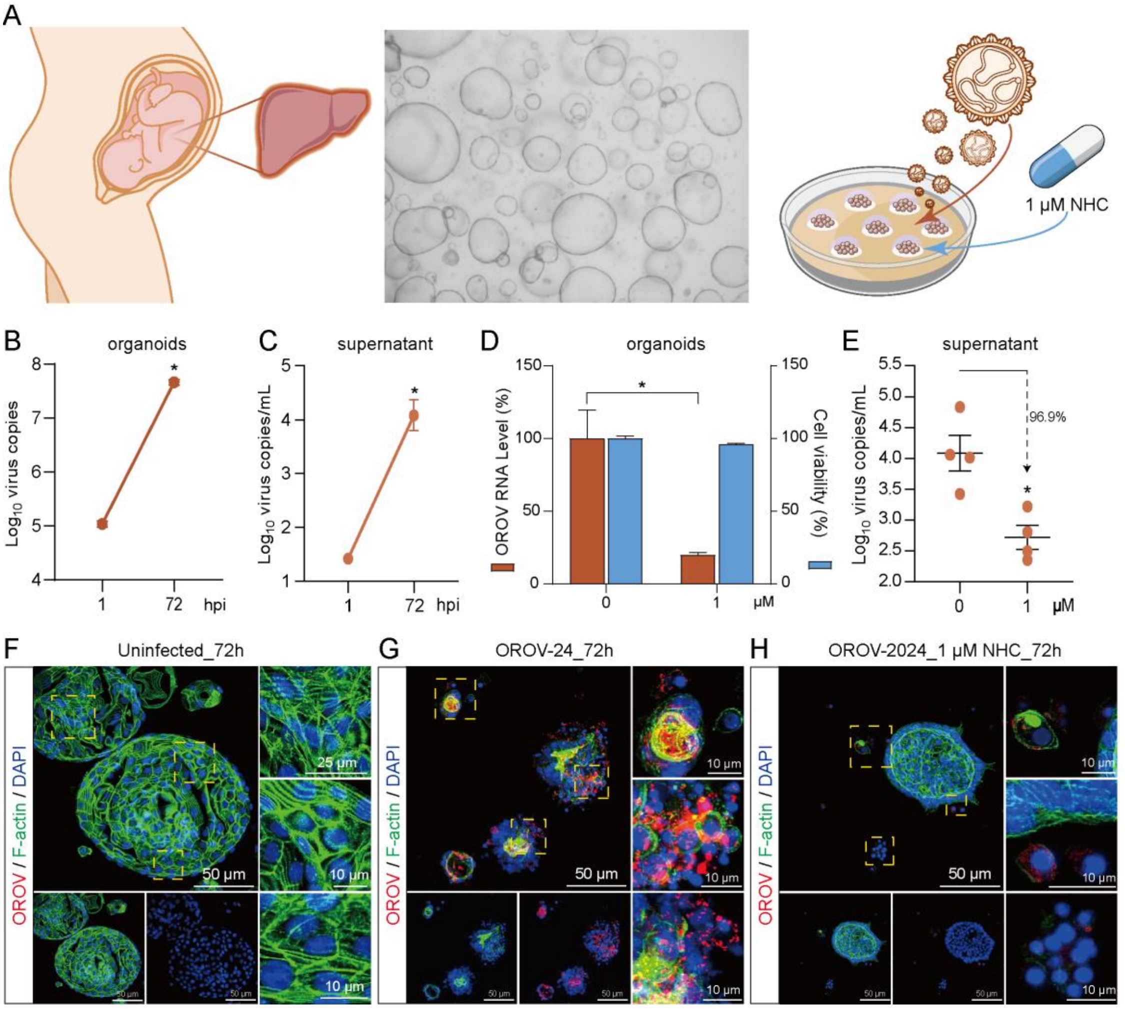

Figure S9. Human fetal liver-derived organoids support OROV infection but effectively inhibited by molnupiravir. Related to Figure 2 & 5.

(A) Schematic representation of fetal liver-derived organoids inoculated with OROV-2024 and 1 μM NHC treatment, and the bright field image of organoids. (B, C) Quantification of viral RNA copy number in organoids (B) and culture medium (C) after 1 hour and 72 hours inoculation with OROV-2024 (n = 4 biological replicates). (D) The inhibitory efficacy of 1 μM NHC on intracellular OROV RNA levels in organoids and corresponding cell viability after 72 hours treatment (n = 4 biological replicates). (E) The inhibitory efficacy of 1 μM NHC on OROV infectious titers in culture medium after 72 hours treatment (n = 4 biological replicates). (F-H) Representative images of uninfected organoids (F), OROV-2024 infected organoids without (G) or with (H) 72 hours NHC (1 μM) treatment by immunostaining with the antibody against OROV Gc glycoprotein (red), phalloidin for F-actin (green) and DAPI for nuclei (blue). Scale bar, 50, 25 and 10 μm. Data represent mean ± SEM; Statistical analysis by Mann-Whitney U test; $*p < 0.05$; ns, not significant.

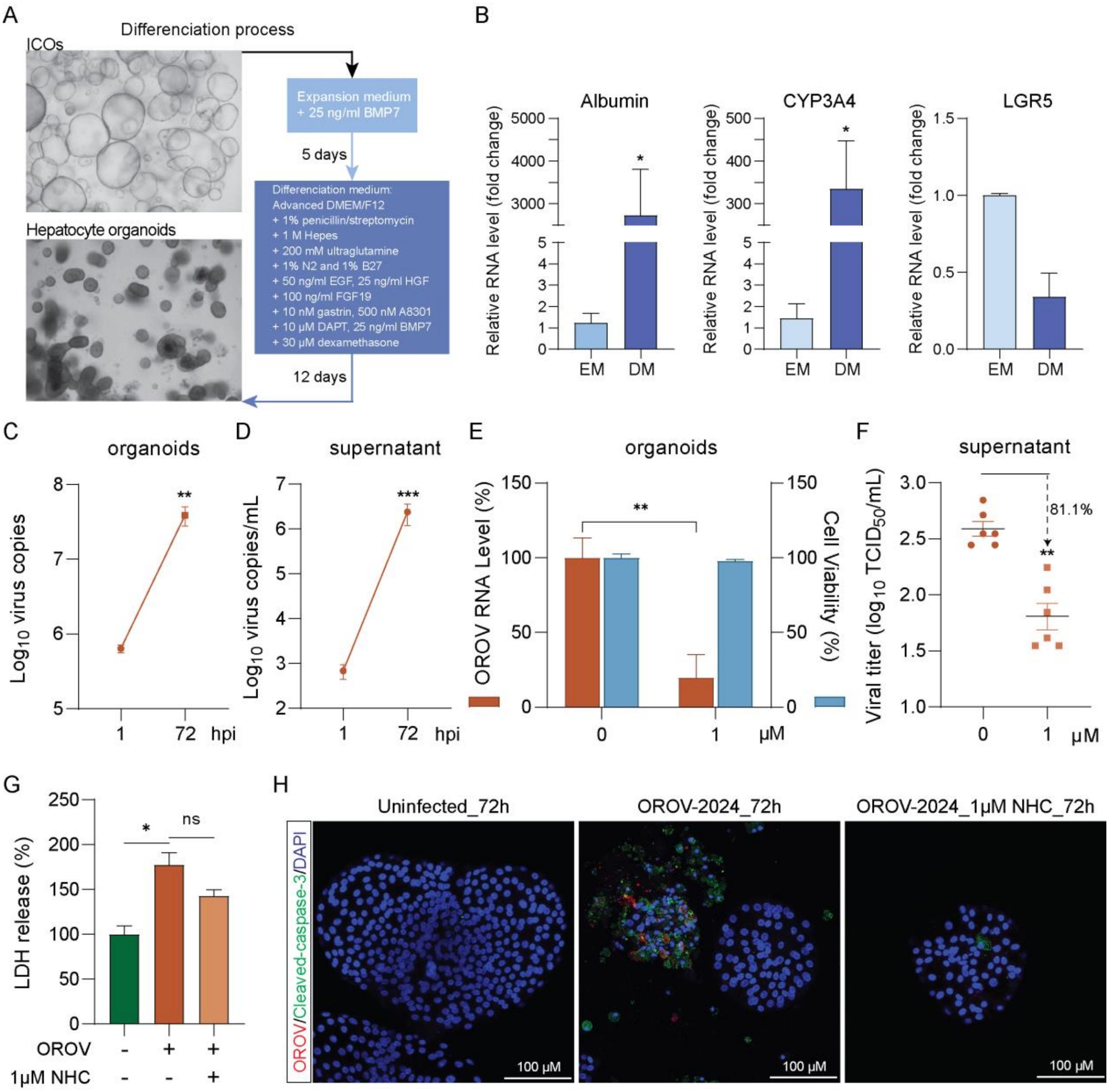

Figure S10. Hepatocyte-differentiated organoids are permissive to OROV infection and responsive to molnupiravir treatment. Related to Figure 2 & 5.

(A) Schematic of the differentiation protocol used to generate hepatocyte-differentiated organoids from intrahepatic cholangiocyte organoids (ICOs). (B) Quantification of hepatic marker genes (Albumin, CYP3A4) (n = 4 biological replicates from 2 donors) and progenitor marker (LGR5) (EM group: n = 4 biological replicates from 2 donors; DM group: n = 3 biological replicates from 2 donors) in organoids cultured in expansion medium (EM) or hepatocyte differentiation medium (DM). (C, D) Quantification of viral RNA copy number in organoids (C) (n = 6 biological replicates from 2 donors) and culture medium (D) (n = 10 biological replicates from 2 donors) after 1 hour and 72 hours inoculation with OROV-2024. (E) The inhibitory efficacy of 1 μM NHC on intracellular OROV RNA levels in organoids (n = 6 biological replicates from 2 donors) and corresponding cell viability (n = 8 biological replicates from 2 donors) after 72 hours treatment. (F) Infectious viral titers in culture supernatant from organoids treated with NHC at 48 and 96 hours (n = 6 biological replicates from 2 donors). (G) LDH release in culture supernatants of organoids at 72 hours post-inoculation, comparing uninfected, OROV-infected, and NHC-treated groups (1 μM) (n = 4 biological replicates from 2 donors). (H) Representative images

of uninfected organoids, OROV-2024 infected organoids without or with 72 hours NHC (1 μM) treatment by immunostaining with the antibody against OROV Gc glycoprotein (red), cleaved-caspase-3 (green) and DAPI for nuclei (blue). Scale bar, 100 μm. Data represent mean ± SEM; Statistical analysis by Mann-Whitney U test; $*p < 0.05$; $**p < 0.01$; $***p < 0.001$; ns, not significant.

Table S1. Characteristics and liver function tests for imported Oropouche cases in Europe. **Related to Figure 1.**

Case	Days from Symptoms Onset	OROV RT-PCR (Ct)	ALT (U/L)	AST (U/L)	GGT (U/L)	Age	Sex	Country	Reference
1	5	34.9	45	39	16	27	F	Italy	(Castilletti, Mori et al. 2024)
2	7	36.8	28	25	NA	55	F	Italy	(Castilletti, Mori et al. 2024)
3	18	35.4	22	23	NA	60	F	Italy	(Barbiero, Formica et al. 2025)
4	7	34.2	30	19	36	67	M	Italy	(Barbiero, Formica et al. 2025)
5	5	36.1	100	89	NA	42	M	Italy	Unpublished
6	6	32.4	149	111	72	41	M	Netherlands	(Igloi, Soochit et al. 2025)

Note: OROV RT-PCR was based on whole blood for the five cases in Italy, but was based on serum for the case in the Netherlands. All the cases were imported to Europe in 2024. Five of the six cases were reported in the previous studies, but the liver enzyme data were newly collected and were not reported previously. ALT: alanine aminotransferase; AST: aspartate aminotransferase; GGT: gamma-glutamyl transferase; NA: not available; F: female; M: male.

Table S2. Characteristics and liver function tests from a cohort of patients with Oropouche fever in Brazil. **Related to Figure 1.**

ID	Age	Sex	OROV RNA ct value	Alanine transaminase (ALT)	Aspartate aminotransferase (AST)	Gamma-glutamyl transferase (GGT)	Alkaline Phosphatase (ALP)	Direct Bilirubin (Conjugated)	Total Bilirubin	Indirect Bilirubin	Symptom onset date
1	53	FEMALE	25	48	55	9	41	0.01	0.1	0.09	2
2	41	MALE	24	36	38	38	65	0.03	0.24	0.21	3
3	49	FEMALE	21	96	98	47	73	0.05	0.19	0.14	2
4	55	MALE	25	55	55	13	59	0.03	0.14	0.11	0
5	31	MALE	20	27	16	27	41	0.01	0.07	0.06	1
6	27	FEMALE	22	23	23	18	69	0.03	0.09	0.06	2
7	76	MALE	18	41	38	18	48	0.02	0.13	0.11	4
8	30	FEMALE	20	89	72	64	71	0.01	0.1	0.09	2
9	40	FEMALE	26	61	52	21	67	0.03	0.13	0.1	1
10	22	FEMALE	21	35	34	18	47	0.04	0.17	0.13	2
11	60	MALE	19	21	21	23	74	0.02	0.53	0.51	3
12	54	FEMALE	18	12	14	12	30	0.02	0.11	0.09	1
13	24	MALE	20	47	41	22	53	0.03	0.13	0.1	2
14	74	FEMALE	22	71	65	106	65	0.08	0.44	0.36	1
15	73	MALE	24	31	30	43	75	0.03	0.15	0.12	3
16	65	FEMALE	25	11	15	20	38	0.04	0.19	0.15	4
17	23	MALE	25	59	74	45	54	0.03	0.11	0.08	0
18	21	FEMALE	23	31	32	24	58	0.01	0.11	0.1	0
19	66	MALE	21	73	56	236	95	0.04	0.23	0.19	1
20	31	MALE	23	21	22	16	57	0.04	0.18	0.14	1
21	31	FEMALE	23	34	21	40	43	0.01	0.06	0.05	1
22	26	MALE	27	31	33	23	59	0.03	0.14	0.11	1
23	52	MALE	23	71	64	67	112	0.06	0.47	0.41	1
24	56	MALE	24	21	23	113	109	0.05	0.2	0.15	1
25	75	FEMALE	23	96	130	73	91	0.04	0.14	0.1	2
26	60	FEMALE	25	73	73	288	100	0.03	0.14	0.11	1
27	48	MALE	25	9	25	20	36	0.01	0.09	0.08	2
28	54	FEMALE	27	68	71	31	45	0.04	0.14	0.1	3
29	55	MALE	25	NA	62	44	331	NA	NA	NA	1
30	47	MALE	18	85	70	58	225	NA	NA	NA	2

31	60	MALE	19	NA	58	39	NA	NA	NA	NA	3
32	73	MALE	24	NA	NA	530	601	NA	NA	NA	3
33	44	MALE	24	21	29	26	208	NA	NA	NA	1
34	59	MALE	27	NA	NA	52	NA	NA	NA	NA	3
35	56	MALE	24	NA	34	24	248	NA	NA	NA	1
36	74	FEMALE	22	191	215	101	362	NA	NA	NA	1
37	54	FEMALE	18	136	141	71	NA	NA	NA	NA	1
38	65	FEMALE	25	102	80	68	344	NA	NA	NA	4
39	21	FEMALE	23	57	63	10	123	NA	NA	NA	1
40	62	FEMALE	27	29	44	31	443	NA	NA	NA	1
41	31	FEMALE	23	143	106	35	346	NA	NA	NA	1
42	60	FEMALE	25	63	32	56	181	NA	NA	NA	1

Note: Clinical and laboratory characteristics of patients with Oropouche fever diagnosed during the 2024 outbreak in Brazil (July–August). Patients were matched by age, sex, and the time interval between symptom onset and sample collection. All cases were confirmed by real-time RT-qPCR targeting OROV, with a limit of detection of 2–20 copies, corresponding to Ct values ranging from 34.8 to 38.1.

Reference ranges for liver function tests were provided by Wiener Laboratories and are as follows: alanine aminotransferase (ALT), ≤41 U/L for males and ≤31 U/L for females; aspartate aminotransferase (AST), ≤38 U/L for males and ≤32 U/L for females; gamma-glutamyl transferase (GGT), 11–50 U/L for males and 7–32 U/L for females; alkaline phosphatase (ALP), ≤240 U/L for both sexes; total bilirubin, ≤1.2 mg/dL; direct bilirubin, ≤0.3 mg/dL; indirect bilirubin, ≤0.8 mg/dL. NA: not available.

Table S3. Summary of minor variant analysis in OROV genomes after serial passaging with or without NHC treatment. **Related to Figure 7.**

Segment	Position*	Nt change	AA change	Frequency in input	Frequency in CTR1	Frequency in CTR2	Frequency in CTR3	Frequency in NHC1	Frequency in NHC2	Frequency in NHC3
L	5498	T>C	-	39.5%	25.0%	27.2%	28.6%	47.5%	-	44.1%
L	5498	C>T	-	-	-	-	-	-	48.0%	-
M	556	G>A	V168I	41.9%	-	41.7%	33.7%	31.7%	47.4%	39.8%
M	556	G>A	I168V	-	40.8%	-	-	-	-	-
M	1684	G>T	A544T	42.7%	-	39.8%	42.8%	20.8%	26.3%	30.7%
M	1684	T > G	T544A	42.7%	40.2%	-	-	-	-	-
M	1892-1894	+GAC	Δ	-	27.9%	29.0%	27.0%	-	-	-
M	1892-1894	+CAA	Δ	-	-	-	-	-	21.6%	20.2%
M	2096	G>A	G680E	-	-	-	-	32.8%	28.9%	26.7%
M	3909	G>A	-	-	41.7%	42.4%	41.2%	-	-	-
M	3960	G>A	-	36.6%	35.9%	36.3%	34.3%	35.6%	40.0%	35.7%
S	66	C>T	-	-	29.0%	25.2%	22.8%	-	-	-
S	66	T>C	-	-	-	-	-	43.9%	40.2%	39.9%
S	375	T>A	-	-	25.1%	27.4%	23.4%	-	-	-
S	558	C>T	-	-	25.4%	27.5%	25.6%	-	-	-
S	567	C>T	-	-	25.2%	26.1%	24.4%	-	-	-

Note: Nucleotide and amino acid substitutions identified by Nanopore sequencing across L, M, and S segments of OROV. Only minor variants with a frequency over 20% are shown. Frequencies in the input virus, control passages (CTR1–CTR3), and NHC-treated passages (NHC1–NHC3) are presented. L, large segment encoding RNA-dependent RNA polymerase; M, medium segment encoding glycoproteins (Gn, Gc) and nonstructural protein; S, small segment encoding nucleocapsid protein N and the nonstructural protein; Nt, nucleotide; AA, amino acid; Δ, deletion

References

[1] GSEA-MSigDB. "https://www.gsea-msigdb.org/gsea/msigdb/human/geneset/MITOCHONDRIAL_PART.html."

[2] Barbiero, A., G. Formica, R. P. Mantovani, S. Accordini, F. Gobbi, M. Spinicci, M. G. Colao, S. Pollini, N. Ciccone, G. M. Rossolini, A. Bartoloni, L. Zammarchi and C. Castilletti (2025). "Persistent Oropouche virus viremia in two travellers returned to Italy from Cuba, July 2024." J Travel Med **32**(2).

[3] Castilletti, C., A. Mori, A. Matucci, N. Ronzoni, L. Van Duffel, G. Rossini, P. Sponga, M. L. D'Errico, P. Rodari, F. Cristini, R. Huits and F. G. Gobbi (2024). "Oropouche fever cases diagnosed in Italy in two epidemiologically non-related travellers from Cuba, late May to early June 2024." Euro Surveill **29**(26).

[4] Igloi, Z., W. Soochit, B. B. O. Munnink, A. A. Anas, K. J. von Eije, A. van der Linden, M. Mandigers, K. Wijnans, J. Voermans, F. D. Chandler, A. A. van der Eijk, C. GeurtsvanKessel, R. Molenkamp, R. S. Sikkema, B. Verstrepen and M. Koopmans (2025). "Oropouche Virus Genome in Semen and Other Body Fluids from Traveler." Emerg Infect Dis **31**(1): 205-206.
