# Supplementary material for: Human liver-derived organoids recapitulate Oropouche virus infection and manifestation, enabling antiviral drug discovery
Source: Cell Rep Med. 2026 Mar 9;7(3):102646. doi: 10.1016/j.xcrm.2026.102646 (PMC13006397; doi:10.1016/j.xcrm.2026.102646)
Supplement: Document S2. Article plus supplemental information [file mmc2.pdf]

Cell Reports
Medicine

Article

Human liver-derived organoids recapitulate Oropouche virus infection and manifestation, enabling antiviral drug discovery

Graphical abstract

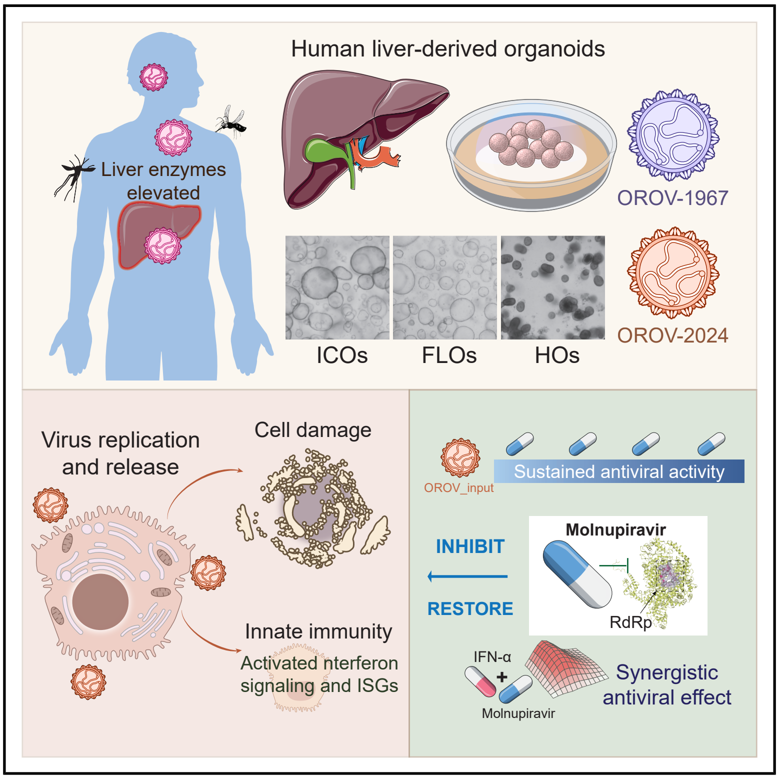

Highlights

- Elevated liver enzymes are detected in a subset of Oropouche patients
- OROV infects human liver organoids, triggering interferon response and cellular injury
- Molnupiravir strongly inhibits OROV infection and restores cellular homeostasis
- The combination of molnupiravir with interferon-alpha exhibits robust synergy

Authors

Jiajing Li, Xin Wang, Yibo Ding, ..., William M. de Souza, Wenshi Wang, Qiuwei Pan

Correspondence

wmdesouza@uky.edu (W.M.d.S.),
wenshi.wang@xzhmu.edu.cn (W.W.),
q.pan@erasmusmc.nl (Q.P.)

In brief

Li et al. integrate liver function data from Oropouche virus (OROV)-infected patients with human liver-derived organoids to demonstrate hepatic infection and resulting cellular injury. Using organoids, they further show that molnupiravir exerts strong antiviral activity against OROV and synergizes with interferon-alpha.

Li et al., 2026, Cell Reports Medicine 7, 102646
March 17, 2026 © 2026 The Author(s). Published by Elsevier Inc.
https://doi.org/10.1016/j.xcrm.2026.102646

CellPress

Cell Reports Medicine

CellPress
OPEN ACCESS

Article

Human liver-derived organoids recapitulate Oropouche virus infection and manifestation, enabling antiviral drug discovery

Jiajing Li,[1,19] Xin Wang,[1,19] Yibo Ding,[2] Fang Qin,[2] Shirlene T.S. de Lima,[3,4,5] Lito Papamichail,[6,7] Rick Schraauwen,[8] Julia Forato,[5] Ingra M. Claro,[3] Xinyi Hua,[3] Leda M. Simões Mello,[4] Dewy Mae Offermans,[1] Monique M.A. Verstegen,[6] Marjan Boter,[9] Maikel P. Peppelenbosch,[1] Anna Barbiero,[10] Elisabetta Pagani,[11] Harry L.A. Janssen,[1,12] José A. Telmos Silva,[13] Magnun N.N. dos Santos,[14] Eder C. Pincinato,[14] José Luiz Proenca-Modena,[5] Pengfei Li,[1] Adam A. Anas,[15,16] Luc J.W. van der Laan,[6] Concetta Castilletti,[17] Bas B. Oude Munnink,[9] William M. de Souza,[3,18,*] Wenshi Wang,[2,18,*] and Qiuwei Pan[1,18,20,*]

[1]Department of Gastroenterology and Hepatology, Erasmus MC-University Medical Center, Rotterdam, the Netherlands
[2]Department of Pathogen Biology and Immunology, Jiangsu Key Laboratory of Immunity and Metabolism, Jiangsu International Laboratory of Immunity and Metabolism, Xuzhou Medical University, Xuzhou 221004, China
[3]Department of Microbiology, Immunology, and Molecular Genetics, College of Medicine, University of Kentucky, Lexington, KY, USA
[4]Laboratório Central de Saúde Pública do Ceará, Fortaleza, Brazil
[5]Laboratory of Emerging Viruses, Department of Genetics, Microbiology and Immunology, Institute of Biology, University of Campinas, Campinas, Brazil
[6]Department of Surgery, Erasmus MC Transplant Institute, University Medical Center Rotterdam, Rotterdam, the Netherlands
[7]Department of Internal Medicine, Erasmus MC Transplant Institute, University Medical Center Rotterdam, Rotterdam, the Netherlands
[8]Department of Pathology, Erasmus MC-University Medical Center, Rotterdam, the Netherlands
[9]Department of Viroscience, Erasmus MC-University Medical Center, Rotterdam, the Netherlands
[10]Experimental and Clinical Medicine Department, University of Florence, Florence, Italy
[11]Laboratory of Microbiology and Virology, Provincial Hospital of Bolzano (SABES-ASDAA), Lehrkrankenhaus der Paracelsus Medizinischen Privatuniversität, Bolzano, Italy
[12]Toronto Centre for Liver Disease, Toronto General Hospital, University Health Network, Toronto, ON, Canada
[13]Laboratório do Hospital da Mulher de Fortaleza, Fortaleza, Brazil
[14]Department of Clinical Pathology, School of Medical Sciences, University of Campinas, Campinas, Brazil
[15]Department of Medical Microbiology and Infectious Diseases, Erasmus MC-University Medical Center, Rotterdam, the Netherlands
[16]Department of Internal Medicine, Section of Infectious Diseases, Erasmus MC-University Medical Center, Rotterdam, the Netherlands
[17]Department of Infectious-Tropical Diseases and Microbiology, IRCCS Sacro Cuore Don Calabria Hospital, Negrar di Valpolicella, 37024 Verona, Italy
[18]Senior author
[19]These authors contributed equally
[20]Lead contact
*Correspondence: wmdesouza@uky.edu (W.M.d.S.), wenshi.wang@xzhmu.edu.cn (W.W.), q.pan@erasmusmc.nl (Q.P.)
https://doi.org/10.1016/j.xcrm.2026.102646

SUMMARY

Oropouche virus (OROV) is a neglected, re-emerging arbovirus that typically causes self-limiting febrile illness but can also lead to severe complications. With no approved vaccines or treatments available, we integrate clinical data with human liver-derived organoids to assess liver involvement in OROV infection and identify antiviral candidates through drug repurposing. Patient blood tests show elevated liver enzymes, indicating OROV-associated hepatic dysfunction. OROV isolates productively infect liver organoids and induce severe cellular damage. Transcriptomic profiling reveals strong virus-host interactions, including activation of interferon-stimulated genes and cell death pathways. Pharmacological inhibition of the interferon pathway enhances OROV replication, whereas treatment with therapeutic interferon-α suppresses the infection. Molnupiravir, a clinically approved antiviral drug targeting viral RNA-dependent RNA polymerase, markedly inhibits OROV replication and mitigates virus-induced cytopathology. Combining molnupiravir with interferon-α results in synergistic antiviral activity, indicating the complementarity of virus-targeted and host-directed strategies. These findings strengthen preparedness and response to OROV emergence.

Check for updates

This is an open access article under the CC BY license (http://creativecommons.org/licenses/by/4.0/).

INTRODUCTION

Oropouche virus (OROV) is a neglected vector-borne pathogen that causes Oropouche fever, first identified in Latin America and the Caribbean in 1955.[1] In late 2023, it re-emerged in the Brazilian Amazon and quickly spread eastward, establishing transmission across all regions of Brazil by 2024.[2–4] Endemic circulation has also been reported in Barbados, Bolivia, Colombia, Cuba, the Dominican Republic, Ecuador, Guyana, Panama, and Peru.[5,6] Travel-related cases have been reported in Canada, the Cayman Islands, Germany, Italy, Spain, the Netherlands, and the United States.[7–9] Oropouche fever is characterized by usually mild and self-limiting febrile illness. However, severe complications have been reported including neurological manifestations, pregnancy complications, vertical transmission associated with stillbirth and congenital anomalies, and death.[10–14] As of July 2025, no licensed vaccines or antiviral drugs are available to prevent or treat OROV infection.

OROV is a tripartite, negative-sense RNA virus comprising three genome segments, namely, small (S), medium (M), and large (L). It is classified into *Orthobunyavirus oropoucheense* within the *Orthobunyavirus* genus, Peribunyaviridae family.[15] OROV is primarily transmitted by *Culicoides paraensis* midges in an enzootic cycle involving pale-throat sloths (*Bradypus tridactylus*), non-human primates, and likely other wild mammals.[16] In urban settings, a human-amplified transmission cycle may occur through midges.[17] Additionally, mosquito species such as *Culex quinquefasciatus* may contribute to urban transmission, although further studies are needed to confirm their vector competence for OROV.[18]

After transmission through the bite of an infected arthropod, arboviruses enter the bloodstream and initially replicate in organs such as the liver and spleen and then spread to other tissues and organs.[19] Previous studies have shown that OROV replicates efficiently in human hepatic cell lines *in vitro*.[2,20] Similarly, experimental infections in hamster and mouse models revealed high OROV levels in the liver, accompanied by pronounced liver damage. OROV-triggered liver damage and dysfunction were defined by histopathological findings such as hepatocyte necrosis, Kupffer cell hyperplasia, and elevated levels of liver enzymes.[21–25] Clinical evidence also links Oropouche fever to liver dysfunction. For example, jaundice, a condition commonly associated with hepatic impairment, has been documented in several cases.[26,27] In 2024, two fatal cases without underlying health conditions showed elevated liver enzyme levels.[10] Additionally, immunohistochemistry detected abundant OROV antigens in the liver of another fatal case.[28] Despite these observations, how OROV infection affects the human liver remains poorly understood.

In this study, we analyzed liver enzyme levels in patients with OROV infection from the 2023–2024 outbreak and employed primary organoids derived from adult and fetal human livers to investigate OROV infection, cytopathogenesis, and potential antiviral therapeutics.

RESULTS

Liver function tests indicate hepatic involvement in patients with Oropouche fever

To investigate potential liver involvement during OROV infection in humans, we analyzed liver function tests from six imported Oropouche cases in Europe.[8,9,29,30] In this analysis, we assessed the levels of alanine aminotransferase (ALT), a marker for hepatocyte injury; gamma-glutamyl transferase (GGT), a marker for bile duct injury; and aspartate aminotransferase (AST), an enzyme highly expressed in both hepatocytes and cardiac muscle and whose elevation is generally considered a marker of liver injury when interpreted along with ALT.[31] We found that 50% (3 of 6) exhibited elevated levels of both ALT and AST, whereas 1 of 3 patients tested showed elevated GGT levels (Figures S1A–S1C; Table S1).

To further examine hepatic involvement during OROV infection, we evaluated serum levels of ALT, GGT, and AST in 42 patients with Oropouche fever confirmed by quantitative reverse-transcription PCR (RT-qPCR) during the 2024 outbreak in Brazil (Table S2).[2] To minimize confounding factors, patients were matched by age, sex, and the time elapsed between symptom onset and sample collection. We found that 64.9% (24 of 37) of patients exhibited elevated levels of ALT (Figure 1A) and 67.5% (27 of 40) showed elevated levels of AST (Figure 1B). Next, the analysis was stratified by sex groups due to slight disparities in physiological ranges of liver enzymes between males and females.[32] Among female patients, 81.0% (17 of 21) of cases exhibited elevated levels of either ALT or AST, with 76.2% (16 of 21) of cases showing concurrent elevations of both enzymes (Figures 1A and 1B). In male patients, 52.6% (10 of 19) presented higher levels of AST, and 43.8% (7 of 16) showed elevated levels of ALT (Figures 1A and 1B). Elevated GGT levels were observed in 52.3% (11 of 21) of female patients and 28.6% (6 of 21) of male patients (Figure 1C).

Furthermore, we also tested serum levels of alkaline phosphatase (ALP), a marker of liver damage or bone diseases.[33] We found that 15.8% (3 of 19) of males and 20.0% (4 of 20) of females exhibited higher levels of ALP (Figure 1D). Bilirubin levels remained within the physiological range in both sexes (Figures S1D–S1F). Collectively, these findings demonstrate that patients with Oropouche fever exhibited elevated levels of key liver function markers (AST, ALT, GGT, and ALP), suggesting liver damage or dysfunction. Notably, female patients appeared to have a higher incidence of liver enzyme abnormalities compared to male patients (Figure 1E).

OROV productively infects human liver-derived organoids

To investigate whether human livers are susceptible to OROV infection, we utilized primary organoids cultured from adult human liver as an *in vitro* model. These 3D organoids are derived from residential stem cells/progenitors mainly located within the bile duct compartment of human livers and are named intrahepatic cholangiocyte organoids (ICOs).[34,35] These organoids exhibited a polarized, epithelial structure with a single-cell layer and expressed the cholangiocyte marker KRT19, as shown by immunohistochemistry staining (Figure 2A). To assess the

CellPress
OPEN ACCESS

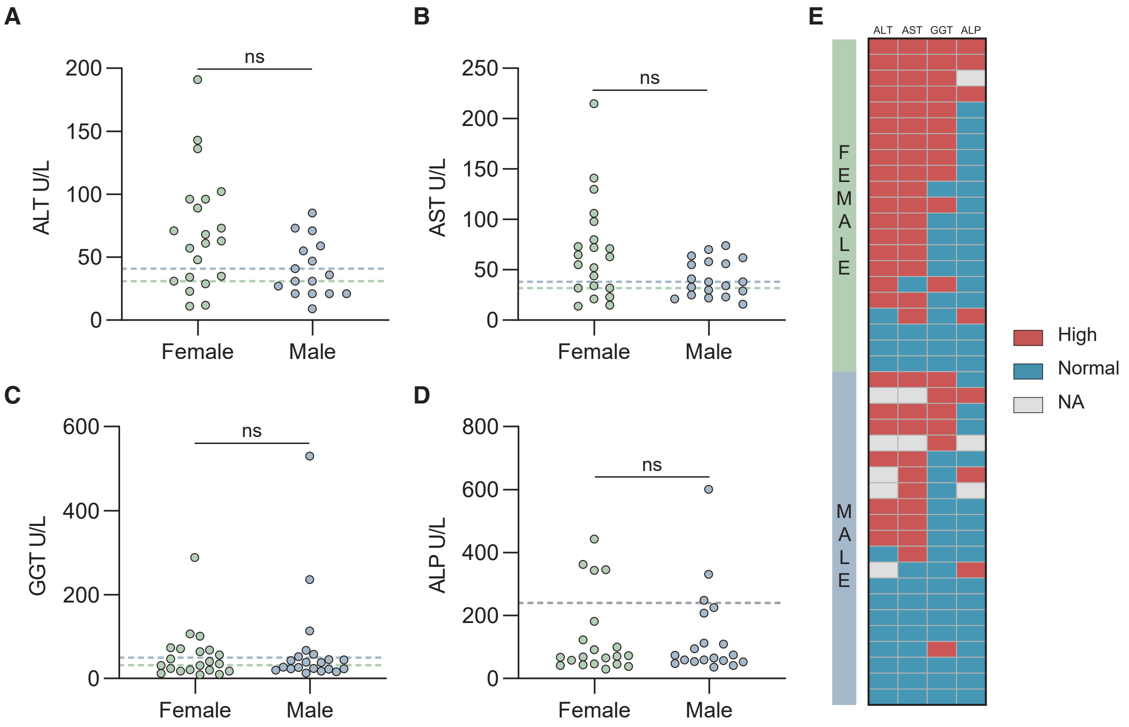

Figure 1. Liver function tests in a cohort of patients with OROV infection

(A) ALT levels in OROV-infected patients recruited from a cohort in Brazil with a threshold (the upper reference limit) of 31 U/L in females (green dotted line) and 41 U/L in males (blue dotted line).

(B) AST levels with a threshold of 32 U/L in females (female, green) and 38 U/L in males (male, blue).

(C) GGT levels with a threshold of 32 U/L in females (female, green) and 50 U/L in males (male, blue).

(D) ALP levels with a threshold of 240 U/L (gray dotted line).

(E) A heatmap depicts elevated or normal liver enzymes in 42 enrolled patients based on the upper reference limit. The threshold references were determined by the kit manufacturers. NA, values not available.

Data represent the values from individual patients; statistical analysis by Mann-Whitney U test; ns, not significant.

susceptibility of ICOs to OROV infection, we utilized two isolates, the historical TRVL9760 strain (OROV-1967) and the IRCCS-SCDC_1/2024 strain (OROV-2024) that represents the reassortant strain associated with the re-emergence of OROV in South America between 2023 and 2024 (Figures 2A, 2B, S2A, and S2B). Amino acid differences in the viral proteins encoded by L, M, and S segments between the two strains were also compared, with RNA-dependent RNA polymerase (RdRp) domain annotations based on the La Crosse virus polymerase structure (PDB 6Z6B) (Figures S2C–S2E).[36]

We first inoculated ICOs cultured from a female donor with both isolates and quantified OROV genomic RNA levels by RT-qPCR and infectious virus titers by 50% tissue culture infectious dose ($TCID_{50}$) assay based on the induction of cytopathic effect (Figure S2F). For the OROV-2024 strain, intracellular virus genome copies showed a continuous increase from 4.6 log_{10} at 1 h post-inoculation to 8.3 log_{10} at 96 h (Figure 2C), while the fold change of RNA levels peaked at nearly 2,000 at 96 h (Figure S2G). Consistently, the titers of OROV infectious particles were undetectable at 1 h in the culture medium and reached 6 log_{10} $TCID_{50}$/mL at 4 days post-inoculation (Figure 2D). Similar results were observed for the OROV-1967 strain (Figures 2C, 2D, and S2G), with OROV infectious titer reaching 6.8 log_{10} $TCID_{50}$/mL at 4 days post-inoculation (Figure 2D). Overall, both strains robustly replicate in organoids, albeit showing subtle differences in the kinetics. We also confirmed the susceptibility of ICOs cultured from a male donor to OROV-1967 infection, by observing an increase of extracellular viral RNA copies into the culture medium (Figure S2H). We verified that both OROV-1967 and OROV-2024 strains replicated efficiently in human hepatoma-derived Huh7 cells, showing time-dependent increases in intracellular RNA levels (Figure S2I).

We further demonstrated viral replication of both OROV isolates by immunostaining the viral surface Gc glycoprotein in infected organoids at 48 h post-inoculation (Figures 2F and S2J), while no Gc signal was detected in the uninfected control group (Figure 2E). Next, our transmission electron microscopy (TEM) analysis of ICOs at 96 h post-OROV infection revealed many extracellular, mature virions near the plasma membrane, along with evidence of virion budding and release (Figures 2G–2I). These virions displayed the characteristic spherical morphology of orthobunyaviruses, with electron-dense cores and surface projections likely corresponding to the glycoprotein spikes. We also observed modified Golgi complexes with swollen sacculi and the presence of immature, annular-shaped OROV particles (Figure 2J). These intracellular and extracellular

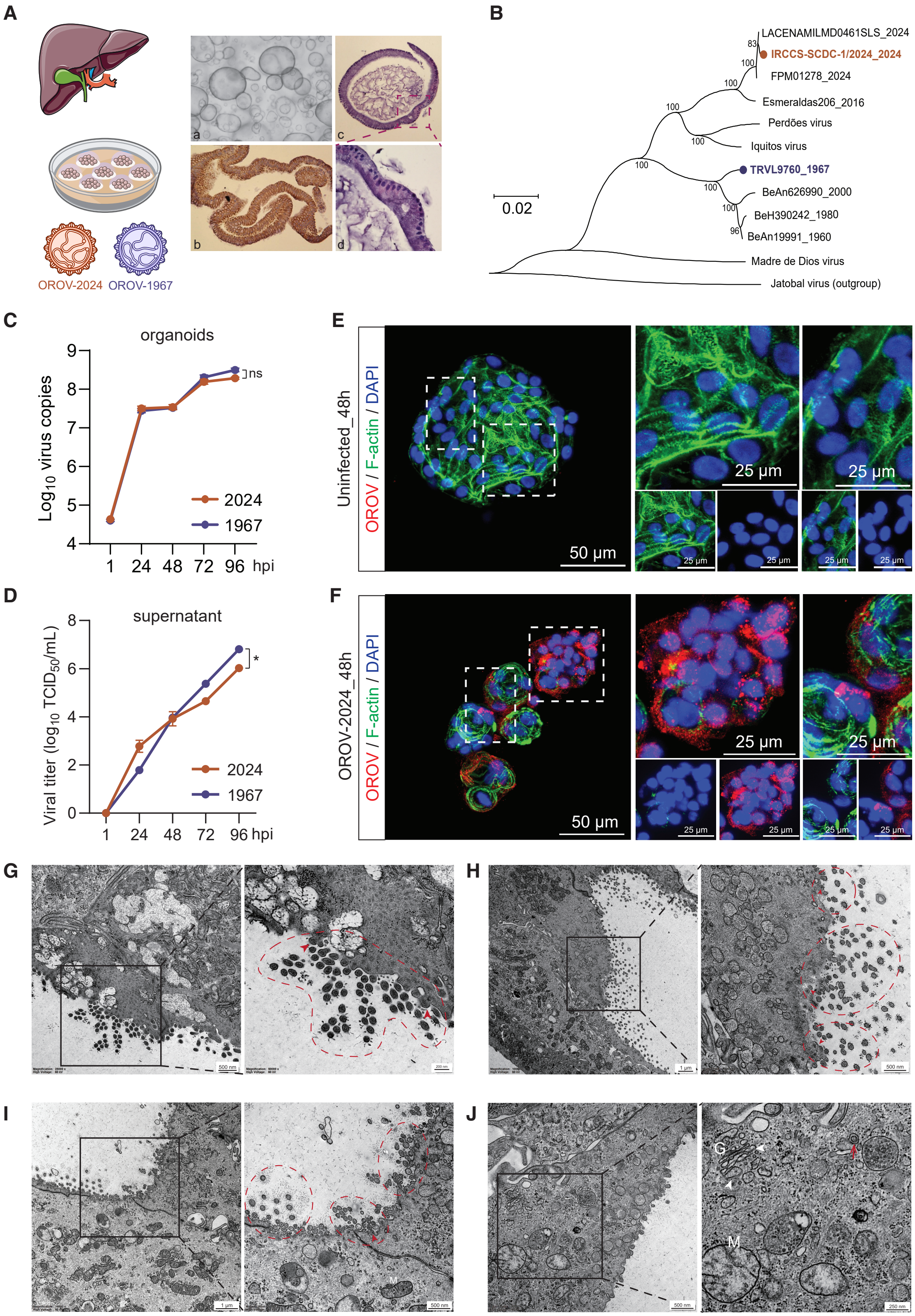

(legend on next page)

CellPress
OPEN ACCESS

viral particles displayed morphological features similar to previously reported description of OROV.[37,38] Notably, some extracellular virions exhibited uneven distribution of surface spikes, which may reflect dynamic glycoprotein organization during viral egress. Altogether, these findings demonstrate that human liver-derived organoids are susceptible to infection by both historical and contemporary OROV strains.

OROV infection triggers a strong antiviral innate immune response in organoids

To investigate virus-host interactions in human liver cells, we inoculated ICOs with the two OROV strains and performed a genome-wide transcriptomic analysis. We set 1-h inoculation as a baseline control, as the eclipse period for OROV infection occurs around 3 h post-exposure.[39] We mapped the host responses at 48 and 96 h post-inoculation when robust viral replication is established. A comparison of the differentially expressed genes among the different time points post-inoculation showed that at least 165 genes were expressed exclusively within each group, while slightly over 10,000 genes were shared among the three groups (Figures 3A and S3A). Volcano plot analysis demonstrated robust host responses to OROV infection at 48 and 96 h compared to 1 h (baseline), with over 5,000 of the genes significantly up- or down-regulated (Figures 3B and S3B). We found that many up-regulated genes are related to innate immune response such as *OAS2*, *IFIT1*, *IFI44L*, and *CXCL11*, which are interferon-stimulated genes (ISGs). These results are consistent for both OROV strains at 48 and 96 h post-inoculation. Furthermore, Gene Ontology analysis revealed that pathways related to response to virus and the interferon signaling pathway were significantly up-regulated, while several host transcriptional and protein targeting pathways were notably down-regulated (Figures 3C and S3C), suggesting the activation of innate antiviral defenses along with a broad suppression of host biosynthetic and metabolic functions. Additionally, gene set enrichment analysis revealed that transcriptional signatures related to viral process, antiviral response, innate immunity, and type I interferon were significantly enriched in organoids at 96 h post-inoculation with both OROV isolates compared to the uninfected controls cultured at the same condition for 96 h (Figures 3D, S3D, and S3E). To further characterize the interferon-mediated antiviral response, we curated a list of ISGs based on a large-scale ISG screen reported in a previous study.[40] We then mapped these genes to our RNA sequencing dataset and selected those that were consistently detected across time points following OROV infection for visualization. To ensure comparability across conditions, the uninfected group was used as a baseline reference at each time point. Heatmap visualization revealed a distinct temporal pattern of ISG expression, with most genes exhibiting low basal levels at 1 h and progressively increasing expression at 48 and 96 h post-inoculation (Figures 3E and S3F). Notably, several representative ISGs including *IFI6*, *IFIT1*, *ISG15*, and *MX1* showed marked upregulation. Overall, these findings demonstrate that OROV replication elicits strong host responses in organoids, particularly activating innate immune pathways.

OROV infection in organoids triggers severe cellular pathogenesis

We next examined whether OROV infection in organoids activates cell death pathways to trigger severe cellular pathogenesis. Gene set enrichment analysis revealed significant upregulation of biological processes related to apoptotic signaling at 96 h post-inoculation compared to uninfected controls (Figure 4A). Depolymerization of the actin cytoskeleton is a hallmark of programmed cell death.[41–43] Immunofluorescence staining of OROV-infected organoids at this time point revealed severe cytopathological changes in Gc-positive cells, including nuclear fragmentation, chromatin condensation, and actin cytoskeleton disassembly (Figures 4B and S4B), consistent with morphologies of severely damaged or late-stage apoptotic cells. Concurrently, we observed extensive extracellular viral signals indicating viral release upon cellular damage. Such patterns were absent in uninfected organoids (Figure S4A).

To investigate the temporal dynamics of OROV infection and cell death, we performed immunostaining at multiple time points from 1 to 96 h post-inoculation. Signals for the OROV Gc protein became detectable by 12 h and increased markedly by 24 h, while staining for the apoptotic marker cleaved caspase-3 remained absent during this early phase (Figure 4C). By 48 and 96 h post-inoculation, Gc protein was broadly distributed throughout the infected organoids, indicating robust viral

Figure 2. Productive infection of OROV in human liver-derived organoids

(A) Schematic representation of adult human liver-derived organoids inoculated with OROV-2024 or OROV-1967. A representative bright-field image shows typical morphology of cultured ICOs (a). Hematoxylin and eosin (H&E) staining of an formalin-fixed paraffin-embedded (FFPE) 4-μm section reveals polarized epithelial architecture with a single-cell-layered structure (b). Immunohistochemistry staining for KRT19 confirms uniform expression of this cholangiocyte marker in organoids (c), with high-magnification detail shown in (d).

(B) Phylogenetic analysis of the OROV L segment highlights the evolutionary divergence between the two strains. A neighbor-joining tree was constructed using the p-distance model, with branch support assessed by 1,000 bootstrap replicates. Only bootstrap values greater than 70% are shown. The tree is midpoint rooted.

(C) Quantification of viral RNA levels in organoids post-inoculation with the two isolates (n = 4 biological replicates).

(D) Quantification of infectious titers in culture medium (0 in x axis represents 1 h post-infection; n = 4 biological replicates).

(E and F) Representative images of uninfected organoids (E) and organoids 48 h post-inoculation with OROV-2024 (F) by immunostaining with the antibody against OROV Gc glycoprotein (red), phalloidin for actin (green), and DAPI for nuclei (blue). Scale bars: 50 and 25 μm.

(G–I) Representative TEM images of organoids 96 h post-inoculation with OROV-2024, showing extracellular mature OROV virions (red frames) and virions being secreted from clathrin-coated vesicles (red arrowheads). Scale bars: 500 nm and 200 nm (magnification) in (G), 1 μm and 500 nm (magnification) in (H and I).

(J) Representative TEM images of organoids 96 h post-inoculation with OROV-2024, showing an immature OROV particle (red arrow) and modified Golgi complexes with swollen sacculi (white arrowheads). Scale bars: 500 nm and 250 nm (magnification). M, mitochondria; G, Golgi complex.

Data represent mean ± SEM.; statistical analysis by mixed-effects model with restricted maximum likelihood (REML); *$p < 0.05$; ns, not significant.

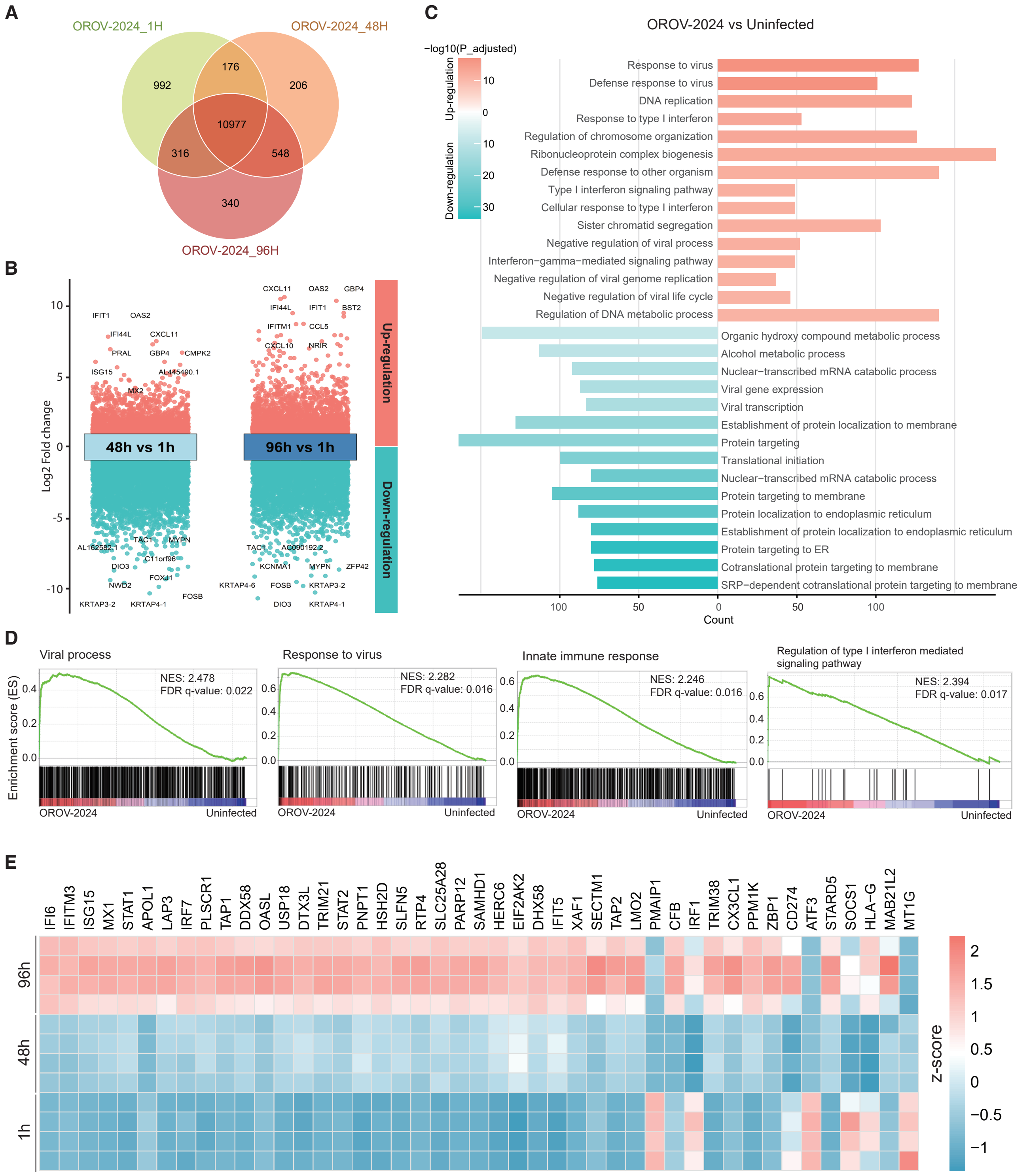

Figure 3. Transcriptomic analyses characterize OROV-host interactions

(A) Venn diagram of overlapped differentially expressed genes in the OROV-2024 strain infected organoids at different time points.

(B) Volcano plot analysis of differentially expressed genes at 48 and 96 h compared to 1 h after OROV-2024 inoculation.

(C) Top 15 up-regulated and top 15 down-regulated pathways identified by Gene Ontology analysis in OROV-2024-inoculated organoids at 96 h post-inoculation compared to the uninfected controls cultured at the same condition for 96 h.

(legend continued on next page)

replication. Interestingly, cleaved caspase-3 signals predominantly localized to the periphery of the organoids and were frequently associated with fragmented nuclei, consistent with apoptotic cell death. Notably, viral and apoptotic markers did not always co-localize, potentially reflecting viral release from disintegrating cells.

Electron microscopy further confirmed organelle damage in infected organoids at 96 h post-inoculation. Infected cells showed swollen mitochondria with disorganized and disintegrating cristae, as well as modified Golgi complexes and extracellular viral particles (Figures 4E–4G). However, organoids with moderate or lower levels of infection retained relatively intact cellular morphology (Figures 4B and S4C–S4F).

Molnupiravir potently inhibits OROV replication

RdRp is responsible for the replication of RNA viruses and is an important antiviral target.[44] To meet the urgent clinical needs, we aimed to identify effective anti-OROV candidates by profiling and repurposing the clinically used RdRp inhibitors (Figure 5A). To this end, we screened a panel of 11 RdRp inhibitors in ICOs infected with the OROV-1967 isolate. To minimize cytotoxic effects on host cells, we used a relatively low concentration (1 μM) and treated for 48 h. Among the tested compounds, only molnupiravir demonstrated effective inhibition of viral replication at this concentration (Figure 5B). Molnupiravir is an orally administered pro-drug of the ribonucleoside analog β-*d*-N4-hydroxycytidine (NHC), which is taken up into cells and phosphorylated to a competitive substrate for viral RdRp, resulting in accumulation of errors in the viral genome and inhibition of viral replication.[45–47]

To map the site-specific binding modes of molnupiravir, NHC, and NHC-5′-triphosphate (Figure S5A), we predicted the RdRp structures (encoded by the L segment) of both strains using AlphaFold3,[48] achieving a high predicted template modeling score (pTM = 0.83), indicating high structural accuracy (Figure S5B). Next, we predicted the potential docking pockets within the RdRp structures (Figures 5C and S5C). Molecular docking analysis revealed that molnupiravir, NHC, and NHC-5′-triphosphate bind to OROV RdRp (Figures S5D–S5J). Consistently, NHC-5′-triphosphate, the active component of molnupiravir and NHC,[49] exhibited a lower Vina score (−7.9) (Figures S5F and S5J), suggesting a more stable and stronger interaction with OROV RdRp.[50] To further support these findings, we performed comparative docking analysis of NHC-5′-triphosphate to the crystal structure of the SARS-CoV-2 RdRp (PDB: 7OZV) (Figure S6A).[51] As expected, ATP and GTP were found to engage the same catalytic residues predicted to interact with NHC-5′-triphosphate (Figures S6B and S6C). Comparative docking of all tested compounds (Figure 5B) revealed that although NHC-5′-triphosphate did not have the strongest predicted binding score, it showed the highest number of predicted interactions with catalytic residues within the NTP-binding pocket. A few other compounds with stronger predicted affinities bound outside the catalytic region did not show antiviral activity in OROV-infected organoids (Figures S7 and 5B). These results indicate that the superior antiviral efficacy of NHC observed in organoids did not directly correlate with docking scores, highlighting the importance of integrating structural predictions with functional validation.

Subsequently, we evaluated a range of concentrations (0.01–50 μM) of molnupiravir and NHC in organoids infected with OROV. Both compounds exhibited dose-dependent inhibition of viral replication of the OROV-2024 isolate, although higher concentrations (e.g., 10–50 μM) moderately affected organoid growth. The half-maximal inhibitory concentration (IC_{50}) values were determined to be 1 μM for molnupiravir and 0.05 μM for NHC (Figures 5D and 5E). Similar results were observed with the OROV-1967 strain, where both compounds demonstrated dose-dependent antiviral activity. The IC_{50} values for molnupiravir and NHC against this strain were 2.2 and 0.05 μM, respectively (Figures S8A and S8B).

Next, we determined the time- and dose-dependent antiviral activity of the active drug NHC in OROV-infected ICOs. Treatment with 0.1 and 1 μM NHC for 48 and 96 h significantly reduced intracellular viral RNA levels ($n = 4$, $p < 0.01$), with no measurable cytotoxicity observed at either concentration (Figure 5F). At 1 μM, RNA levels were reduced by ~90% at 48 h and ~98% at 96 h. Consistently, infectious virus production was also strongly inhibited: 1 μM NHC led to 98.5% and 99.9% reduction in viral titers at 48 and 96 h ($n = 4$, $p < 0.05$), respectively (Figure 5G). To further understand the mode of action of NHC, we performed a time-of-addition assay (Figure 5H).[52] NHC treatment was most effective when administered 2 h post-inoculation with OROV, whereas virucidal treatment, pre-treatment, or treatment during virus inoculation resulted in minimal inhibition (Figure 5I). To assess the effects of prolonged treatment, OROV-infected organoids were continuously exposed to 1 or 5 μM NHC for up to 15 days. Both concentrations maintained robust viral suppression without clear evidence of viral rebound during the treatment period (Figure 5J). Subsequently, we performed an additional 15-day regimen using a stepwise dose-escalation approach, starting at 0.01 μM and gradually increasing to 10 μM. Quantification of viral RNA levels in the supernatant showed progressive inhibition in parallel with the increasing NHC concentrations (Figure S8C).

Molnupiravir treatment partially restores host cellular homeostasis

Immunofluorescence staining showed a clear reduction of viral Gc glycoprotein in NHC-treated organoids, confirming effective inhibition of OROV replication (Figure 6A). NHC treatment also rescued virus-induced cytopathic changes including extensive actin cytoskeleton disruption and nuclear fragmentation. Cleaved-caspase-3 staining further confirmed that NHC treatment mitigated virus-induced apoptotic and lytic cell death at both 48 h and 96 h post-inoculation

(D) Gene set enrichment analysis (GSEA) in OROV-2024-infected organoids at 96 h post-inoculation compared to uninfected controls.
(E) Heatmap showing the expression of selected ISGs in OROV-2024-infected organoids at 1, 48, and 96 h post-inoculation. ISGs were sourced from a published large-scale ISG screen[40] and mapped to our RNA-seq dataset. Data are representative of four biological replicates from one RNA-seq experiment using two independent OROV isolates with multiple time points. The color scale represents row-wise *Z* score of gene expression across samples.

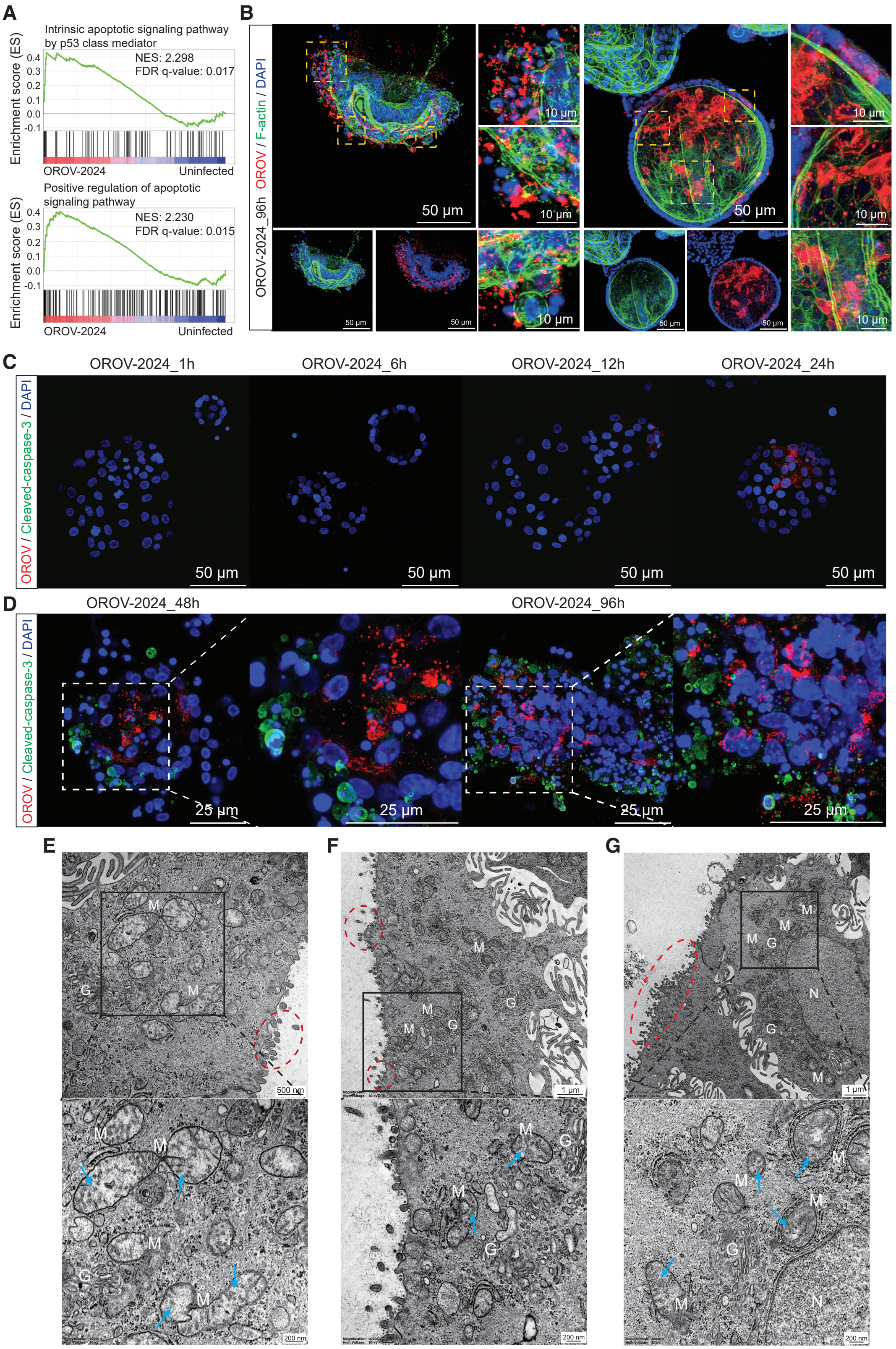

(legend on next page)

(Figures 6B, S8C, and S8D). Lactate dehydrogenase (LDH) release assays, which measure membrane damage associated with cell death, showed a similar trend, with NHC treatment significantly lowering virus-induced cytotoxicity at both 48 and 96 h (Figure 6C). Transcriptomic analysis showed that NHC treatment for 96 h substantially alleviated rewiring of the host transcriptome by OROV infection and reversed the activation of viral response and apoptotic signaling pathways, while restoring the balance in host immune responses (Figures 6D, 6E, and S8E). Notably, OROV-mediated activation of multiple ISGs was attenuated upon treatment (Figure 6F), suggesting restoration of host cellular homeostasis. We also found that OROV infection disturbed the transcription of mitochondrial-related genes, including upregulation of genes involved in apoptosis and mitophagy, as well as dysregulation of genes essential for mitochondrial respiration and morphology dynamics. These abnormalities were largely restored by NHC treatment (Figure S8F). Collectively, these findings provide preclinical evidence supporting the potential of molnupiravir as an antiviral therapeutic candidate for treating OROV infection.

Human fetal liver-derived organoids are susceptible to OROV infection but potently inhibited by molnupiravir

Given that OROV RNA was detected in liver tissue of a stillborn infant caused by vertical transmission during the 2023–2024 outbreak,[53] we assessed viral susceptibility of primary organoids cultured from human fetal liver. These organoids, which also carry a cholangiocyte phenotype,[54] were then inoculated with the OROV-2024 isolate (Figure S9A). Intracellular and extracellular viral genome copy numbers increased by 7.7 $\log_{10}$ and 4 $\log_{10}$ at 3 days compared to 1 h post-OROV infection, respectively (Figures S9B and S9C). Subsequently, we evaluated the antiviral activity of NHC, the active form of molnupiravir, in OROV-infected fetal organoids. Treatment with 1 μM NHC for 72 h resulted in 80% inhibition of intracellular ($p < 0.05$), with no measurable cytotoxicity observed (Figure S9D), and a 96.9% reduction in extracellular viral titers in the culture supernatant ($p < 0.05$) (Figure S9E). Consistently, immunofluorescence staining showed substantial reduction of Gc glycoprotein in NHC-treated organoids compared to the untreated group, confirming the potent antiviral effect of NHC against OROV infection (Figures S9F–S9H). These data demonstrate that human fetal liver-derived organoids are susceptible to OROV infection, which can be effectively inhibited by NHC treatment.

Hepatocyte-differentiated organoids support OROV replication and respond to molnupiravir treatment

To further confirm our findings in a liver-relevant model, we differentiated ICOs into hepatocyte-like organoids using a well-defined protocol (Figure S10A).[35,54] After a 15-day process of differentiation, the resulting organoids showed increased expression levels of the hepatocyte markers albumin and CYP3A4, while the expression level of the progenitor cell marker LGR5 decreased, acquiring hepatocyte morphology of polygonal cell shapes (Figure S10B). Hepatocyte-differentiated organoids supported robust replication of the OROV-2024 strain, with viral RNA copy numbers increasing from 5.8 $\log_{10}$ at 1 h post-inoculation to 7.5 $\log_{10}$ at 72 h in organoids and from 2.7 $\log_{10}$ at 1 h post-inoculation to 6.1 $\log_{10}$ at 72 h in culture supernatants (Figures S10C and S10D). Treatment with 1 μM NHC significantly inhibited intracellular viral RNA levels by 80.4% without affecting cell viability and reduced infectious virus titers in the supernatant by 81.1% (Figures S10E and S10F). NHC treatment also reduced both OROV Gc protein and cleaved-caspase-3 signals in infected hepatocyte-differentiated organoids and attenuated LDH release (Figures S10G and S10H). These findings demonstrate that OROV can productively infect and replicate in hepatocyte-differentiated organoids and further confirm the efficacy of molnupiravir in inhibiting viral replication and virus-induced cell damage.

Harnessing host antiviral interferon response to develop molnupiravir-based combination therapy

Given the general concern that direct-acting antivirals such as molnupiravir may induce drug-resistance mutations,[55] we analyzed the genomic sequences of OROV following serial passaging in Vero cells with or without escalating concentrations of NHC treatment (Figure 7A). By Nanopore-based whole-genome sequencing, one mutation was found in the consensus sequence of one treated sample (T5498C, segment L, nonsynomynous) and one mutation was found in the consensus sequence of all three treated samples (C66T, segment S, nonsynomynous) (Figure 7B). However, both mutations were present already as minor variant in either the input or in the control passaged viruses (Table S3). Furthermore, given that both are nonsynomynous mutations, there are no clear functional implications. In addition, a deletion of three nucleotides (N621Δ, segment M) was found in all cultured viruses which might be a culture-based adaption since it was not found in the input virus stock.

Figure 4. Cellular pathogenesis induced by OROV infection in organoids

(A) GSEA analysis shows the activation of apoptotic signaling pathways in OROV-infected organoids at 96 h post-inoculation compared to the uninfected controls cultured at the same condition for 96 h. Data are representative of four biological replicates from one RNA-seq experiment.

(B) Representative images of organoids 96 h post-inoculation with the OROV-2024 by immunostaining with the antibody against OROV Gc glycoprotein (red), phalloidin for F-actin (green), and DAPI for nuclei (blue). Scale bars, 50 and 10 μm.

(C) Representative images of organoids at 1, 6, 12, and 24 h post-inoculation with OROV-2024 by immunostaining with the antibodies against OROV Gc protein (red), cleaved-caspase-3 (green), and DAPI (blue). Scale bars, 50 μm.

(D) Representative images of organoids at 48 and 96 h post-inoculation with OROV-2024 by immunostaining with the antibodies against OROV Gc protein (red), cleaved-caspase-3 (green), and DAPI (blue). Scale bars, 25 μm.

(E–G) Transmission electron microscopy images of OROV-infected organoids at 96 h post-inoculation, showing damaged mitochondria (M) with disorganized and disintegrating cristae (blue arrows), modified Golgi complexes (G), and extracellular viral particles (red circles). Insets show magnified views. Scale bars: 500 nm and 200 nm (E) and 1 μm and 200 nm (F and G). N, nucleus.

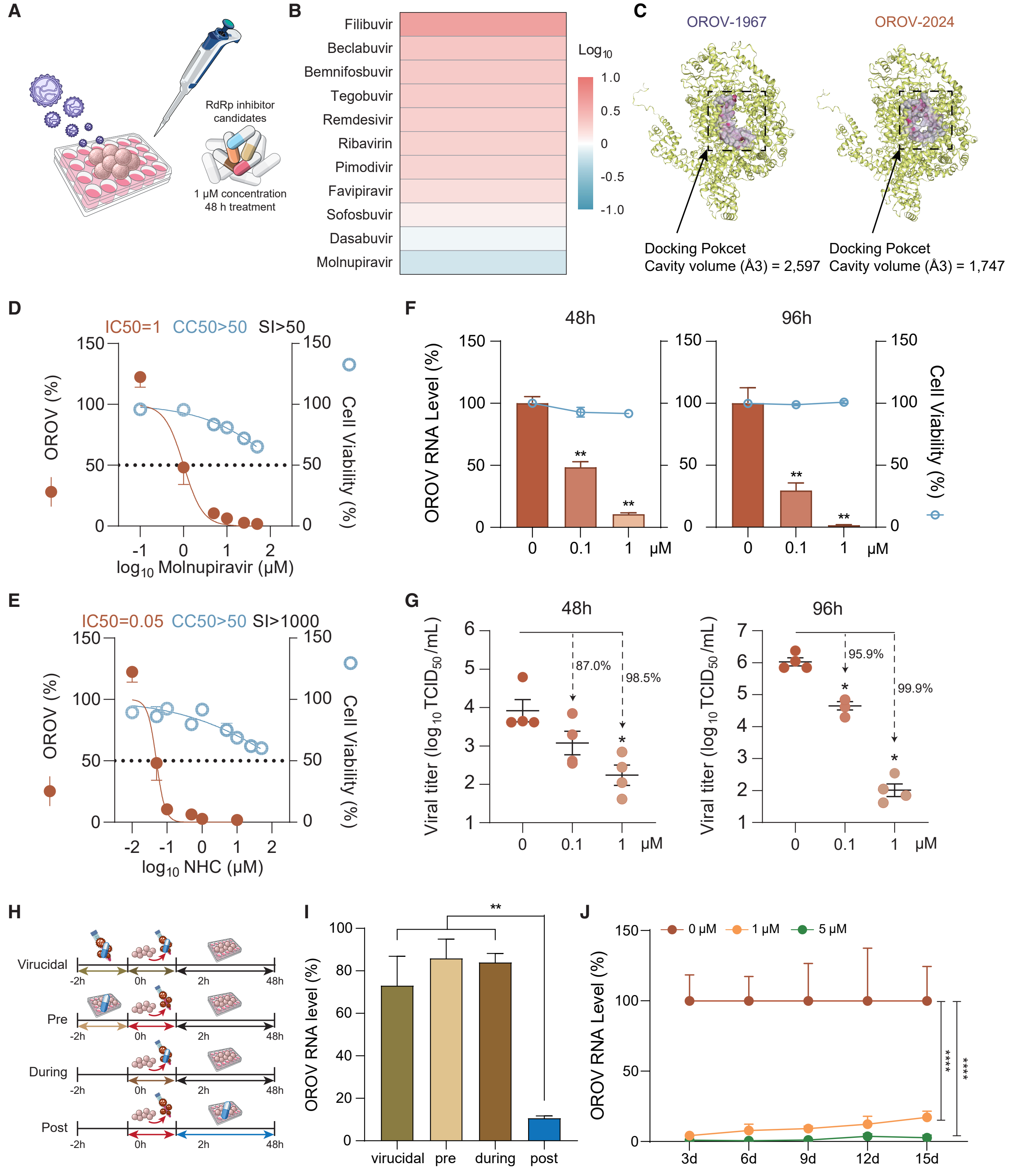

Figure 5. Antiviral drug profiling identified molnupiravir as a potent inhibitor of OROV infection

(A) Schematic diagram of profiling RdRp inhibitors, prioritizing clinically used drugs, in OROV-infected liver organoids.

(B) Fold changes of OROV RNA levels in infected organoids after 48-h treatment with the selected RdRp inhibitors.

(C–E) (C) Potential docking pocket of RdRp protein predicted by CurPocket. RNA-protein contact active site cavity is indicated as a dotted circle (D and E). Dose-response curves of molnupiravir (D) and NHC (E) in infected organoids, showing the 50% inhibitory concentration (IC_{50}) and 50% cytotoxic concentration (CC_{50}) values (*n* = 4 biological replicates).

(legend continued on next page)

Although our findings suggest a relatively high barrier to resistance development, combining antivirals with complementary mechanisms remains a common clinical strategy to enhance efficacy and reduce the risk of drug resistance. Transcriptomic analysis revealed robust activation of interferon responses, indicated by the induction of a panel of ISGs, in organoids infected with OROV for 96 h (Figure 7C). Pharmacological inhibition of the interferon pathway using a JAK inhibitor markedly increased viral RNA levels and infectious viral titers (Figures 7D and 7E) while suppressing ISG expression (Figure 7F). These findings demonstrate that the endogenous interferon response plays a functional role in restricting OROV replication. Conversely, treatment with recombinant interferon-alpha (IFN-α), a clinically approved therapy for several viral infections,[56] dose-dependently inhibited intracellular viral RNA level (Figure 7G) and infectious virus production (Figure 7H), accompanied by ISG induction (Figure 7I).

Finally, to leverage complementary antiviral mechanisms, we tested the combination of NHC and IFN-α in OROV-infected organoids. A range of concentrations was applied individually or in combination, followed by viral RNA quantification and synergy modeling using the SynergyFinder 3.0 tool.[57] The analysis revealed a strong synergistic antiviral effect, indicated by a high synergy score (mean = 21.16) (Figures 7J and 7K). These findings support a combination strategy that integrates virus-targeting and host-directed antiviral approaches to achieve more effective control of OROV infection.

DISCUSSION

Previous experimental studies in murine models demonstrated that OROV infection causes significant liver damage, especially targeting hepatocytes.[21–25] Our study provides clear evidence that OROV infection can induce hepatic alterations in patients with Oropouche fever. Elevated liver transaminases (ALT, AST, and GGT) observed in some patients point to possible injury of both hepatocyte and cholangiocyte compartments.[31] We hypothesize that OROV's liver tropism may be linked to high expression of low-density lipoprotein receptor-related protein 1 (LRP1) in liver cells,[58,59] which has been proposed as an OROV entry factor.[60] Notably, similar mechanisms of LRP1-dependent liver tropism have been observed in Rift Valley fever virus, a related tri-segmented RNA virus.[61,62]

We demonstrated that human liver organoids derived from both fetal and adult livers support productive OROV infection. The cholangiocyte phenotype of these organoids corresponds with elevated GGT levels observed in some patient cases. In addition to cholangiocyte-like ICOs, we showed that hepatocyte-differentiated organoids[35] are also permissive to OROV and exhibit virus-induced cytopathic effects. Following infection, viral loads progressively increased, accompanied by clear signs of cell injury, including cytoskeletal disassembly, membrane damage, LDH release, and cleaved caspase-3, consistent with a lytic infection cycle. While these cellular responses represent injuries at the epithelial cell level rather than organ pathology, clinical observations have also shown that liver involvement can occur during OROV infection. A recent autopsy report described abundant viral antigens in the liver together with severe hepatic injury.[28] In addition, transcriptomic profiling revealed activation of apoptotic pathways and engagement of host antiviral responses, reflecting a strong inflammatory state that likely acts together with direct viral damage to drive cellular- and tissue-level injury. These findings point to the need for targeted therapeutic strategies to mitigate the OROV-induced severe consequences.

We discovered that molnupiravir potently inhibits OROV infection in human liver-derived organoids. Originally developed for influenza, molnupiravir was later repurposed for COVID-19 treatment in several countries.[47,63] Previous studies have shown its broad-spectrum antiviral activity against SARS-CoV-2[64] and seasonal coronaviruses.[52] As a prodrug, molnupiravir is metabolized into its active form, NHC, which inhibits viral RNA replication.[65] Its antiviral mechanism involves incorporation of NHC ribonucleoside into viral RNA by RdRp, leading to mispairing with cytidine or uridine, inducing transition mutations and suppressing viral replication.[47,51] Consistent with this mode of action, our data showed that molnupiravir treatment after OROV inoculation significantly reduced viral replication in liver organoids, with even greater inhibition observed using the active NHC metabolite. The time-of-addition analysis[52] revealed that NHC was most effective when administered post-infection, whereas pre-treatment, co-treatment during inoculation, or direct incubation with virus particles (virucidal) had limited effects. These findings support its role as an RdRp inhibitor targeting OROV replication. Notably, molnupiravir/NHC treatment also alleviated OROV-induced cytopathology and apoptotic cell death.

One potential concern with molnupiravir is its ability to introduce mutations into viral RNA, which could increase the risk of drug resistance, especially during prolonged treatment. However, since Oropouche fever is typically an acute, self-limiting illness with symptoms lasting 2–7 days,[12] antiviral therapy would likely be short term, prescribed for only a few days. In our liver organoid models, both short-term (2–4 days) and extended

(F) Intracellular OROV RNA levels (n = 5 biological replicates) and corresponding cell viability (n = 8 biological replicates) following 48- and 96-h treatment with NHC (0.1 or 1 μM).
(G) Infectious viral titers in culture supernatant from organoids treated with NHC at 48 and 96 h (n = 4 biological replicates).
(H) Schematic of the time-of-addition experiment to assess the timing of NHC-mediated antiviral activity.
(I) OROV RNA levels under virucidal treatment (virucidal, n = 7 biological replicates), pre-treatment (pre, n = 6 biological replicates), treatment during virus inoculation (during, n = 7 biological replicates), and post-treatment conditions (post, n = 5 biological replicates). Data are shown as relative levels compared to matched untreated controls in each group. The post-treatment group is same as the condition of 48-h treatment with 1 μM concentration in (F).
(J) OROV RNA levels in the supernatant of cultured organoids without or with NHC treatment (1 or 5 μM) over a 15-day period. Data are shown as relative levels normalized to untreated controls (n = 5 biological replicates).
Statistical comparisons between treatment groups were performed using two-tailed Mann-Whitney U tests (F, G, and I). Statistical analysis for longitudinal data were performed using mixed-effects model (REML) (J). Data represent mean ± SEM; *$p < 0.05$, **$p < 0.01$, ****$p < 0.0001$.

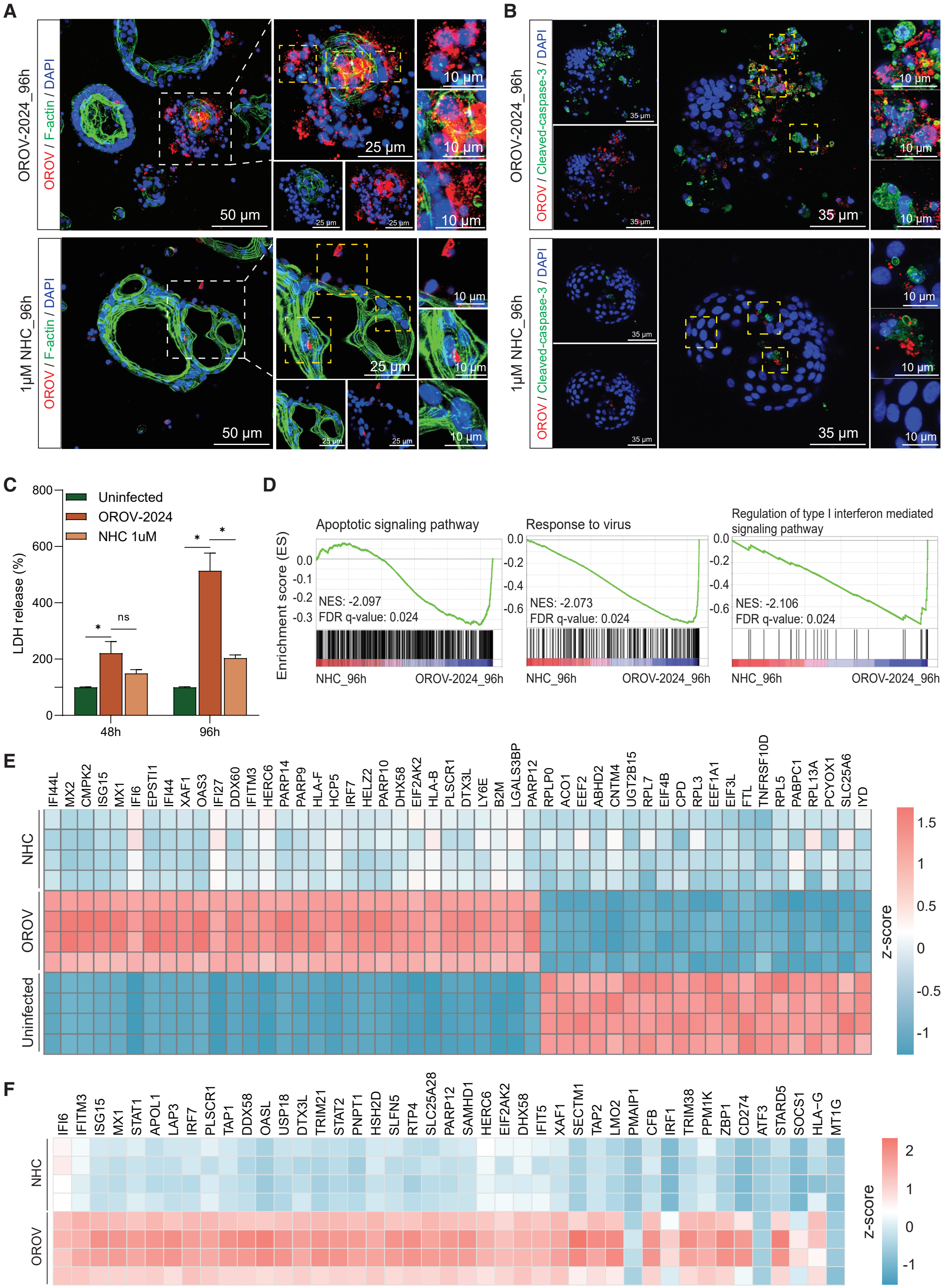

(legend on next page)

CellPress
OPEN ACCESS

(15-day) treatment with molnupiravir effectively suppressed OROV replication without clear evidence of resistance. Additionally, Nanopore sequencing of the complete OROV genome after continuous passaging under NHC pressure in Vero cells identified two mutations. However, both were nonsynonymous without clear functional implications, suggesting no evidence for the emergence of drug-resistant variants in this experimental setup. Nevertheless, the potential risk of resistance emergence should be thoroughly assessed in future studies. Lessons from real-world use of molnupiravir for COVID-19, even with standard 5-day regimen, show that a distinct drug-associated mutational signature has appeared in global SARS-CoV-2 genomes.[55] Therefore, if molnupiravir advances to clinical use for treating Oropouche, careful monitoring of viral evolution in treated individuals as well as in the broader population will be essential to detect and prevent the emergence of resistant variants.

In clinical settings, the maximum serum concentration of NHC, the active metabolite of molnupiravir, can reach approximately 8,000 ng/mL (~30 μM).[65] In our study, NHC inhibited OROV replication with an IC_{50} value as low as 0.05 μM, and we confirmed potent antiviral activity at concentrations of 0.1, 1, and 5 μM. Consistent with a previous study,[66] we also demonstrated that ribavirin, a commonly used antiviral, lacks efficacy against OROV. In addition to direct-acting antivirals, we also evaluated host-directed strategies based on the interferon response. While pre-treatment with IFN-α has shown some anti-OROV activity in mouse models, post-infection administration was not effective.[67] In human liver-derived organoids, we found that OROV infection induced robust interferon responses which functionally restrict viral replication. Importantly, treatment with IFN-α significantly inhibited OROV infection and the combination of molnupiravir with IFN-α resulted in strong synergistic antiviral activity. These findings highlight the strong potential of repurposing molnupiravir, and its combination with host-directed antivirals such as IFN-α, for OROV treatment. However, our antiviral evidence is primarily based on organoid-based experiments. Further *in vivo* studies are recommended to confirm the definitive therapeutic efficacy and pharmacodynamics of these agents. Clinical safety data on molnupiravir use during pregnancy remain unavailable,[47] and potential risks should be carefully evaluated before clinical application.

In summary, our study showed the liver as a key target organ during OROV infection in humans. We demonstrated that primary organoids derived from adult and fetal human livers are highly permissive to OROV, leading to severe cellular pathology and establishing a robust, physiologically relevant *in vitro* model for OROV research. OROV infection elicits a strong antiviral interferon response, and treatment with IFN-α effectively inhibits viral replication. Finally, we identified molnupiravir as a promising antiviral candidate against OROV, and its combination with IFN-α exerts strong synergistic effects. These findings provide a framework for further *in vivo* studies to enable targeted interventions and rapid responses to this emerging public health threat.

Limitations of the study

This study has several limitations. First, available liver function data were limited to patients with mild OROV infections and lacked information on pre-existing liver conditions. Liver biopsies from patients with severe or fatal cases are needed for a comprehensive understanding of OROV-associated liver pathology. Second, while we used both historical and recent viral strains, the study was not designed to compare them. A rigorous comparison would require a larger panel of strains and a dedicated study design. Third, our liver organoids consist solely of epithelial cells and lack blood flow and multicellular tissue architecture; therefore, they model cellular injury rather than organ-level pathology. Incorporating immune components in future models will be essential to elucidate the immunopathogenesis of OROV-induced liver injury. Additionally, although organoids from both male and female donors were used, the study was not specifically designed to analyze sex-specific differences in infection and treatment responses. Fourth, while our *in vitro* data demonstrate strong antiviral activity of molnupiravir against OROV, *in vivo* studies are necessary to validate its efficacy. Finally, the precise mechanism by which molnupiravir inhibits OROV replication remains to be fully elucidated.

RESOURCE AVAILABILITY

Lead contact

Further information and requests for resources and reagents should be directed to and will be fulfilled by the lead contact, Qiuwei Pan (q.pan@erasmusmc.nl).

Materials availability

No novel, unique reagents were developed as part of this research.

Figure 6. Molnupiravir attenuated virus-induced cell death and modulates host antiviral responses in OROV-infected liver organoids

(A) Representative images of OROV-2024-infected organoids without (top) or with (bottom) 96-h NHC (1 μM) treatment by immunostaining with the antibody against OROV Gc glycoprotein (red), phalloidin for F-actin (green), and DAPI for nuclei (blue). Scale bars: 50 , 25, and 10 μm.

(B) Representative images of OROV-2024 infected organoids without (top) or with (bottom) 96-h NHC (1 μM) treatment by immunostaining with the antibody against OROV Gc glycoprotein (red), cleaved-caspase-3 (green), and nuclei (DAPI, blue). Scale bars: 35 and 10 μm.

(C) LDH release in culture supernatant of organoids at 48 and 96 h post-inoculation, comparing uninfected, OROV-infected, and NHC-treated groups (1 μM) (n = 4 biological replicates).

(D) GSEA in OROV-infected organoids with NHC treatment for 96 h compared to organoids with OROV infection alone.

(E) Heatmap of the top 50 significantly regulated genes comparing uninfected controls, OROV-infected organoids at 96 h post-inoculation, and OROV-infected organoids with 96-h NHC treatment.

(F) Heatmap of selected ISGs comparing OROV-infected organoids at 96 h post-inoculation and OROV-infected organoids with 96-h NHC treatment. ISGs were sourced from a published large-scale ISG screen[40] and mapped to our RNA-seq dataset. The color scale represents row-wise Z score of gene expression across samples.

Data are representative of four biological replicates from one RNA-seq experiment (D–F). Data represent mean ± SEM; statistical analysis by Mann-Whitney U test; $^{*}p < 0.05$; ns, not significant.

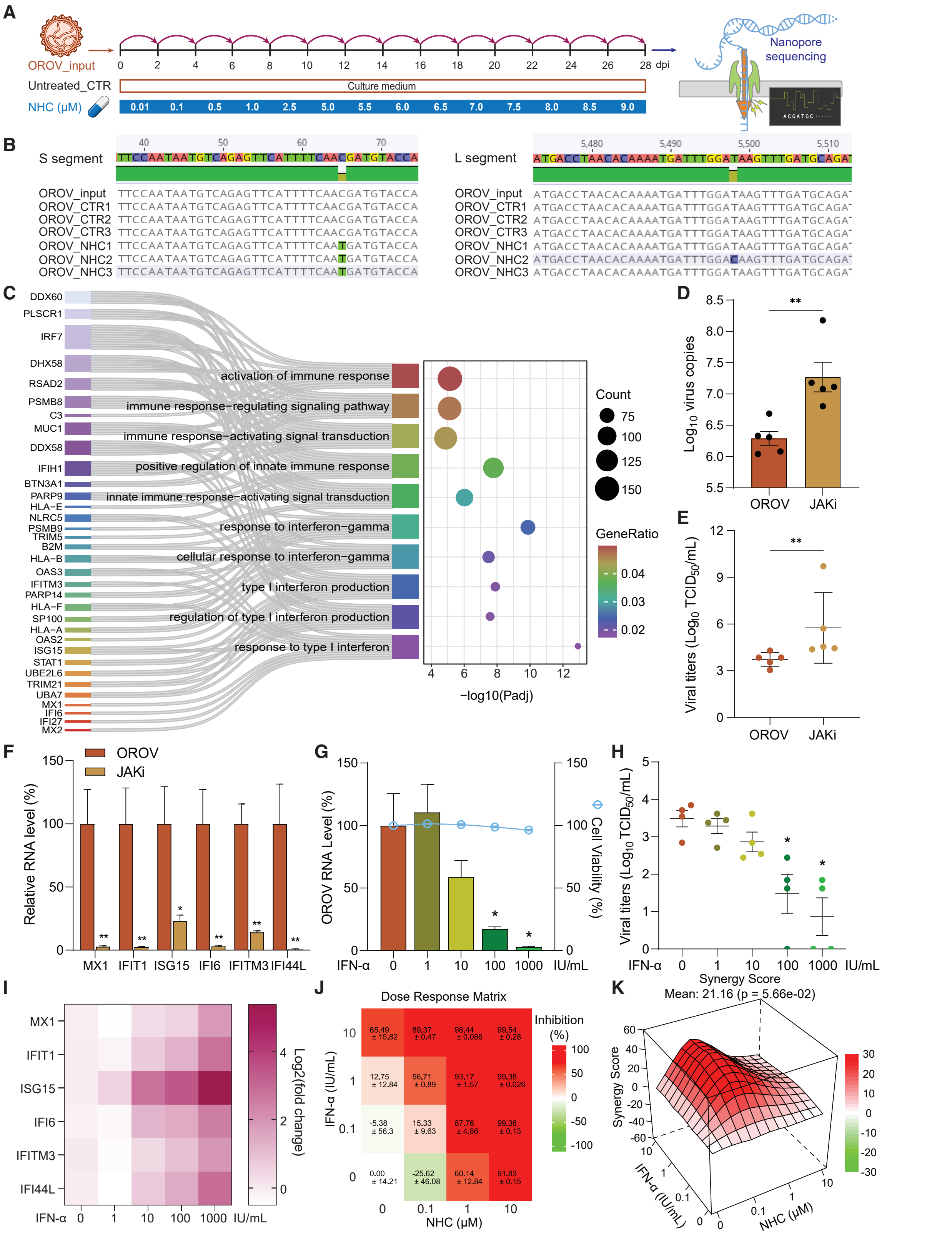

(legend on next page)

CellPress
OPEN ACCESS

Data and code availability

- Data: RNA sequencing data have been deposited to in the National Center for Biotechnology Information (NCBI) Gene Expression Omnibus (GEO) database (GEO: GSE316780). The accession number is also listed in the key resources table.
- Code: This paper does not report original code.
- Other items: Any additional information required to reanalyze the data reported in this work paper is available from the lead contact (Qiuwei Pan, q.pan@erasmusmc.nl) upon request.

ACKNOWLEDGMENTS

Q.P. is supported by a VIDI personal grant (no. 91719300) from the Netherlands Organisation for Health Research and Development (ZonMw). W.M.d.S. is supported by Burroughs Wellcome Fund—Climate Change and Human Health Seed Grants (grant number 1022448) and a Wellcome Trust—Digital Technology Development Award in Climate Sensitive Infectious Disease Modeling (grant number 226075/Z/22/Z). J.L.P.-M. is supported by the São Paulo Research Foundation (grant number 2022/10442-0) and National Council for Scientific and Technological Development (grant number 309971/2023-3). We are grateful to the staff of the CASA clinics in Leiden and Rotterdam for collecting fetal tissues. We thank Dr. Ron Smits from the Department of Gastroenterology and Hepatology, Erasmus MC, the Netherlands, for providing technical assistance. We thank Dr. Sven Reiche from Federal Research Institute for Animal Health, Germany, for providing the anti-OROV Gc antibody and Dr. Chantal Reusken from the National Institute for Public Health and the Environment, the Netherlands, for providing the OROV strain TRVL9760.

AUTHOR CONTRIBUTIONS

J.L. and X.W., conceptualization, methodology, formal analysis, investigation, data curation, writing – original draft, writing – review & editing, and visualization; Y.D. and F.Q., methodology, formal analysis, investigation, data curation, writing – original draft, writing – review & editing, and visualization; S.T.S.d.L., methodology, formal analysis, investigation, data curation, writing – review & editing, and visualization; L.P., methodology, formal analysis, investigation, and writing – review and editing; R.S., M.M.A.V., and M.B., methodology and investigation; J.F., I.M.C., X.H., and L.M.S.M., investigation and formal analysis; D.M.O., M.P.P., A.B., E.P., H.L.A.J., J.A.T.S., M.N.N.d.S., E.C.P., J.L.P.-M., P.L., A.A.A., L.J.W.v.d.L., and C.C., investigation; B.B.O.M., methodology, formal analysis, investigation, and data curation; W.M.d.S. and W.W., conceptualization, methodology, resources, writing – review & editing, and supervision; Q.P., conceptualization, resources, writing – original draft, writing–review & editing, supervision, project administration, and funding acquisition.

DECLARATION OF INTERESTS

The authors declare no competing interests.

STAR★METHODS

Detailed methods are provided in the online version of this paper and include the following:

- KEY RESOURCES TABLE
- EXPERIMENTAL MODELS AND STUDY PARTICIPANT DETAILS
  - Infection diagnosis and liver function tests in human participants
  - Culture of human liver-derived organoids
  - Cell culture
- METHOD DETAILS
  - Immunohistochemistry
  - Hepatocyte differentiation of organoids
  - OROV strains and viral inoculation
  - Antiviral compound treatment and IC_{50}/CC_{50} analysis
  - Phylogenic analysis
  - Molecular docking
  - RNA extraction and RT-qPCR detection
  - Quantification of virus genome copy numbers
  - $TCID_{50}$ assay
  - Immunofluorescence assay
  - LDH release assay
  - AlamarBlue assay
  - Genome-wide RNA sequencing and data analysis
  - Passaging OROV under NHC pressure and nanopore sequencing
- QUANTIFICATION AND STATISTICAL ANALYSIS

SUPPLEMENTAL INFORMATION

Supplemental information can be found online at https://doi.org/10.1016/j.xcrm.2026.102646.

Received: February 15, 2025
Revised: July 23, 2025
Accepted: February 2, 2026
Published: March 9, 2026

REFERENCES

1. Anderson, C.R., Spence, L., Downs, W.G., and Aitken, T.H. (1961). Oropouche virus: a new human disease agent from Trinidad, West Indies. Am. J. Trop. Med. Hyg. *10*, 574–578.
2. Scachetti, G.C., Forato, J., Claro, I.M., Hua, X., Salgado, B.B., Vieira, A., Simeoni, C.L., Barbosa, A.R.C., Rosa, I.L., de Souza, G.F., et al. (2024). Re-emergence of Oropouche virus between 2023 and 2024 in Brazil: an observational epidemiological study. Lancet Infect. Dis. *25*, 166.

Figure 7. Harnessing host interferon response for developing molnupiravir-based antiviral combination
(A) Schematic illustration of serial passaging with ($n = 3$ biological replicates) or without ($n = 3$ biological replicates from one passaging experiment series) NHC treatment of escalating concentrations in Vero cells.
(B) Identification of mutations by whole-genome sequencing of OROV using Nanopore sequencing technology.
(C) Sankey-bubble plot showing significantly enriched immune and interferon-related pathways in OROV-infected human adult liver-derived organoids at 96 h post-inoculation. Data are representative of four biological replicates from one RNA-seq experiment.
(D) Quantification of OROV RNA levels in organoids with or without JAK inhibitor treatment ($n = 5$ biological replicates).
(E) Infectious viral titers in culture medium from organoids treated with or without JAK inhibitor ($n = 5$ biological replicates).
(F) Relative expression of selected ISGs in OROV-infected organoids treated with or without 10 μM JAK inhibitor ($n = 4$ biological replicates).
(G) Intracellular OROV RNA levels and corresponding cell viability following IFN-α treatment for 48 h with increasing concentrations (0, 1, 10, 100, 1,000 IU/mL) ($n = 4$ biological replicates).
(H) Infectious viral titers in culture medium following IFN-α treatment ($n = 4$ biological replicates).
(I) Heatmap showing induced expression of representative ISGs after 48-h treatment of IFN-α at different concentrations. Values represent $\log_2$(fold change) relative to untreated controls.
(J and K) Dose-response matrix (J) and ZIP synergy landscape (K) of IFN-α and NHC combination treatment in OROV-infected organoids, generated by SynergyFinder 3.0.
Data represent mean ± SEM; statistical analysis by two-tailed Mann-Whitney U test unless otherwise indicated; *$p < 0.05$, **$p < 0.01$.

3. Moreira, F.R.R., Dutra, J.V.R., de Carvalho, A.H.B., Reis, C.R., Rios, J.S.H., Ribeiro, M.d.O., Arruda, M.B., Alvarez, P., Souza, R.P., Voloch, C., et al. (2024). Oropouche virus genomic surveillance in Brazil. Lancet Infect. Dis. *24*, e664–e666.

4. Naveca, F.G., Almeida, T.A.P.d., Souza, V., Nascimento, V., Silva, D., Nascimento, F., Mejía, M., Oliveira, Y.S.d., Rocha, L., Xavier, N., et al. (2024). Human outbreaks of a novel reassortant Oropouche virus in the Brazilian Amazon region. Nat. Med. *30*, 3509–3521.

5. Moutinho, S. (2024). Little-known virus is on the rise in South America. Science *384*, 1052–1053.

6. Quaglia, S. (2024). Clues emerge about an obscure virus' sudden spread. Science *386*, 256–257.

7. Morrison, A., White, J.L., Hughes, H.R., Guagliardo, S.A.J., Velez, J.O., Fitzpatrick, K.A., Davis, E.H., Stanek, D., Kopp, E., Dumoulin, P., et al. (2024). Oropouche Virus Disease Among U.S. Travelers - United States, 2024. MMWR Morb. Mortal. Wkly. Rep. *73*, 769–773.

8. Castilletti, C., Huits, R., Mantovani, R.P., Accordini, S., Alladio, F., and Gobbi, F. (2024). Replication-Competent Oropouche Virus in Semen of Traveler Returning to Italy from Cuba, 2024. Emerg. Infect. Dis. *30*, 2684–2686.

9. Igloi, Z., Soochit, W., Munnink, B.B.O., Anas, A.A., von Eije, K.J., van der Linden, A., Mandigers, M., Wijnans, K., Voermans, J., Chandler, F.D., et al. (2025). Oropouche Virus Genome in Semen and Other Body Fluids from Traveler. Emerg. Infect. Dis. *31*, 205–206.

10. Bandeira, A.C., Pereira, F.M., Leal, A., Santos, S.P.O., Barbosa, A.C., Souza, M.S.P.L., de Souza, D.R., Guimaraes, N., Fonseca, V., Giovanetti, M., et al. (2024). Fatal Oropouche Virus Infections in Nonendemic Region, Brazil, 2024. Emerg. Infect. Dis. *30*, 2370–2374.

11. Vernal, S., Martini, C.C.R., and da Fonseca, B.A.L. (2019). Oropouche Virus-Associated Aseptic Meningoencephalitis, Southeastern Brazil. Emerg. Infect. Dis. *25*, 380–382.

12. Wesselmann, K.M., Postigo-Hidalgo, I., Pezzi, L., de Oliveira-Filho, E.F., Fischer, C., de Lamballerie, X., and Drexler, J.F. (2024). Emergence of Oropouche fever in Latin America: a narrative review. Lancet Infect. Dis. *24*, e439–e452.

13. Martins-Filho, P.R., Carvalho, T.A., and Dos Santos, C.A. (2024). Oropouche fever: reports of vertical transmission and deaths in Brazil. Lancet Infect. Dis. *24*, e662–e663.

14. das Neves Martins, F.E., Chiang, J.O., Nunes, B.T.D., Ribeiro, B.F.R., Martins, L.C., Casseb, L.M.N., Henriques, D.F., de Oliveira, C.S., Maciel, E.L.N., Azevedo, R.D.S., et al. (2024). Newborns with microcephaly in Brazil and potential vertical transmission of Oropouche virus: a case series. Lancet Infect. Dis. *25*, 155.

15. de Souza, W.M., Calisher, C.H., Carrera, J.P., Hughes, H.R., Nunes, M.R.T., Russell, B., Tilson-Lunel, N.L., Venter, M., and Xia, H. (2024). ICTV Virus Taxonomy Profile: Peribunyaviridae 2024. J. Gen. Virol. *105*, 002034.

16. Travassos da Rosa, J.F., de Souza, W.M., Pinheiro, F.d.P., Figueiredo, M.L., Cardoso, J.F., Acrani, G.O., and Nunes, M.R.T. (2017). Oropouche Virus: Clinical, Epidemiological, and Molecular Aspects of a Neglected Orthobunyavirus. Am. J. Trop. Med. Hyg. *96*, 1019–1030.

17. Pinheiro, F.P., Travassos da Rosa, A.P., Gomes, M.L., LeDuc, J.W., and Hoch, A.L. (1982). Transmission of Oropouche virus from man to hamster by the midge Culicoides paraensis. Science *215*, 1251–1253.

18. Benitez, A.J., Alvarez, M., Perez, L., Gravier, R., Serrano, S., Hernandez, D.M., Perez, M.M., Gutierrez-Bugallo, G., Martinez, Y., Companioni, A., et al. (2024). Oropouche Fever, Cuba, May 2024. Emerg. Infect. Dis. *30*, 2155–2159.

19. Peinado, R.D.S., Eberle, R.J., Arni, R.K., and Coronado, M.A. (2022). A Review of Omics Studies on Arboviruses: Alphavirus, Orthobunyavirus and Phlebovirus. Viruses *14*, 2194.

20. Geddes, V.E.V., de Oliveira, A.S., Tanuri, A., Arruda, E., Ribeiro-Alves, M., and Aguiar, R.S. (2018). MicroRNA and cellular targets profiling reveal miR-217 and miR-576-3p as proviral factors during Oropouche infection. PLoS Negl. Trop. Dis. *12*, e0006508.

21. Rodrigues, A.H., Santos, R.I., Arisi, G.M., Bernardes, E.S., Silva, M.L., Rossi, M.A., Lopes, M.B.S., and Arruda, E. (2011). Oropouche virus experimental infection in the golden hamster (Mesocrisetus auratus). Virus Res. *155*, 35–41.

22. Araujo, R., Dias, L.B., Araujo, M.T., Pinheiro, F., and Oliva, O.F. (1978). [Ultrastructural changes in the hamster liver after experimental inoculation with Oropouche arbovirus (type BeAn 19991)] Alteracoes ultraestruturais no figado de hamster apos inoculacao experimental com arbovirus Oropouche (tipo BeAn 19991). Rev. Inst. Med. Trop. Sao Paulo *20*, 45–54.

23. Proenca-Modena, J.L., Sesti-Costa, R., Pinto, A.K., Richner, J.M., Lazear, H.M., Lucas, T., Hyde, J.L., and Diamond, M.S. (2015). Oropouche virus infection and pathogenesis are restricted by MAVS, IRF-3, IRF-7, and type I interferon signaling pathways in nonmyeloid cells. J. Virol. *89*, 4720–4737.

24. Proenca-Modena, J.L., Hyde, J.L., Sesti-Costa, R., Lucas, T., Pinto, A.K., Richner, J.M., Gorman, M.J., Lazear, H.M., and Diamond, M.S. (2016). Interferon-Regulatory Factor 5-Dependent Signaling Restricts Orthobunyavirus Dissemination to the Central Nervous System. J. Virol. *90*, 189–205.

25. da Silva Menegatto, M.B., Ferraz, A.C., Lima, R.L.S., Almeida, L.T., de Brito, R.C.F., Reis, A.B., Carneiro, C.M., de Lima, W.G., de Mello Silva, B., de Magalhães, J.C., and Magalhães, C.L.d.B. (2023). Oropouche virus infection induces ROS production and oxidative stress in liver and spleen of mice. J. Gen. Virol. *104*, 001857.

26. da Costa, V.G., de Rezende Féres, V.C., Saivish, M.V., de Lima Gimaque, J.B., and Moreli, M.L. (2017). Silent emergence of Mayaro and Oropouche viruses in humans in Central Brazil. Int. J. Infect. Dis. *62*, 84–85.

27. Alva-Urcia, C., Aguilar-Luis, M.A., Palomares-Reyes, C., Silva-Caso, W., Suarez-Ognio, L., Weilg, P., Manrique, C., Vasquez-Achaya, F., Del Valle, L.J., and Del Valle-Mendoza, J. (2017). Emerging and reemerging arboviruses: A new threat in Eastern Peru. PLoS One *12*, e0187897.

28. Co, A.C.G., de Mendonca, G.C., Gatti, F.D., Sousa, T.J., Tavares, E.A., Nodari, J.Z., de Moura, R.G., Lopes, P.O., Pereira, J.D.P., Alves, L.N.R., et al. (2025). Unravelling the pathogenesis of Oropouche virus. Lancet Infect. Dis. *25*, e381–e382.

29. Barbiero, A., Formica, G., Mantovani, R.P., Accordini, S., Gobbi, F., Spinicci, M., Colao, M.G., Pollini, S., Ciccone, N., Rossolini, G.M., et al. (2024). Persistent Oropouche virus viremia in two travellers returned to Italy from Cuba, July 2024. J Travel Med *32*, taae148.

30. Deiana, M., Malago, S., Mori, A., Accordini, S., Matucci, A., Passarelli Mantovani, R., Gianesini, N., Huits, R., Piubelli, C., Gobbi, F.G., et al. (2024). Full Genome Characterization of the First Oropouche Virus Isolate Imported in Europe from Cuba. Viruses *16*, 1586.

31. van Beek, J.H.D.A., de Moor, M.H.M., de Geus, E.J.C., Lubke, G.H., Vink, J.M., Willemsen, G., and Boomsma, D.I. (2013). The genetic architecture of liver enzyme levels: GGT, ALT and AST. Behav. Genet. *43*, 329–339.

32. Lefebvre, P., and Staels, B. (2021). Hepatic sexual dimorphism - implications for non-alcoholic fatty liver disease. Nat. Rev. Endocrinol. *17*, 662–670.

33. Tamber, S.S., Bansal, P., Sharma, S., Singh, R.B., and Sharma, R. (2023). Biomarkers of liver diseases. Mol. Biol. Rep. *50*, 7815–7823.

34. Marsee, A., Roos, F.J.M., Verstegen, M.M.A., Gehart, H., de Koning, E., Lemaigre, F., Forbes, S.J., Peng, W.C., Huch, M., Takebe, T., et al. (2021). Building consensus on definition and nomenclature of hepatic, pancreatic, and biliary organoids. Cell Stem Cell *28*, 816–832.

35. Huch, M., Gehart, H., van Boxtel, R., Hamer, K., Blokzijl, F., Verstegen, M.M.A., Ellis, E., van Wenum, M., Fuchs, S.A., de Ligt, J., et al. (2015). Long-term culture of genome-stable bipotent stem cells from adult human liver. Cell *160*, 299–312.

36. Arragain, B., Effantin, G., Gerlach, P., Reguera, J., Schoehn, G., Cusack, S., and Malet, H. (2020). Pre-initiation and elongation structures

CellPress
OPEN ACCESS

of full-length La Crosse virus polymerase reveal functionally important conformational changes. Nat. Commun. *11*, 3590.

37. Santos, R.I.M., Rodrigues, A.H., Silva, M.L., Mortara, R.A., Rossi, M.A., Jamur, M.C., Oliver, C., and Arruda, E. (2008). Oropouche virus entry into HeLa cells involves clathrin and requires endosomal acidification. Virus Res. *138*, 139–143.
38. de Almeida, A.L.T., da Costa, I.P.S., Garcia, M.D.d.N., da Silva, M.A.N., Lazzaro, Y.G., de Filippis, A.M.B., Nogueira, F.d.B., and Barreto-Vieira, D.F. (2025). Oropouche Virus: Isolation and Ultrastructural Characterization from a Human Case Sample from Rio de Janeiro, Brazil, Using an In Vitro System. Viruses *17*, 373.
39. Concha, J.O., Gutierrez, K., Barbosa, N., Rodrigues, R.L., de Carvalho, A.N., Tavares, L.A., Rudd, J.S., Costa, C.S., Andrade, B.Y.G., Espreafico, E.M., et al. (2024). Rab27a GTPase and its effector Myosin Va are host factors required for efficient Oropouche virus cell egress. PLoS Pathog. *20*, e1012504.
40. Schoggins, J.W., Wilson, S.J., Panis, M., Murphy, M.Y., Jones, C.T., Bieniasz, P., and Rice, C.M. (2011). A diverse range of gene products are effectors of the type I interferon antiviral response. Nature *472*, 481–485.
41. Ahrens, S., Zelenay, S., Sancho, D., Hanč, P., Kjær, S., Feest, C., Fletcher, G., Durkin, C., Postigo, A., Skehel, M., et al. (2012). F-actin is an evolutionarily conserved damage-associated molecular pattern recognized by DNGR-1, a receptor for dead cells. Immunity *36*, 635–645.
42. Suarez-Huerta, N., Mosselmans, R., Dumont, J.E., and Robaye, B. (2000). Actin depolymerization and polymerization are required during apoptosis in endothelial cells. J. Cell. Physiol. *184*, 239–245.
43. Bursch, W., Hochegger, K., Torok, L., Marian, B., Ellinger, A., and Hermann, R.S. (2000). Autophagic and apoptotic types of programmed cell death exhibit different fates of cytoskeletal filaments. J. Cell Sci. *113*, 1189–1198.
44. Tian, L., Qiang, T., Liang, C., Ren, X., Jia, M., Zhang, J., Li, J., Wan, M., Yu-Wen, X., Li, H., et al. (2021). RNA-dependent RNA polymerase (RdRp) inhibitors: The current landscape and repurposing for the COVID-19 pandemic. Eur. J. Med. Chem. *213*, 113201.
45. Zibat, A., Zhang, X., Dickmanns, A., Stegmann, K.M., Dobbelstein, A.W., Alachram, H., Soliwoda, R., Salinas, G., Groß, U., Görlich, D., et al. (2023). N4-hydroxycytidine, the active compound of Molnupiravir, promotes SARS-CoV-2 mutagenesis and escape from a neutralizing nanobody. iScience *26*, 107786.
46. Strizki, J.M., Gaspar, J.M., Howe, J.A., Hutchins, B., Mohri, H., Nair, M.S., Kinek, K.C., McKenna, P., Goh, S.L., and Murgolo, N. (2024). Molnupiravir maintains antiviral activity against SARS-CoV-2 variants and exhibits a high barrier to the development of resistance. Antimicrob. Agents Chemother. *68*, e0095323.
47. Maas, B.M., Strizki, J., Miller, R.R., Kumar, S., Brown, M., Johnson, M.G., Cheng, M., De Anda, C., Rizk, M.L., and Stone, J.A. (2024). Molnupiravir: Mechanism of action, clinical, and translational science. Clin. Transl. Sci. *17*, e13732.
48. Abramson, J., Adler, J., Dunger, J., Evans, R., Green, T., Pritzel, A., Ronneberger, O., Willmore, L., Ballard, A.J., Bambrick, J., et al. (2024). Accurate structure prediction of biomolecular interactions with AlphaFold 3. Nature *630*, 493–500.
49. Toots, M., Yoon, J.J., Cox, R.M., Hart, M., Sticher, Z.M., Makhsous, N., Plesker, R., Barrena, A.H., Reddy, P.G., Mitchell, D.G., et al. (2019). Characterization of orally efficacious influenza drug with high resistance barrier in ferrets and human airway epithelia. Sci. Transl. Med. *11*, eaax5866.
50. Liu, Y., Yang, X., Gan, J., Chen, S., Xiao, Z.X., and Cao, Y. (2022). CB-Dock2: improved protein-ligand blind docking by integrating cavity detection, docking and homologous template fitting. Nucleic Acids Res. *50*, W159–W164.
51. Kabinger, F., Stiller, C., Schmitzová, J., Dienemann, C., Kokic, G., Hillen, H.S., Höbartner, C., and Cramer, P. (2021). Mechanism of molnupiravir-induced SARS-CoV-2 mutagenesis. Nat. Struct. Mol. Biol. *28*, 740–746.
52. Wang, Y., Li, P., Solanki, K., Li, Y., Ma, Z., Peppelenbosch, M.P., Baig, M.S., and Pan, Q. (2021). Viral polymerase binding and broad-spectrum antiviral activity of molnupiravir against human seasonal coronaviruses. Virology *564*, 33–38.
53. Garcia Filho, C., Lima Neto, A.S., Maia, A.M.P.C., da Silva, L.O.R., Cavalcante, R.D.C., Monteiro, H.D.S., Marques, K.C.A., Oliveira, R.d.S., Gadelha, S.d.A.C., Nunes de Melo, D., et al. (2024). A Case of Vertical Transmission of Oropouche Virus in Brazil. N. Engl. J. Med. *391*, 2055–2057.
54. Li, P., Li, Y., Wang, Y., Liu, J., Lavrijsen, M., Li, Y., Zhang, R., Verstegen, M.M.A., Wang, Y., Li, T.C., et al. (2022). Recapitulating hepatitis E virus-host interactions and facilitating antiviral drug discovery in human liver-derived organoids. Sci. Adv. *8*, eabj5908.
55. Sanderson, T., Hisner, R., Donovan-Banfield, I., Hartman, H., Løchen, A., Peacock, T.P., and Ruis, C. (2023). A molnupiravir-associated mutational signature in global SARS-CoV-2 genomes. Nature *623*, 594–600.
56. Hoofnagle, J.H., and Seeff, L.B. (2006). Peginterferon and ribavirin for chronic hepatitis C. N. Engl. J. Med. *355*, 2444–2451.
57. Zheng, S., Wang, W., Aldahdooh, J., Malyutina, A., Shadbahr, T., Tanoli, Z., Pessia, A., and Tang, J. (2022). SynergyFinder Plus: Toward Better Interpretation and Annotation of Drug Combination Screening Datasets. Genom. Proteom. Bioinform. *20*, 587–596.
58. Chinnarasu, S., Alogaili, F., Bove, K.E., Jaeschke, A., and Hui, D.Y. (2021). Hepatic LDL receptor-related protein-1 deficiency alters mitochondrial dynamics through phosphatidylinositol 4,5-bisphosphate reduction. J. Biol. Chem. *296*, 100370.
59. Hamlin, A.N., Chinnarasu, S., Ding, Y., Xian, X., Herz, J., Jaeschke, A., and Hui, D.Y. (2018). Low-density lipoprotein receptor-related protein-1 dysfunction synergizes with dietary cholesterol to accelerate steatohepatitis progression. J. Biol. Chem. *293*, 9674–9684.
60. Schwarz, M.M., Price, D.A., Ganaie, S.S., Feng, A., Mishra, N., Hoehl, R.M., Fatma, F., Stubbs, S.H., Whelan, S.P.J., Cui, X., et al. (2022). Oropouche orthobunyavirus infection is mediated by the cellular host factor Lrp1. Proc. Natl. Acad. Sci. USA *119*, e2204706119.
61. Schwarz, M.M., Ganaie, S.S., Feng, A., Brown, G., Yangdon, T., White, J.M., Hoehl, R.M., McMillen, C.M., Rush, R.E., Connors, K.A., et al. (2023). Lrp1 is essential for lethal Rift Valley fever hepatic disease in mice. Sci. Adv. *9*, eadh2264.
62. Bermudez-Mendez, E., Angelino, P., van Keulen, L., van de Water, S., Rockx, B., Pijlman, G.P., Ciuffi, A., Kortekaas, J., and Wichgers Schreur, P.J. (2023). Transcriptomic Profiling Reveals Intense Host-Pathogen Dispute Compromising Homeostasis during Acute Rift Valley Fever Virus Infection. J. Virol. *97*, e0041523.
63. Jayk Bernal, A., Gomes da Silva, M.M., Musungaie, D.B., Kovalchuk, E., Gonzalez, A., Delos Reyes, V., Martín-Quirós, A., Caraco, Y., Williams-Diaz, A., Brown, M.L., et al. (2022). Molnupiravir for Oral Treatment of Covid-19 in Nonhospitalized Patients. N. Engl. J. Med. *386*, 509–520.
64. Li, P., Wang, Y., Lavrijsen, M., Lamers, M.M., de Vries, A.C., Rottier, R.J., Bruno, M.J., Peppelenbosch, M.P., Haagmans, B.L., and Pan, Q. (2022). SARS-CoV-2 Omicron variant is highly sensitive to molnupiravir, nirmatrelvir, and the combination. Cell Res. *32*, 322–324.
65. Nakamura, K., Fujimoto, K., Hasegawa, C., Aoki, I., Yoshitsugu, H., Ugai, H., Yatsuzuka, N., Tanaka, Y., Furihata, K., Maas, B.M., et al. (2022). A phase I, randomized, placebo-controlled study of molnupiravir in healthy Japanese to support special approval in Japan to treat COVID-19. Clin. Transl. Sci. *15*, 2697–2708.
66. Livonesi, M.C., De Sousa, R.L.M., Badra, S.J., and Figueiredo, L.T.M. (2006). In vitro and in vivo studies of ribavirin action on Brazilian Orthobunyavirus. Am. J. Trop. Med. Hyg. *75*, 1011–1016.
67. Livonesi, M.C., de Sousa, R.L.M., Badra, S.J., and Figueiredo, L.T.M. (2007). In vitro and in vivo studies of the Interferon-alpha action on distinct Orthobunyavirus. Antiviral Res. *75*, 121–128.

68. Eberhardt, J., Santos-Martins, D., Tillack, A.F., and Forli, S. (2021). AutoDock Vina 1.2.0: New Docking Methods, Expanded Force Field, and Python Bindings. J. Chem. Inf. Model. *61*, 3891–3898.

69. Trott, O., and Olson, A.J. (2010). AutoDock Vina: improving the speed and accuracy of docking with a new scoring function, efficient optimization, and multithreading. J. Comput. Chem. *31*, 455–461.

70. Naveca, F.G., Nascimento, V.A.D., Souza, V.C.d., Nunes, B.T.D., Rodrigues, D.S.G., and Vasconcelos, P.F.d.C. (2017). Multiplexed reverse transcription real-time polymerase chain reaction for simultaneous detection of Mayaro, Oropouche, and Oropouche-like viruses. Mem. Inst. Oswaldo Cruz *112*, 510–513.

71. Vasconcelos, H.B., Nunes, M.R.T., Casseb, L.M.N., Carvalho, V.L., Pinto da Silva, E.V., Silva, M., Casseb, S.M.M., and Vasconcelos, P.F.C. (2011). Molecular epidemiology of Oropouche virus, Brazil. Emerg. Infect. Dis. *17*, 800–806.

72. Gutierrez, B., Wise, E.L., Pullan, S.T., Logue, C.H., Bowden, T.A., Escalera-Zamudio, M., Trueba, G., Nunes, M.R.T., Faria, N.R., and Pybus, O.G. (2020). Evolutionary Dynamics of Oropouche Virus in South America. J. Virol. *94*, e01127-19.

73. Katoh, K., Misawa, K., Kuma, K.i., and Miyata, T. (2002). MAFFT: a novel method for rapid multiple sequence alignment based on fast Fourier transform. Nucleic Acids Res. *30*, 3059–3066.

74. Tamura, K., Stecher, G., and Kumar, S. (2021). MEGA11: Molecular Evolutionary Genetics Analysis Version 11. Mol. Biol. Evol. *38*, 3022–3027.

75. Berger, A., Drosten, C., Doerr, H.W., Stürmer, M., and Preiser, W. (2004). Severe acute respiratory syndrome (SARS)–paradigm of an emerging viral infection. J. Clin. Virol. *29*, 13–22.

76. Biacchesi, S., Skiadopoulos, M.H., Yang, L., Murphy, B.R., Collins, P.L., and Buchholz, U.J. (2005). Rapid human metapneumovirus microneutralization assay based on green fluorescent protein expression. J. Virol. Methods *128*, 192–197.

77. Li, P., Pachis, S.T., Xu, G., Schraauwen, R., Incitti, R., de Vries, A.C., Bruno, M.J., Peppelenbosch, M.P., Alam, I., Raymond, K., and Pan, Q. (2023). Mpox virus infection and drug treatment modelled in human skin organoids. Nat. Microbiol. *8*, 2067–2079.

78. Love, M.I., Huber, W., and Anders, S. (2014). Moderated estimation of fold change and dispersion for RNA-seq data with DESeq2. Genome Biol. *15*, 550.

79. Yu, G., Wang, L.G., Han, Y., and He, Q.Y. (2012). clusterProfiler: an R package for comparing biological themes among gene clusters. OMICS *16*, 284–287.

80. de Melo Iani, F.C., Pereira, F.M., de Oliveira, E.C., Rodrigues, J.T.N., Machado, M.H., Fonseca, V., Adelino, T.E.R., Guimaraes, N.R., Tome, L.M.R., Gomez, M.K.A., et al. (2025). Travel-associated international spread of Oropouche virus beyond the Amazon. J Travel Med *32*.

STAR★METHODS

KEY RESOURCES TABLE

REAGENT or RESOURCE	SOURCE	IDENTIFIER
Antibodies		
KRT19	Agilent Dako	Cat#GA61561-2
Envision Flex HRP	Agilent Dako	Cat#GV90011-2
Anti-Oropouche orthobunyavirus Gc (1:100, mouse monoclonal antibody; clone 2B5B1)	provided by Dr. Sven Reiche from Federal Research Institute for Animal Health, Germany	N/A
Human Active Caspase-3 Antibody	Bio-Techne	Cat#AF835; AB_2243952
anti-mouse immunoglobulin G (IgG) (H + L, Alexa Fluor 594)	Invitrogen	Cat#A32742; AB_2762825
Phalloidin (Alexa Fluor 488)	Invitrogen	Cat#A12379
Antifade Mounting Medium with DAPI	Vector Laboratories VECTASHIELD	Cat#H-1200-10
Bacterial and virus strains		
Oropouche virus, human, strain RIVM/OROV/TRVL9760/09/11/1967	the National Institute for Public Health and the Environment, The Netherlands	N/A
OROV strain IRCCS-SCDC_1/2024_OROV-2024	IRCCS Sacro Cuore Don Calabria (SCDC) Hospital in Italy	N/A
Biological samples		
Human adult liver organoids	Erasmus Medical Center	N/A
Human fetal liver organoids	Erasmus Medical Center	N/A
Vero cell line	Historically maintained at Laboratory of Gastroenterology and Hepatology, Erasmus Medical Center; also available at ATCC	CRL-1586
Chemicals, peptides, and recombinant proteins		
Matrigel Matrix	Corning	Cat#356231
Molnupiravir (EIDD-2801)	MedChemExpress	Cat#HY-135853
EIDD-1931 (Synonyms: β-D-N4-hydroxycytidine; NHC)	MedChemExpress	Cat#HY-125033
Interferon alpha (IFN-α) 2B human	Sigma-Aldrich	Cat#H6166
Filibuvir	MedChemExpress	Cat#HY-10118
Beclabuvir	MedChemExpress	Cat#HY-12429
Bemnifosbuvir	MedChemExpress	Cat#HY-137958A
Tegobuvir	MedChemExpress	Cat#HY-10544
Remdesivir	MedChemExpress	Cat#HY-104077
Ribavirin	Sigma-Aldrich	Cat#R9644
Pimodivir	MedChemExpress	Cat#HY-12353A
T-705 (Favipiravir)	BioVision	Cat#2778-5
Sofosbuvir	MedChemExpress	Cat#HY-15005
Dasabuvir	MedChemExpress	Cat#HY-13998
Critical commercial assays		
NucleoSpin RNA Kit	Macherey-Nagel	Cat#740955.250
PrimeScript^{TM} RT Master Mix	Takara Bio	Cat#RR036A
SYBR Green PCR Master Mix	Thermo Fisher Scientific	A46109
CytoTox 96® Non-Radioactive Cytotoxicity Assay	Promega	Cat#G1780
AlamarBlue reagent	Invitrogen	Cat#DAL1100
Deposited data		
RNA-seq raw and processed data	This paper	GEO: GSE316780

(*Continued on next page*)

Continued

REAGENT or RESOURCE	SOURCE	IDENTIFIER
Oligonucleotides		
OROV S segment for RT-qPCR: forward GACAAGTSCTCAATGCTGGTGT, reverse CGTTGTCCGGSACTGGATT	This paper	In this study
Human ALB for RT-qPCR: forward CTGCCTGCCTGTTGCCAAAGC, reverse GGCAAGGTCCGCCCTGTCATC	This paper	In this study
Human CYP3A4 for RT-qPCR: forward AGCAAAGAGCAACACAGAGCTGAA, reverse CAGAGGTGTGGGCCCTGGAAT	This paper	In this study
Human LGR5 for RT-qPCR: forward GTCAGCTGCTCCCGAATCCC, reverse TGAAACAGCTTGGGGGCACA	This paper	In this study
Human MX1 for RT-qPCR: forward GGCTGTTTACCAGACTCCGACA, reverse CACAAAGCCTGGCAGCTCTCTA	This paper	In this study
Human IFIT1 for RT-qPCR: forward GCCTTGCTGAAGTGTGGAGGAA, reverse ATCCAGGCGATAGGCAGAGATC	This paper	In this study
Human ISG15 for RT-qPCR: forward CTCTGAGCATCCTGGTGAGGAA, reverse AAGGTCAGCCAGAACAGGTCGT	This paper	In this study
Human IFI6 for RT-qPCR: forward TGATGAGCTGGTCTGCGATCCT, reverse GTAGCCCATCAGGGCACCAATA	This paper	In this study
Human IFITM3 for RT-qPCR: forward GGTCTTCGCTGGACACCAT, reverse TGTCCCTAGACTTCACGGAGTA	This paper	In this study
Human IFI44L for RT-qPCR: forward TGCACTGAGGCAGATGCTGCG, reverse TCATTGCGGCACACCAGTACAG	This paper	In this study
Software and algorithms		
GraphPad Prism8 statistics software	GraphPad Software	https://www.graphpad.com/
MEGA 11	MEGA Software	https://www.megasoftware.net/
AlphaFold3	AlphaFold Server	https://alphafoldserver.com/
CB-Dock2	Liu et al.[50]	https://cadd.labshare.cn/cb-dock2/
AutoDock Vina	Dr. Oleg Trott in the Molecular Graphics Lab[68,69]	https://vina.scripps.edu/
BIOVIA Discovery Studio Visualizer (v24.1.0.23298)	Dassault Systèmes	https://www.3ds.com/products/biovia/discovery-studio/structure-based-design/

EXPERIMENTAL MODELS AND STUDY PARTICIPANT DETAILS

Infection diagnosis and liver function tests in human participants

Liver enzyme data (ALT, AST, GGT) were collected from six imported Oropouche cases in Europe for a pilot analysis (Table S1). Next, serum samples were collected from 42 patients with confirmed OROV infection in Ceará State, Brazil, between July and August 2024 (Table S2). Patients were matched by age, sex, and the time interval between symptom onset and sample collection. All cases were confirmed by real-time RT-qPCR targeting OROV with a limit of detection of 2–20 copies, corresponding to Ct values of 34.8–38.1.[70] The positivity was confirmed in all follow-up clinical samples. All the patients showed OROV-specific antibody seroconversion confirmed by seroneutralization test. Serum levels of liver transaminases (i.e., ALT, AST, GGT, ALP, and bilirubin) were quantified using commercial colorimetric kits from (Wiener Laboratories, Argentina; Cat numbers: 867031000, 861267524, 870580000, 870600000, 867046100, 867013010). Reference ranges for liver function tests were provided by local hospitals or the kit manufacturers: ALT $\leq$41 U/L for males and $\leq$31 U/L for females, AST $\leq$38 U/L for males and $\leq$32 U/L for females, γ-GT 11–50 U/L for males

and 7–32 U/L for females, and for both sexes: ALP ≤240 U/L, total bilirubin ≤1.2 mg/dL, direct bilirubin ≤0.3 mg/dL and indirect bilirubin ≤0.8 mg/dL. All serum samples were stored at −80°C prior to analysis, following manufacturer instructions. Basic clinical and demographic data of the patients are presented in Table S2. Sex as a biological variable. Sex (female/male) was obtained from clinical records; gender was not assessed. Liver function parameters were compared between females and males (Figures 1 and S1). These sex-stratified analyses are exploratory.

Culture of human liver-derived organoids

Human fetal (n = 1 donor) and adult (n = 4 donors, 2 females and 2 males; sex was not specifically analyzed) human liver-derived organoids (ICOs) were isolated by collagenase digestion from tissue samples (≤0.5 cm^3) of donor liver biopsies.[35,54] ICOs were embedded in Matrigel (Corning, USA) and cultured in organoid expansion medium (EM), consisting of advanced DMEM/F12 (Invitrogen, USA), supplemented with 1% penicillin/streptomycin (Life Technologies, USA), 1 M HEPES (Life Technologies, USA), 200 mM ultraglutamine (Life Technologies, USA), 1% (v/v) of N2 (Gibco, USA), 2% (v/v) of B27 (Gibco, USA), 1 mM N-acetylcysteine (Sigma-Aldrich, USA), 10 mM nicotinamide (Sigma-Aldrich, USA), 5 μM A83.01 (Tocris Bioscience, UK), 10 μM forskolin (Tocris Bioscience, UK), 10 nM gastrin (Sigma-Aldrich, USA), epidermal growth factor (EGF) (50 ng/mL; PeproTech, USA), 10% (v/v) of R-spondin-1 (conditioned medium), fibroblast growth factor 10 (FGF10) (100 ng/mL; PeproTech, USA), hepatocyte growth factor (HGF) (25 ng/mL; PeproTech, USA), and 10 μM Y27632 (Sigma-Aldrich, USA). To ensure the consistent quality and the reproducibility, only early-passage organoids (up to 12 passages) were used in this study. All adult liver organoids used in this study were derived from donor livers originally obtained for transplantation. Use of liver tissues for research purposes was approved by the Erasmus MC Medical Ethical Council, and informed consent was given (MEC2006-202 for fetal liver tissues and MEC-2014-060 for adult liver tissues).

Cell culture

Vero cells were maintained in Dulbecco's modified Eagle medium (DMEM; Lonza) supplemented with 10% fetal calf serum (Hyclone, Logan, USA) and 100 U/mL penicillin-streptomycin, and cultured in a humidified incubator at 37°C and 5% CO_2. All cell cultures were confirmed to be mycoplasma negative through regularly testing by GATC Biotech (Konstanz, Germany). The laboratory implemented a policy of authentication of all cell lines using the short tandem repeat genotyping assay performed at the Department of Pathology, Erasmus Medical Center Rotterdam. However, this assay is specific for human species, while Vero cells are of monkey origin, and therefore genotyping was not performed.

METHOD DETAILS

Immunohistochemistry

Organoids were formalin-fixed (4%) for 24 h, paraffin-embedded and sectioned (4 μm) for histological examination. To assess overall morphology, sections were stained with hematoxylin and eosin (H&E) and imaged by bright-field microscopy. Additional sections were processed for immunohistochemistry (IHC) using a primary antibody against cytokeratin 19 (KRT19; clone RCK108, Agilent Dako). After deparaffinization and antigen retrieval in citrate buffer (pH 6.0) at boiling temperature for 10 min, sections were incubated with the primary antibody overnight at 4°C, followed by 1 h incubation with secondary antibody (Envision Flex HRP, Agilent Dako) at room temperature, and finally developed using DAB substrate. Slides were imaged using a standard bright-field microscope.

Hepatocyte differentiation of organoids

ICOs cultured from adult liver were expanded for 5–7 days before initiation of hepatocyte differentiation. Differentiation toward hepatocyte fate was initiated with the addition of 25 ng/mL BMP7 (25 ng/mL; PeproTech) to the EM and lasted 5 days. Subsequently, organoids were passaged with a 1:1.5 split ratio and medium was changed to human hepatocyte differentiation medium (DM): AdDMEM/F12 medium supplemented with 1% penicillin/streptomycin (Life Technologies), 1 M HEPES (Life Technologies), 200 mM ultraglutamine (Life Technologies), 1% N2 and 1% B27 (with vitamin A) and containing EGF (50 ng/mL; PeproTech), 10 nM gastrin (Sigma-Aldrich), HGF (25 ng/mL; PeproTech), FGF19 (100 ng/mL; PeproTech), 500 nM A8301 (Tocris), 10 μM DAPT (Sigma-Aldrich), BMP7 (25 ng/mL; PeproTech), and 30 μM dexamethasone (Sigma-Aldrich). DM was refreshed every 2 to 3 days for a period of 12 days[35,54]

OROV strains and viral inoculation

The historical OROV strain TRVL9760 (OROV-1967) was propagated through nine successive passages in mice by intracerebral inoculation. Subsequently, virus stocks were produced by infecting Vero cells with homogenized brain tissue from the infected mice. This strain was obtained by the National Institute for Public Health and the Environment, The Netherlands. The OROV strain IRCCS-SCDC_1/2024_OROV-2024 (OROV-2024) was isolated from an imported case in Italy.[30] Human ICOs, fetal liver-derived organoids, and hepatocyte-differentiated organoids were all infected using the same standardized protocol. Organoids were mechanically fragmented and then exposed to OROV at a dose of 10^6 PFU per ~10,000 organoids for 2 h at 37°C. To enhance the infection efficiency, the virus-organoid suspension was gently resuspended every 30 min during the incubation. Subsequently, fragmented organoids were centrifuged at 300 g for 5 min at 4°C, and the supernatant was discarded. Organoids were then washed three times

with advanced DMEM/F12 to remove residual viruses, embedded in Matrigel, and cultured in EM. Each experimental group included at least four biological replicates, defined as independent organoid cultures.

Antiviral compound treatment and IC_{50}/CC_{50} analysis

To evaluate antiviral efficacy, OROV-infected human liver organoids were treated with single-dose or serial dilutions of each compound. Organoids were first inoculated with OROV for 2 h, then were washed, re-embedded in Matrigel, and transferred into 48-well plates containing expansion medium supplemented with the respective compounds. Treated organoids were then incubated for 48, 72, or 96 h depending on the experimental design. After treatment, infected organoids and supernatants were collected for following analyses. For IC_{50} and CC_{50} analysis, relatively OROV RNA levels in organoids were quantified by RT-qPCR after 48h treatment, and cell viability was assessed by AlamarBlue assay in parallel. Dose-response curves were fitted using nonlinear regression, and IC_{50} and CC_{50} values were calculated accordingly.[52]

Phylogenic analysis

We selected 1–2 representative strains from each cluster to construct a concise phylogenetic tree that clearly distinguishes between the clusters.[71,72] Specifically, the complete genomes (L, M and S segment) of OROV-2024, OROV-1967, and six representative OROV strains (strain ID: BeAn19991, BeH 390242, BeAn 626990, Esmeraldas/206, FPM01278, LACENAM_ILMD_0461SLS), along with one Perdões virus (strain ID: BeAn789726), one Madre de Dios virus strain (strain ID: FMD 1303), and one Iquitos virus strain (strain ID: MIS-0397) were included in the phylogenetic analysis. The Jatobal virus (strain ID: BeAn 423380) was used as the outgroup. Sequences were aligned using the MAFFT 7.[73] The phylogenetic tree was reconstructed using the neighbor-joining (NJ) method with branch support evaluated using the Bootstrap method (1000 replicates) in MEGA 11.[74]

The evolutionary distances were computed using the p-distance method and are in the units of the number of base differences per site. The rate variation among sites was modeled with a gamma distribution (shape parameter = 4). The resulting phylogenetic tree was visualized and edited using MEGA 11, with bootstrap values greater than 70% shown below the branches to indicate significant support. The tree was rooted using the midpoint method, and branch lengths were scaled to represent evolutionary distances. To enhance interpretability, visual adjustments were made, including color-coding the strains from our study.

Molecular docking

We conducted molecular docking experiments following the workflow illustrated in Figure S5A. Briefly, AlphaFold3 was used to predict the RdRp protein structures of OROV-1967 and OROV-2024, with a predicted template modeling (pTM) score above 0.8, indicating high-quality structural predictions.[48] Next, the CurPocket tool within the CB-Dock2v,[50] which uses a web-based protein surface curvature cavity detection method, was employed to identify potential cavities on the predicted OROV RdRp proteins. Amino acid sites on the OROV RdRp were annotated based on previous research,[36] and the cavity affecting RNA-protein contact was selected as docking configurations. Subsequently, docking experiments between molnupiravir, NHC, NHC-5′-triphosphate and the OROV RdRp models were conducted using CB-Dock2, a protein-ligand blind docking web server powered by AutoDock Vina (version 1.1.2),[68,69] to verify the accuracy of the pocket detection process.[50] The 2D schematic diagram illustrating the intermolecular interactions between receptor and ligand molecules was generated using BIOVIA Discovery Studio Visualizer (v24.1.0.23298).

RNA extraction and RT-qPCR detection

Total RNA was extracted using the NucleoSpin RNA Kit (MACHEREY-NAGEL, Germany) and quantified by Nanodrop ND-1000 (Wilmington, USA). cDNA was synthesized using a cDNA synthesis kit (Takara Bio, Japan). Viral DNA levels were quantified by SYBR Green-based RT-qPCR (Applied Biosystems SYBR Green PCR Master Mix; Thermo Fisher Scientific) with the QuantStudio Real-Time PCR Systems (Applied Biosystems by Thermo Fisher Scientific). Relative gene expression was normalized to GAPDH using the formula $2^{-\Delta\Delta CT}$ ($\Delta\Delta CT = \Delta CTsample - \Delta CTcontrol$). Primers used for quantitative reverse transcription PCR (qRT-PCR) were synthesized by Sigma-Aldrich (USA).

Quantification of virus genome copy numbers

To determine viral genome copy numbers, cDNA from OROV infected organoids was used as template to generate the insert DNA, which was then cloned into the vector to generate the plasmid containing OROV S segment sequence. The plasmid was serially diluted 10-fold from 10^2 to 10^8 copies and quantified by RT-qPCR to establish a standard curve. The linear regression equation was determined as $Ct = -3.323 \times \log_{10}[copies] + 35.47$, with an R^2 of 0.9989. Based on the standard deviation of the residuals (Sy.x = 0.2406), the limit of detection (LOD) and limit of quantification (LOQ) were calculated as 1.65 copies and 5.31 copies per reaction, respectively. These correspond to Ct values of approximately 34.9 (LOD) and 33.1 (LOQ).

$TCID_{50}$ assay

Infectious OROV titers were determined using a 50% tissue culture infectious dose (TCID50) assay. Briefly, serial 10-fold dilutions of harvested supernatant were inoculated onto monolayers of Vero cells seeded at a density of 2,000 cells per well in 96-well plates (*n* = 8 wells per dilution). Plates were incubated at 37°C for 3–4 days and monitored daily for CPE using light microscopy. CPE was

CellPress
OPEN ACCESS

scored as present or absent in each well based on the changes of cell morphology, including cell rounding, detachment, and syncytia formation.[75] The $TCID_{50}$ value was calculated by using the Reed-Muench method.[76]

Immunofluorescence assay

Organoids were fixed in 4% paraformaldehyde solution at 4°C overnight. The slides containing organoids were then rinsed three times with phosphate-buffered saline (PBS) for 5 min each time, followed by permeabilizing with PBS containing 0.2% (v/v) Triton X-100 for 5 min. Then, the slides were twice rinsed with PBS for 5 min, followed by incubation with blocking solution (5% donkey serum, 1% bovine serum albumin, and 0.2% Triton X-100 in PBS) at room temperature for 1 h. Next, the slides were incubated in a humidity chamber with primary antibody diluted in blocking solution at 4°C overnight. Primary antibodies used in this study are as follows: Anti-Oropouche orthobunyavirus Gc (1:100, mouse monoclonal antibody; clone 2B5B1; provided by Dr. Sven Reiche from Federal Research Institute for Animal Health, Germany) and Human Active Caspase-3 Antibody (R&D Systems, Bio-Techne, USA). Slides were washed three times for 5 min each in PBS before 1-h incubation with 1:1000 dilutions of the anti-mouse immunoglobulin G (IgG) (H + L, Alexa Fluor 594, Invitrogen, USA) secondary antibodies and 20-min incubation with 1:200 Phalloidin (Alexa Fluor 488, Invitrogen, USA). Nuclei were stained with DAPI (VECTASHIELD Vector Laboratories, USA). Images were obtained using Leica SP5 cell imaging system.

LDH release assay

Organoid culture supernatants were collected at the indicated time points, and LDH release was quantified using the CytoTox 96 Non-Radioactive Cytotoxicity Assay (Promega, USA) following the manufacturer's instructions. Briefly, 50 μL of each sample was transferred to a 96-well plate and incubated with 50 μL of reconstituted substrate mix at room temperature for 30 min in the dark. The reaction was stopped by adding 50 μL of stop solution, and absorbance was measured at 490 nm using a microplate reader (Thermo Fisher Scientific, USA).

AlamarBlue assay

Culture supernatant was discarded, and the organoids were incubated with a 1:20 dilution of AlamarBlue reagent (Invitrogen, USA) in culture medium for 2 h at 37°C. Subsequently, 100 μL of the medium was collected to assess cell metabolic activity, with each sample being measured in triplicate. Absorbance measurements were obtained using a CytoFluor Series 4000 plate reader (Thermo Fisher Scientific, USA) at an excitation wavelength of 530/25 nm and an emission wavelength of 590/35 nm.

Genome-wide RNA sequencing and data analysis

Organoids cultured from adult human liver tissue were infected with both OROV isolates as described above. Infected organoids were harvested at 1- 48-, and 96-h post-infection. A treatment group was established using organoids infected with the OROV-2024 isolate and treated with 1 μM NHC treatment from 1 h to 96 h post-inoculation. As negative controls, uninfected organoids were cultured under same conditions for 96 h. Total RNA was extracted from all samples using the MachereyNagel NucleoSpin RNA II Kit (Bioke, Netherlands) and quantified with the Bioanalyzer RNA 6000 Picochip. Briefly, mRNA was purified from total RNA using poly-T oligo-attached magnetic beads for cDNA synthesis and library construction by Novogene. The library was checked with Qubit and real-time PCR for quantification and bioanalyzer for size distribution detection. RNA sequencing was conducted using a paired-end 150 bp (PE 150) sequencing strategy via Illumina platforms.[77] RNA sequencing datasets have been deposited to NCBI GEO: GSE316780 at https://www.ncbi.nlm.nih.gov/geo/query/acc.cgi?acc=GSE316780.

Novogene provided a standard bioinformatic analysis for the host (human) mRNA sequencing data of with a well-annotated reference genome. Gene expression levels were quantified as fragments per kilobase of transcript per million mapped reads (FPKM). The coexpression Venn diagram showed the number of genes uniquely or commonly expressed across groups. Differential gene expression analysis was performed for comparison between two groups,[78] and the results were visualized using volcano plots. Functional annotation was conducted using the clusterProfiler package for gene ontology (GO) enrichment analysis,[79] and gene set enrichment analysis (GSEA) was used to evaluate pathway-level transcriptional changes. For heatmap visualization, gene expression values were row-wise *Z* score normalized across different groups, and we used the same uninfected group as a background reference to ensure comparability across conditions.

Passaging OROV under NHC pressure and nanopore sequencing

Vero cells were seeded in 6-well plates on the day prior to inoculation with OROV. Independent triplicate cultures were infected with OROV and in the presence of NHC and triplicates with culture medium only served as the control. OROV was inoculated into each well at 10^5 $TCID_{50}$ for the initial culture at the final volume of 2 mL per well, and NHC was added starting at 0.01 μM. Every two days, infected cultures were harvested by freeze-thawing the cells together with the supernatant. 100 μL of the collected virus-containing medium was then used to infect fresh Vero cells, and the NHC concentration was increased at each passage. Passaging continued until the drug concentration reached 9 μM, after which samples from the final passage was collected for Nanopore sequencing.

Briefly, whole genome sequencing of the OROV stock and cultured isolates was performed using amplicon-based sequencing as previously described on the Nanopore platform.[80] Consensus sequence analysis was performed using a custom build Nanopore

analysis pipeline (https://github.com/EMC-Viroscience/nanopore-amplicon-analysis-manual). Minor variant analysis was performed using Genenious Prime version 2026.0.1 using a minimum of 30x coverage and a minimum of 20% variant frequency.

QUANTIFICATION AND STATISTICAL ANALYSIS

Statistical analysis was performed using GraphPad Prism8 statistics software (GraphPad, USA). Patient data are presented as values measured from individual patients. Experimental data are presented as mean ± standard error of the mean (SEM), based on biological replicates from at least 2 independent experiments, unless specified. All statistical details of experiments can be found in the figure legends. Comparison between two groups was analyzed by Mann-Whitney U test. For longitudinal data, a mixed-effects model (restricted maximum likelihood, REML) was used to assess the effects of time, treatment, and their interaction. When applicable, Dunnett's multiple comparisons test was used for post hoc analysis to compare treatment groups against the control. Asterisks indicated the degree of significant differences compared with the controls (*$p < 0.05$; **$p < 0.01$; ***$p < 0.001$; ****$p < 0.0001$).

Cell Reports Medicine, Volume *7*

Supplemental information

Human liver-derived organoids recapitulate Oropouche virus infection and manifestation, enabling antiviral drug discovery

Jiajing Li, Xin Wang, Yibo Ding, Fang Qin, Shirlene T.S. de Lima, Lito Papamichail, Rick Schraauwen, Julia Forato, Ingra M. Claro, Xinyi Hua, Leda M. Simões Mello, Dewy Mae Offermans, Monique M.A. Verstegen, Marjan Boter, Maikel P. Peppelenbosch, Anna Barbiero, Elisabetta Pagani, Harry L.A. Janssen, José A. Telmos Silva, Magnun N.N. dos Santos, Eder C. Pincinato, José Luiz Proenca-Modena, Pengfei Li, Adam A. Anas, Luc J. W. van der Laan, Concetta Castilletti, Bas B. Oude Munnink, William M. de Souza, Wenshi Wang, and Qiuwei Pan

Supplementary Information

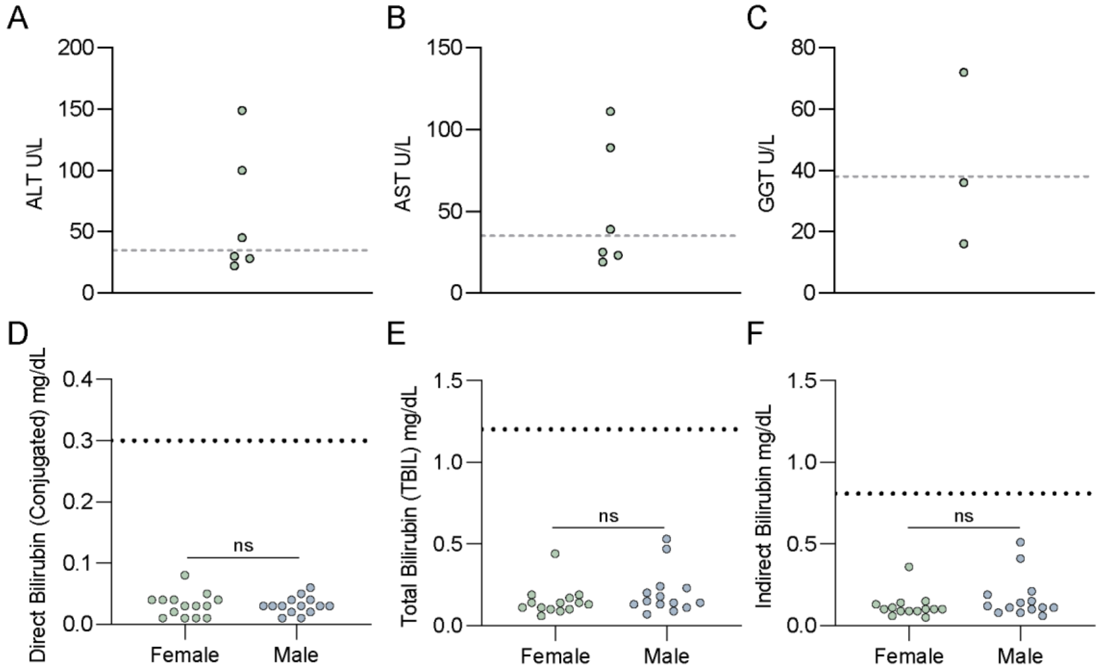

Figure S1. Liver enzyme and bilirubin levels in patients with OROV infection from Europe and Brazil. Related to Figure 1.

(A) ALT levels in OROV-infected cases imported to Europe with a threshold of 35 U/L. (B) AST levels with a threshold of 35 U/L. (C) GGT levels with a threshold of 38 U/L. (D) Direct bilirubin levels in OROV-infected cases recruited from a cohort in Brazil with a threshold of 0.3 mg/dL. (E) Total bilirubin levels with a threshold of 1.2 mg/dL. (F) Indirect bilirubin levels with a threshold of 0.8 mg/dL. The threshold references were showed as dotted line and provided by local hospitals or determined by the kit manufacturers. ALT: alanine aminotransferase; AST: aspartate aminotransferase; GGT: gamma-glutamyl transferase. Data represent measured values from individual patients. P values determined by Mann-Whitney U test. ns, not significant.

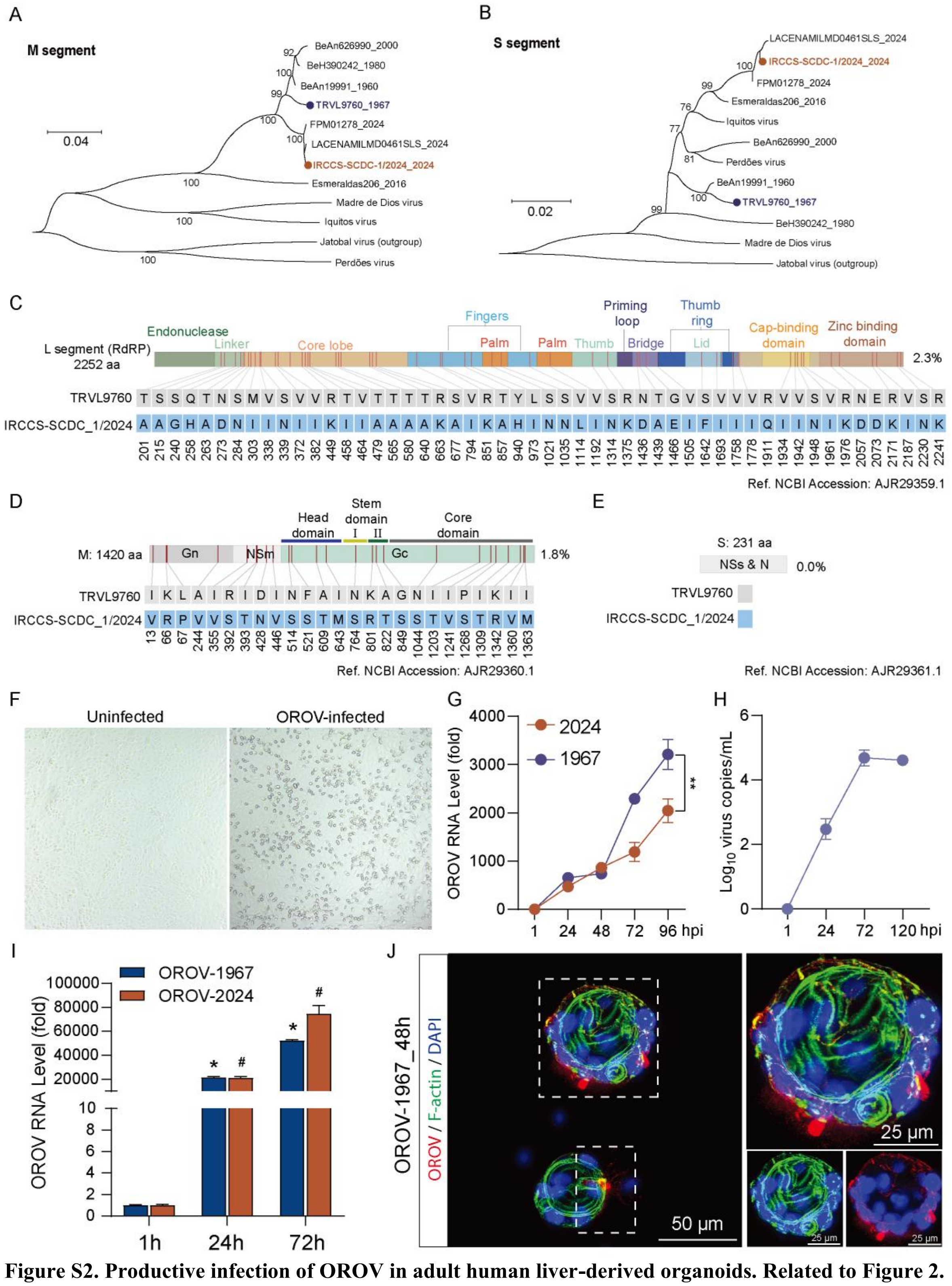

Figure S2. Productive infection of OROV in adult human liver-derived organoids. Related to Figure 2. (A, B) Phylogenetic analysis of OROV M (A) and S (B) segments. The neighbor-joining (NJ) trees were constructed using the p-distance model and evaluated with 1,000 bootstrap replicates. Only bootstrap values greater than 70% are shown. Trees are midpoint-rooted. (C–E) Amino acid substitutions between TRVL9760 and IRCCS-SCDC_1/2024 strains in the L (D), M (D), and S (E) segments. Only differing residues are shown. (F) Representative images of Vero cells showing OROV-induced cytopathic effects compared with uninfected controls. (G) Relative OROV RNA levels in organoids post-inoculation with the OROV-1967 and OROV-2024

isolates (0 in x axis represent 1-hour post-infection; n = 4 biological replicates). (H) Quantification of extracellular OROV RNA levels in cultured medium (n = 4 biological replicates). (I) Intracellular OROV RNA levels in human hepatoma-derived Huh7 cells infected with OROV-1967 or OROV-2024 at 1-, 24-, and 72-hours post-infection (n = 4 biological replicates). (J) Immunofluorescence images of organoids 48 hours post-inoculation with OROV-1967, stained with the antibody against OROV Gc glycoprotein (red), phalloidin for actin (green) and DAPI for nuclei (blue). Scale bar, 50 µm and 25 µm. Data represent mean ± SEM. Statistical analysis for longitudinal data were performed using mixed-effects model with restricted maximum likelihood (REML) (G). Statistical comparisons between different time points were performed using two-tailed Mann-Whitney U tests (I). $*p < 0.05$, $\#p < 0.05$, $**p < 0.01$.

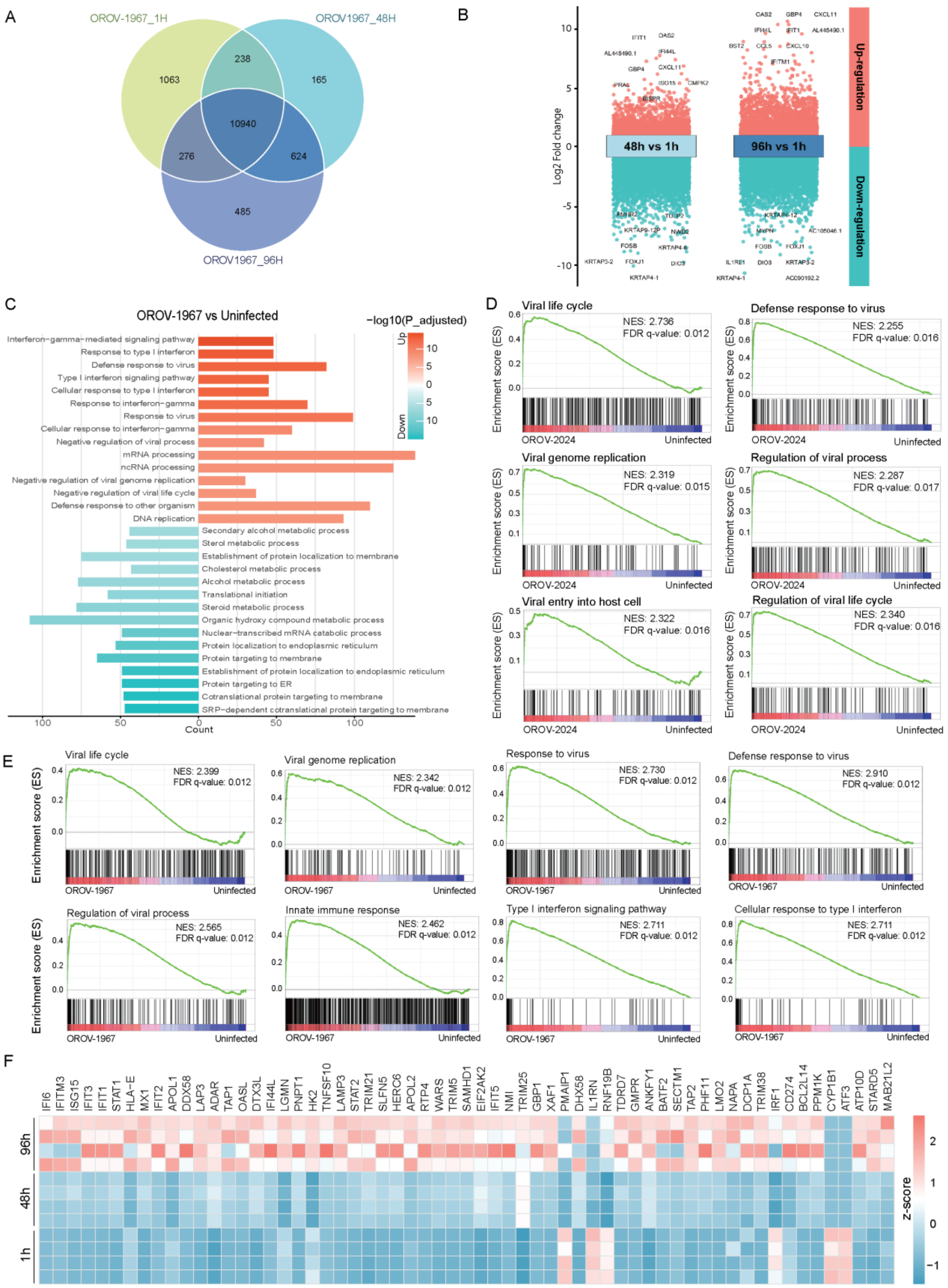

Figure S3. Transcriptomic analyses of host responses to OROV infection. Related to Figure 3.

(A) Venn diagram of overlapped differentially expressed genes in the OROV-1967 strain infected organoids at different time points. (B) Volcano plot analysis of differentially expressed genes at 48 and 96 hours compared to 1 hour after OROV-1967 inoculation. (C) Top 15 up-regulated and top 15 down-regulated pathways identified by gene ontology (GO) analysis in OROV-1967 infected organoids at 96 hours post-inoculation compared to the

uninfected controls cultured at the same condition for 96 hours. (D) Gene set enrichment analysis (GSEA) in OROV-2024 infected organoids at 96 hours post-inoculation compared to uninfected controls. (E) GSEA in OROV-1967 infected organoids at 96 hours post-inoculation compared to uninfected controls. (F) Heatmap showing the expression of selected interferon-stimulated genes (ISGs) in OROV-1967 infected organoids at 1, 48, and 96 hours post-inoculation. Data are representative of four biological replicates from one RNA-seq experiment using two independent OROV isolates and multiple time points. The color scale represents row-wise Z-score of gene expression across samples.

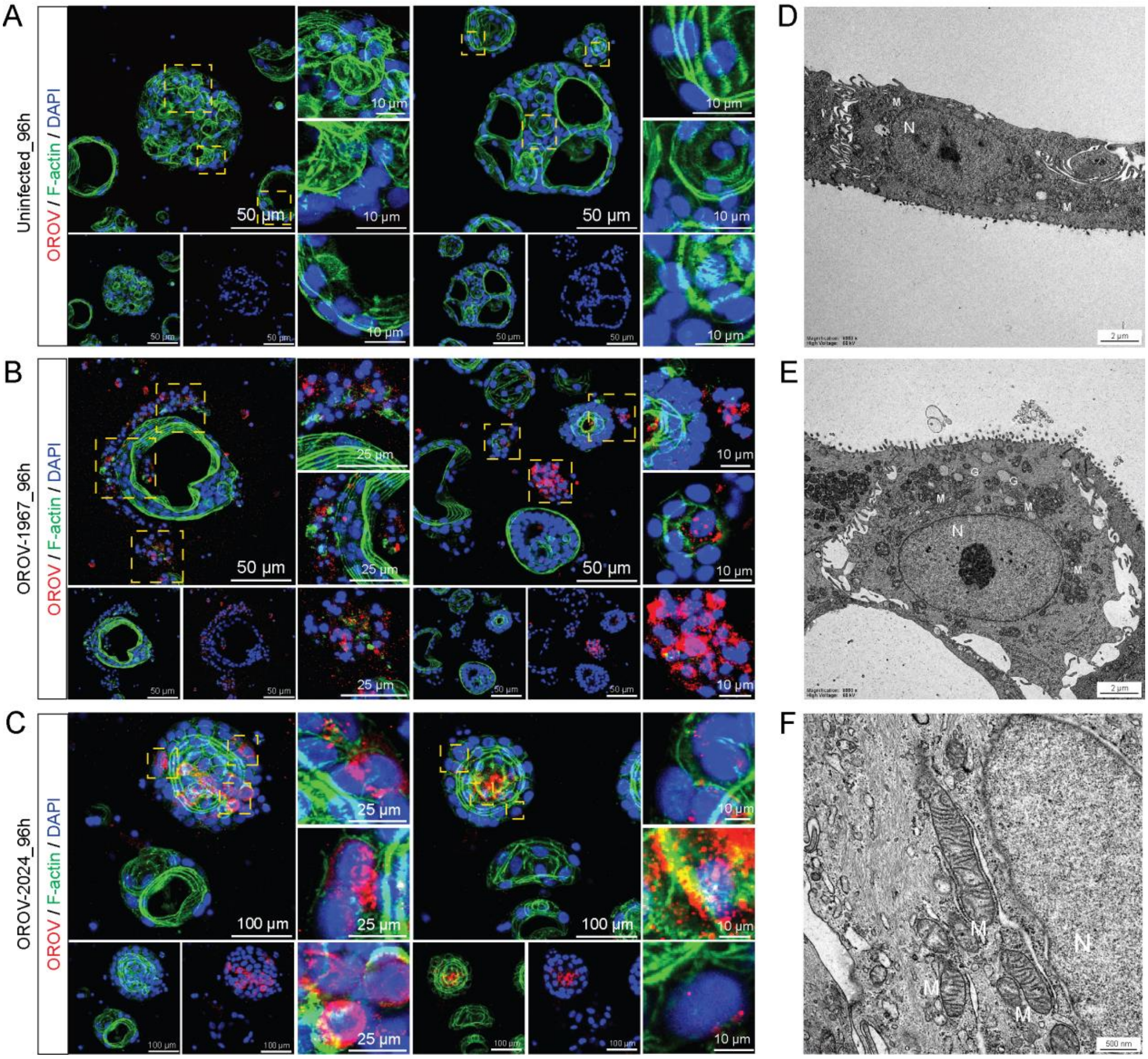

Figure S4. Cellular pathogenic alterations in OROV-infected organoids. Related to Figure 4.

(A-C) Immunofluorescence images of uninfected organoids (A), organoids 96 hours post-inoculation with OROV-1967 (B), and organoids 96 hours post-inoculation with OROV-2024 (C), stained with the antibody against OROV Gc glycoprotein (red), phalloidin (green) and DAPI nuclei staining (blue). Scale bar, 100, 50, 25 and 10 μm. (D-F) Representative TEM images visualized relatively intact mitochondria in organoids 96 hours post-inoculation with OROV-1967. Scale bars, 2 μm in D, E; 500 nm in F. N, nuclear. M, mitochondria. G, Golgi complex.

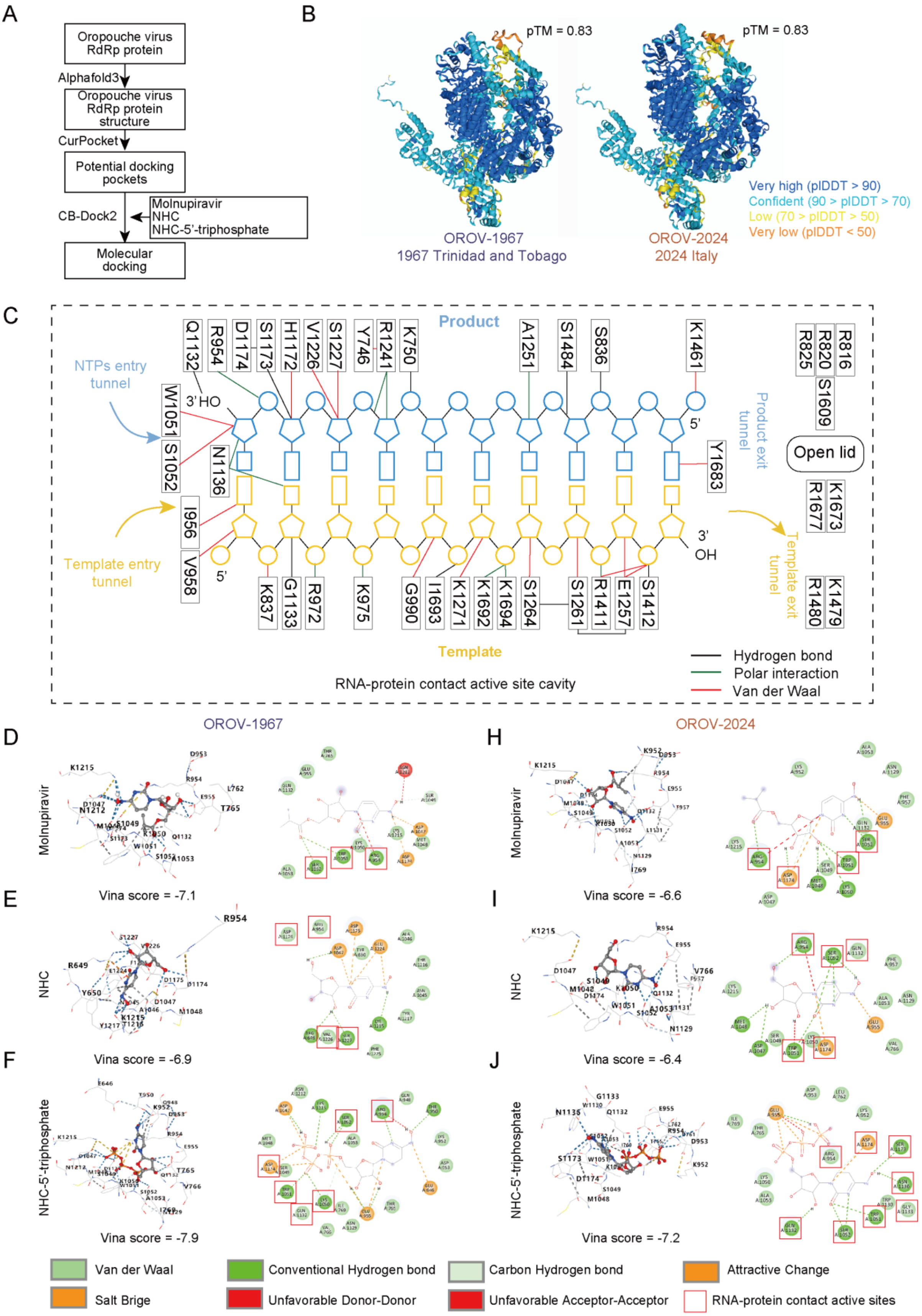

A
Oropouche virus RdRp protein
Alphafold3
Oropouche virus RdRp protein structure
CurPocket
Potential docking pockets
CB-Dock2
Molnupiravir
NHC
NHC-5'-triphosphate
Molecular docking
B
pTM = 0.83
pTM = 0.83
OROV-1967
1967 Trinidad and Tobago
OROV-2024
2024 Italy
Very high (plDDT > 90)
Confident (90 > plDDT > 70)
Low (70 > plDDT > 50)
Very low (plDDT < 50)
C
Product
NTPs entry tunnel
Q1132
R954
D1174
S1173
H1172
V1226
S1227
Y746
R1241
K750
A1251
S1484
S836
K1461
R825
R820
R816
S1609
3' HO
W1051
S1052
N1136
5'
Product exit tunnel
Open lid
Y1683
R1677
K1673
I956
V958
Template entry tunnel
3'
OH
Template exit tunnel
5'
K837
G1133
R972
K975
G990
I1693
K1271
K1692
K1694
S1264
S1261
R1411
E1257
S1412
R1480
K1479
Template
RNA-protein contact active site cavity
Hydrogen bond
Polar interaction
Van der Waal
OROV-1967
OROV-2024
D
H
Molnupiravir
Vina score = -7.1
Vina score = -6.6
E
I
NHC
Vina score = -6.9
Vina score = -6.4
F
J
NHC-5'-triphosphate
Vina score = -7.9
Vina score = -7.2
Van der Waal
Conventional Hydrogen bond
Carbon Hydrogen bond
Attractive Change
Salt Brige
Unfavorable Donor-Donor
Unfavorable Acceptor-Acceptor
RNA-protein contact active sites

Figure S5. Site specific binding mode of molnupiravir, NHC, and NHC-5'-triphosphate to OROV RdRp. Related to Figure 5.

(A) Workflow of this study. (B) The RdRp protein structures predicted by Aphafold3. (C) Schematic representation of RNA–protein contacts in the active site cavity. Drugs bind at the NTPs entry tunnel, interacting with key residues such as R954, W1051, S1052, Q1132, N1136, H1172, D1174. (D-F) The docking poses and binding residues of molnupiravir (D), NHC (E), and NHC-5'-triphosphate (F) with OROV RdRp protein of OROV-1967. (H-J) The docking poses and binding residues of molnupiravir (H), NHC (I), and NHC-5'-triphosphate (J) with OROV RdRp protein of OROV-2024.

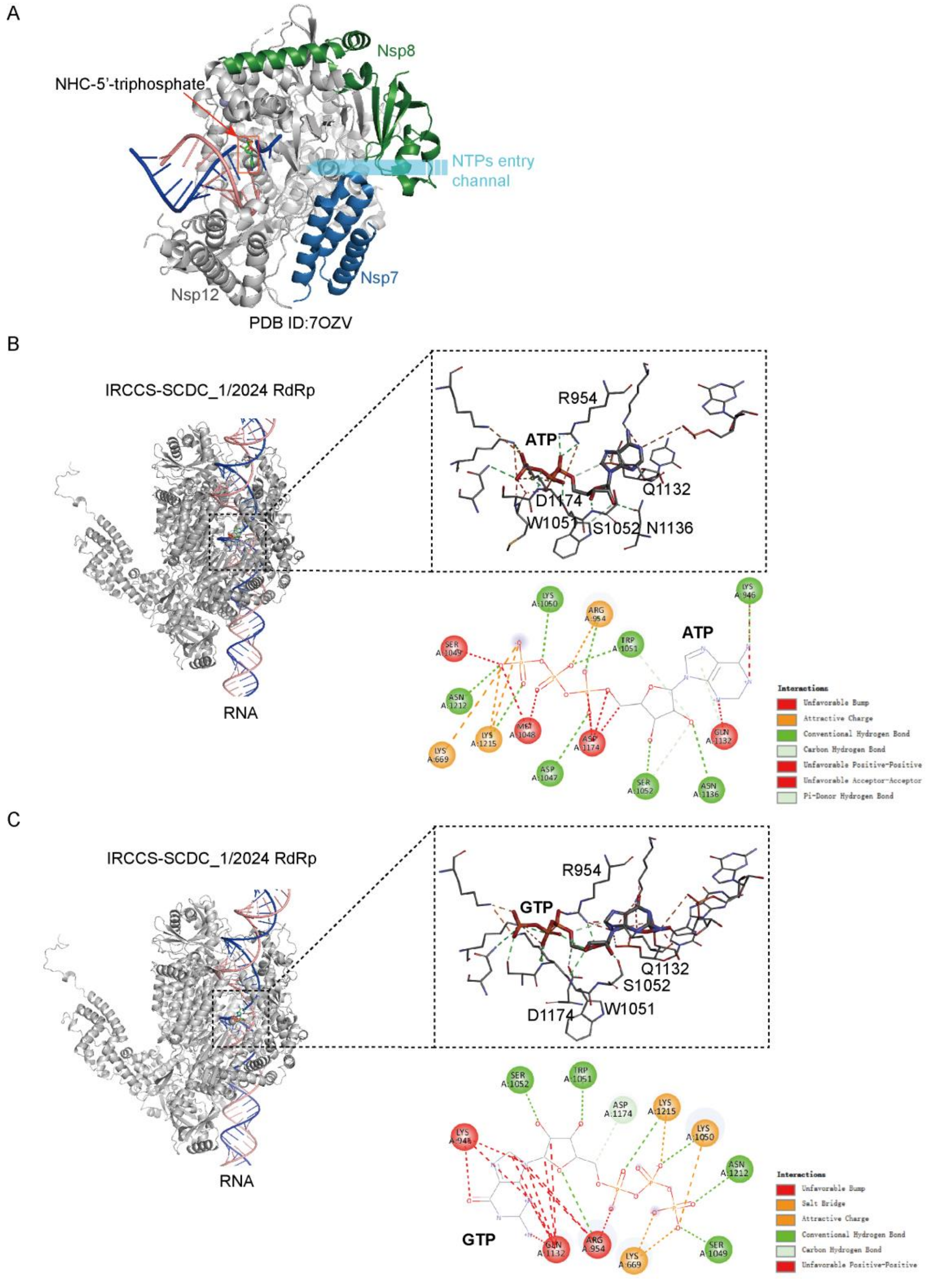

Figure S6. Schematic of molnupiravir's antiviral mechanism. Related to Figure 5.

(A) Crystal structure of SARS-CoV-2 RdRp bound to molnupiravir's active form, NHC-5′-triphosphate, in the template strand paired with G (PDB 7OZV).

(B, C) Docking poses of ATP (B) and GTP (C) in Oropouche virus (OROV) RdRp. AlphaFold3-guided docking of (B) ATP and (C) GTP into the IRCCS-SCDC_1/2024 RdRp key active sites (e.g., R954, W1051, S1052, Q1132, N1136, D1174).

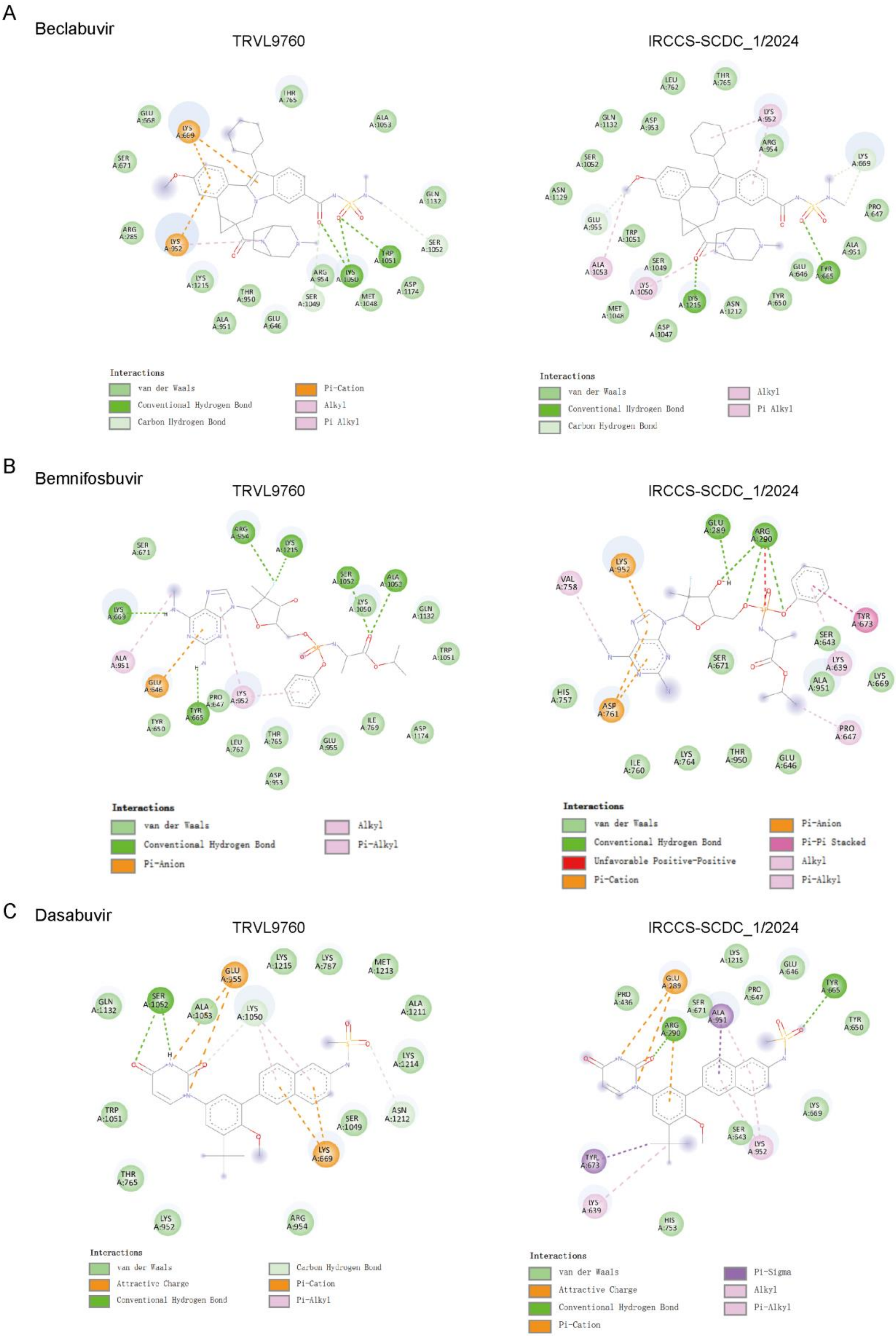
A
Beclabuvir
TRVL9760
IRCCS-SCDC_1/2024
Interactions
van der Waals
Conventional Hydrogen Bond
Carbon Hydrogen Bond
Pi-Cation
Alkyl
Pi Alkyl
B
Bemnifosbuvir
TRVL9760
IRCCS-SCDC_1/2024
Interactions
van der Waals
Conventional Hydrogen Bond
Pi-Anion
Alkyl
Pi-Alkyl
Unfavorable Positive-Positive
Pi-Cation
Pi-Pi Stacked
C
Dasabuvir
TRVL9760
IRCCS-SCDC_1/2024
Interactions
van der Waals
Attractive Charge
Conventional Hydrogen Bond
Carbon Hydrogen Bond
Pi-Cation
Pi-Alkyl
Pi-Sigma
Alkyl

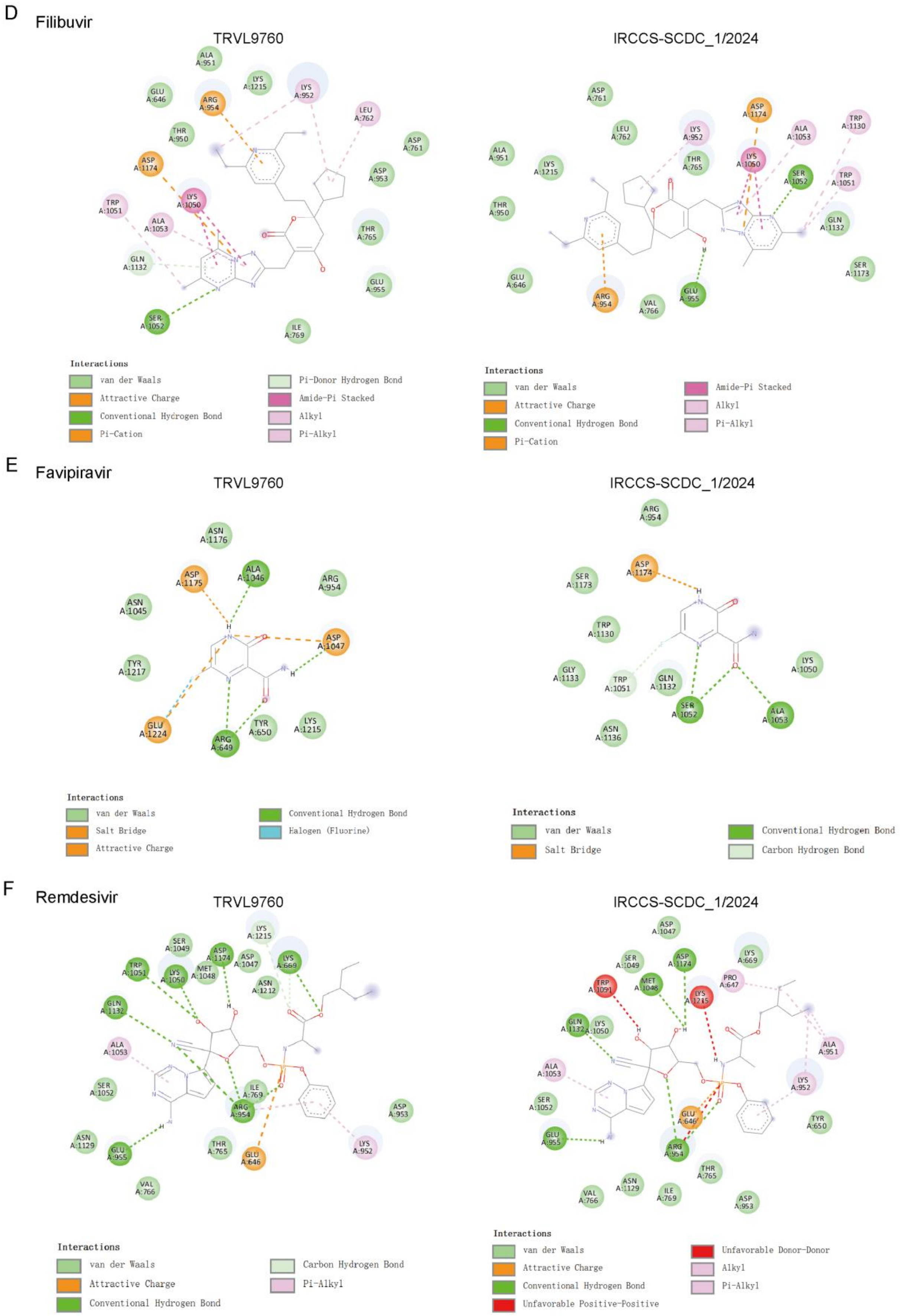
D
Filibuvir
TRVL9760
IRCCS-SCDC_1/2024
Interactions
van der Waals
Attractive Charge
Conventional Hydrogen Bond
Pi-Cation
Pi-Donor Hydrogen Bond
Amide-Pi Stacked
Alkyl
Pi-Alkyl
E
Favipiravir
TRVL9760
IRCCS-SCDC_1/2024
Interactions
van der Waals
Salt Bridge
Attractive Charge
Conventional Hydrogen Bond
Halogen (Fluorine)
Carbon Hydrogen Bond
F
Remdesivir
TRVL9760
IRCCS-SCDC_1/2024
Interactions
van der Waals
Attractive Charge
Conventional Hydrogen Bond
Carbon Hydrogen Bond
Pi-Alkyl
Unfavorable Positive-Positive
Unfavorable Donor-Donor
Alkyl

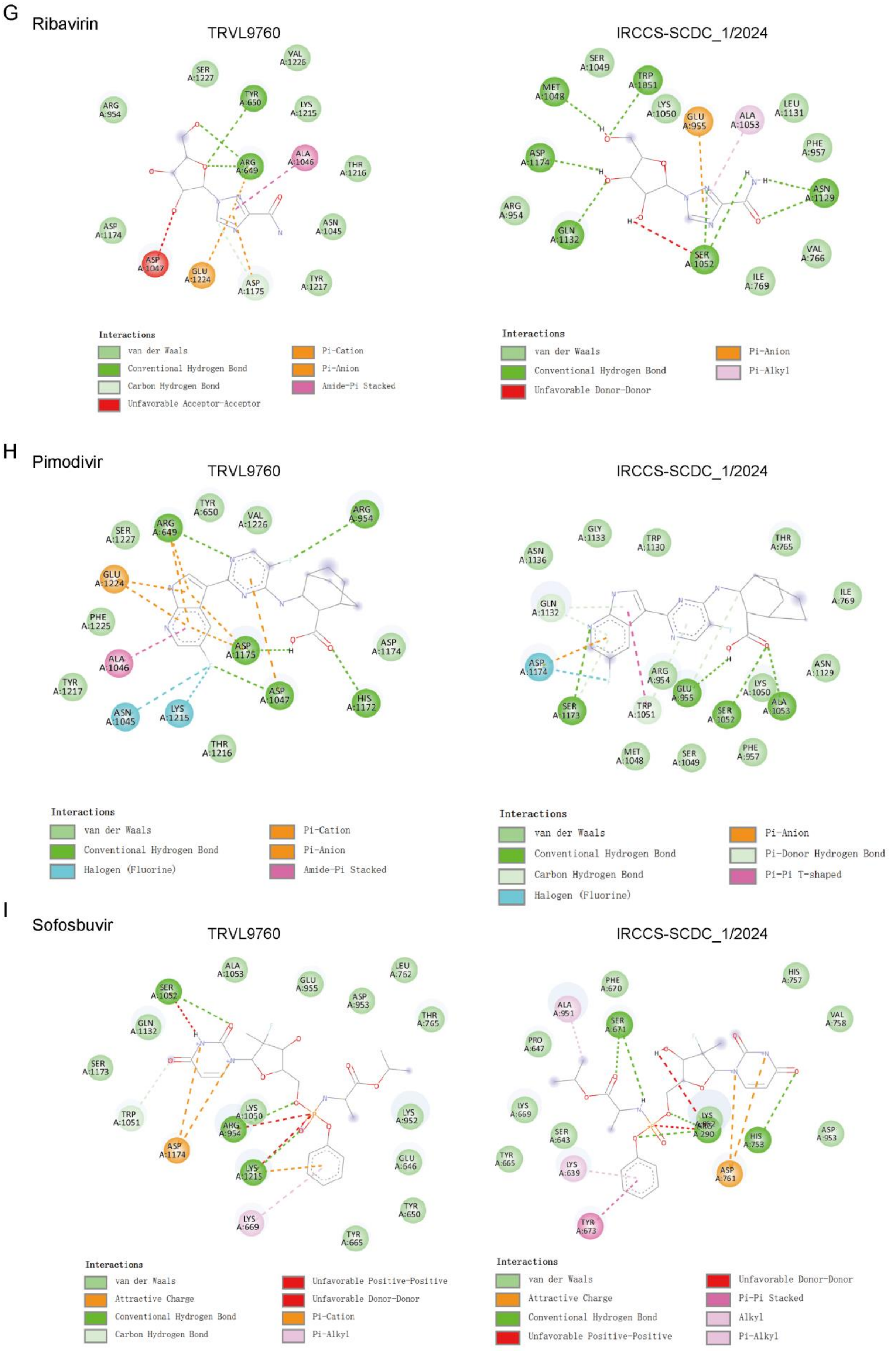
G
Ribavirin
TRVL9760
IRCCS-SCDC_1/2024
Interactions
van der Waals
Conventional Hydrogen Bond
Carbon Hydrogen Bond
Unfavorable Acceptor-Acceptor
Pi-Cation
Pi-Anion
Amide-Pi Stacked
Interactions
van der Waals
Conventional Hydrogen Bond
Unfavorable Donor-Donor
Pi-Anion
Pi-Alkyl
H
Pimodivir
TRVL9760
IRCCS-SCDC_1/2024
Interactions
van der Waals
Conventional Hydrogen Bond
Halogen (Fluorine)
Pi-Cation
Pi-Anion
Amide-Pi Stacked
Interactions
van der Waals
Conventional Hydrogen Bond
Carbon Hydrogen Bond
Halogen (Fluorine)
Pi-Anion
Pi-Donor Hydrogen Bond
Pi-Pi T-shaped
I
Sofosbuvir
TRVL9760
IRCCS-SCDC_1/2024
Interactions
van der Waals
Attractive Charge
Conventional Hydrogen Bond
Carbon Hydrogen Bond
Unfavorable Positive-Positive
Unfavorable Donor-Donor
Pi-Cation
Pi-Alkyl
Interactions
van der Waals
Attractive Charge
Conventional Hydrogen Bond
Unfavorable Positive-Positive
Unfavorable Donor-Donor
Pi-Pi Stacked
Alkyl
Pi-Alkyl

J

Tegobuvir

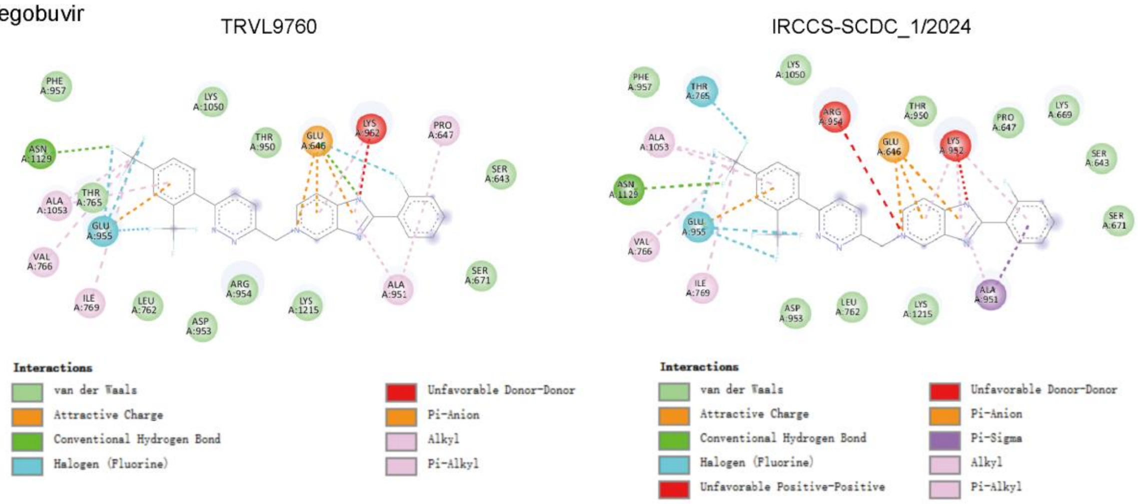

K

Prediction of antiviral drug binding characteristics and affinities to the RdRp of IRCCS-SCDC_1/2024 strain

Drugs	Category	Interaction with key residues*	CB-Dock2 Score	Calculated Affinity (kcal/mol)
NHC-5'-triphosphate	Nucleotide Analogue	R954, W1051, S1052, Q1132, N1136, S1173, D1174	-7.00	-7.06
Beclabuvir	Non-nucleoside RNA polymerase inhibitor	/	-9.00	-7.05
Bemnifosbuvir	Nucleoside RNA polymerase inhibitor	/	-8.00	-7.28
Dasabuvir	Non-nucleoside RNA polymerase inhibitor	/	-9.00	-8.21
Filibuvir	Non-nucleoside RNA polymerase inhibitor	R954, W1051, S1052, D1174	-8.90	-8.36
Favipiravir	Nucleotide Analogue	W1051, S1052, D1174	-5.90	-6.02
Remdesivir	Nucleotide Analogue	R954, W1051, Q1132, D1174	-8.10	-6.95
Ribavirin	Nucleotide Analogue	W1051, S1052, Q1132, D1174	-6.60	-6.36
Pimodivir	Non-nucleoside RNA polymerase inhibitor	W1051, S1052, Q1132, S1173, D1174	-8.50	-8.50
Sofosbuvir	Nucleoside RNA polymerase inhibitor	/	-7.80	-7.32
Tegobuvir	Non-nucleoside RNA polymerase inhibitor	R954	-9.30	-10.03

*Interactions listed represent specific contacts (e.g., hydrogen bonds, π–π stacking, salt bridges, or metal coordination) predicted by molecular docking. Non-specific van der Waals interactions were not listed.

Figure S7. Docking of antiviral drug candidates to OROV RdRp. Related to Figure 5.

(A-J) Molecular docking of (A) Beclabuvir, (B) Bemnifosbuvir, (C) Dasabuvir, (D) Filibuvir, (E) Favipiravir, (F) Remdesivir, (G) Ribavirin, (H) Pimodivir, (I) Sofosbuvir, and (J) Tegobuvir into the active site of Oropouche virus (OROV) RdRp. Explicit interactions, including hydrogen bonds, π–π stacking, salt bridges and metal coordination are indicated by dashed lines; residues depicted without dashed lines are within van der Waals contact distance (≤4 Å) of the ligand. (K) Summary of each compound's drug class, interaction with key residues, CB-Dock2 score (unitless), and calculated binding affinity (kcal/mol, from SwissDock using the AutoDock Vina engine) against IRCCS-SCDC_1/2024 OROV RdRp.

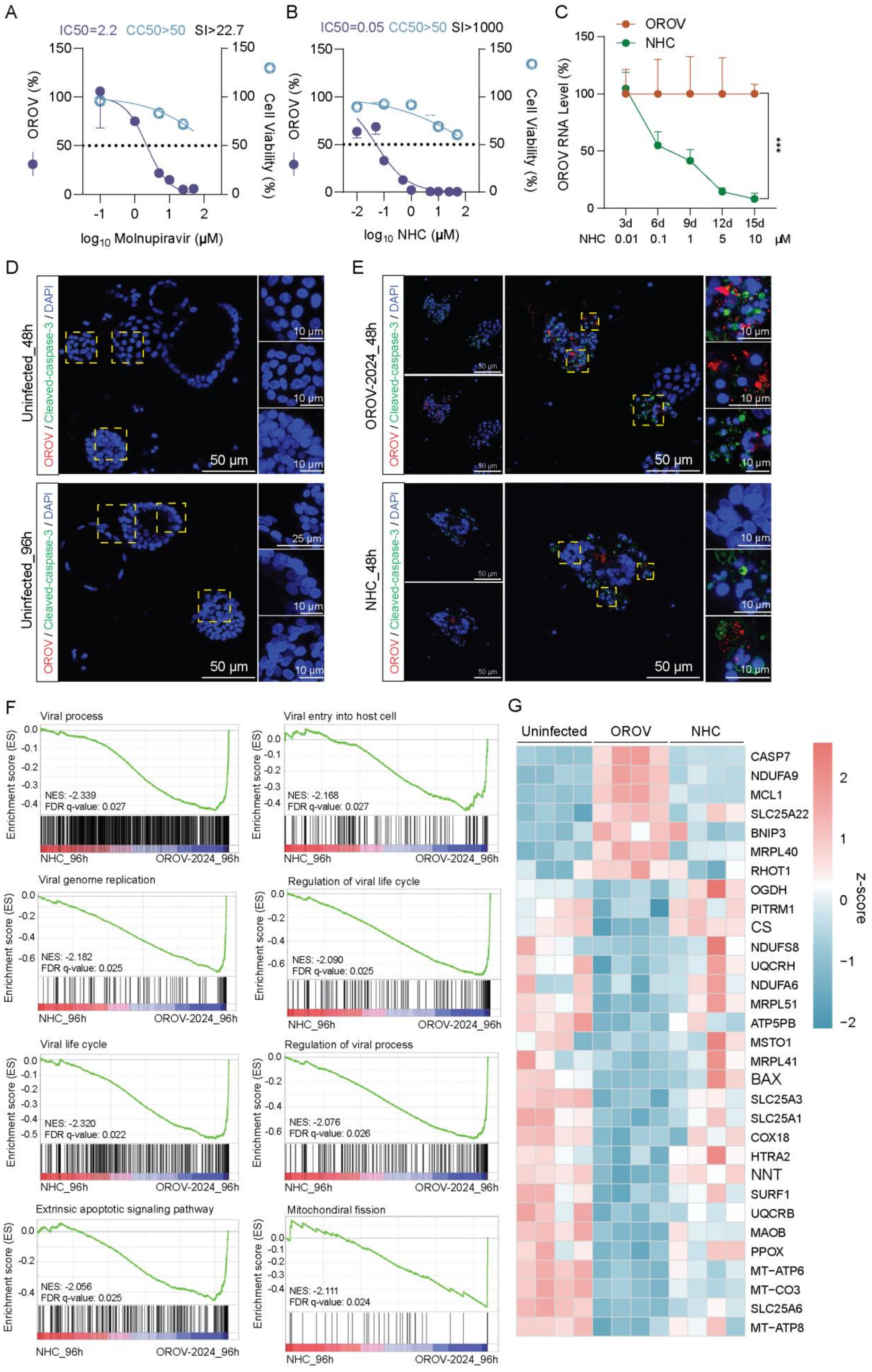
A
IC50=2.2 CC50>50 SI>22.7
OROV (%)
Cell Viability (%)
log10 Molnupiravir (μM)
B
IC50=0.05 CC50>50 SI>1000
OROV (%)
Cell Viability (%)
log10 NHC (μM)
C
OROV
NHC
OROV RNA Level (%)
***
3d 6d 9d 12d 15d
NHC 0.01 0.1 1 5 10 μM
D
Uninfected_48h
Uninfected_96h
OROV / Cleaved-caspase-3 / DAPI
50 μm
10 μm
25 μm
E
OROV-2024_48h
NHC_48h
OROV / Cleaved-caspase-3 / DAPI
50 μm
10 μm
F
Viral process
NES: -2.339
FDR q-value: 0.027
Viral entry into host cell
NES: -2.168
FDR q-value: 0.027
Viral genome replication
NES: -2.182
FDR q-value: 0.025
Regulation of viral life cycle
NES: -2.090
FDR q-value: 0.025
Viral life cycle
NES: -2.320
FDR q-value: 0.022
Regulation of viral process
NES: -2.076
FDR q-value: 0.026
Extrinsic apoptotic signaling pathway
NES: -2.056
FDR q-value: 0.025
Mitochondrial fission
NES: -2.111
FDR q-value: 0.024
Enrichment score (ES)
NHC_96h
OROV-2024_96h
G
Uninfected
OROV
NHC
CASP7
NDUFA9
MCL1
SLC25A22
BNIP3
MRPL40
RHOT1
OGDH
PITRM1
CS
NDUFS8
UQCRH
NDUFA6
MRPL51
ATP5PB
MSTO1
MRPL41
BAX
SLC25A3
SLC25A1
COX18
HTRA2
NNT
SURF1
UQCRB
MAOB
PPOX
MT−ATP6
MT−CO3
SLC25A6
MT−ATP8
z-score

Figure S8. Antiviral effect of molnupiravir in organoids inoculated with OROV. Related to Figure 5 & 6. (A, B) The 50% inhibitory concentration (IC50) and 50% cytotoxic concentration (CC50) of molnupiravir (A) or NHC (B) determined in the OROV-1967 strain infected organoids (n = 4 biological replicates). (C) Viral RNA levels in the supernatant of OROV-infected organoids during a 15-day NHC treatment using a stepwise dose-escalation approach, starting at 0.01 μM and increasing to 10 μM (n = 4 biological replicates). (D) Representative images of uninfected organoids at 48 (top) and 96 (bottom) hours by immunostaining with the antibody against OROV Gc glycoprotein (red), cleaved-caspase-3 (green) and nuclei (DAPI, blue). Scale bar, 50, 25 and 10 μm. (E) Representative images of OROV-2024 infected organoids without (top) or with (bottom) 48 hours NHC (1 μM) treatment by immunostaining with the antibody against OROV Gc glycoprotein (red), cleaved-caspase-3 (green) and nuclei (DAPI, blue). Scale bar, 50 μm. (F) Gene set enrichment analysis (GSEA) in OROV-2024 infected organoids with NHC treatment for 96 hours compared to organoids with OROV infection alone. (G) Heatmap showing the expression of selected mitochondrial-related genes comparing uninfected controls, OROV-infected organoids at 96 hours post-inoculation and OROV-infected organoids with 96 hour NHC treatment. Data are representative of four biological replicates from one RNA-seq experiment (F, G). Mitochondrial-related genes were sourced from an online-published gene set (GSEA-MSigDB) and NCBI gene. The color scale represents row-wise Z-score of gene expression across samples. Statistical analysis for longitudinal data were performed using mixed-effects model (REML). Data represent mean ± SEM; $***p < 0.001$.

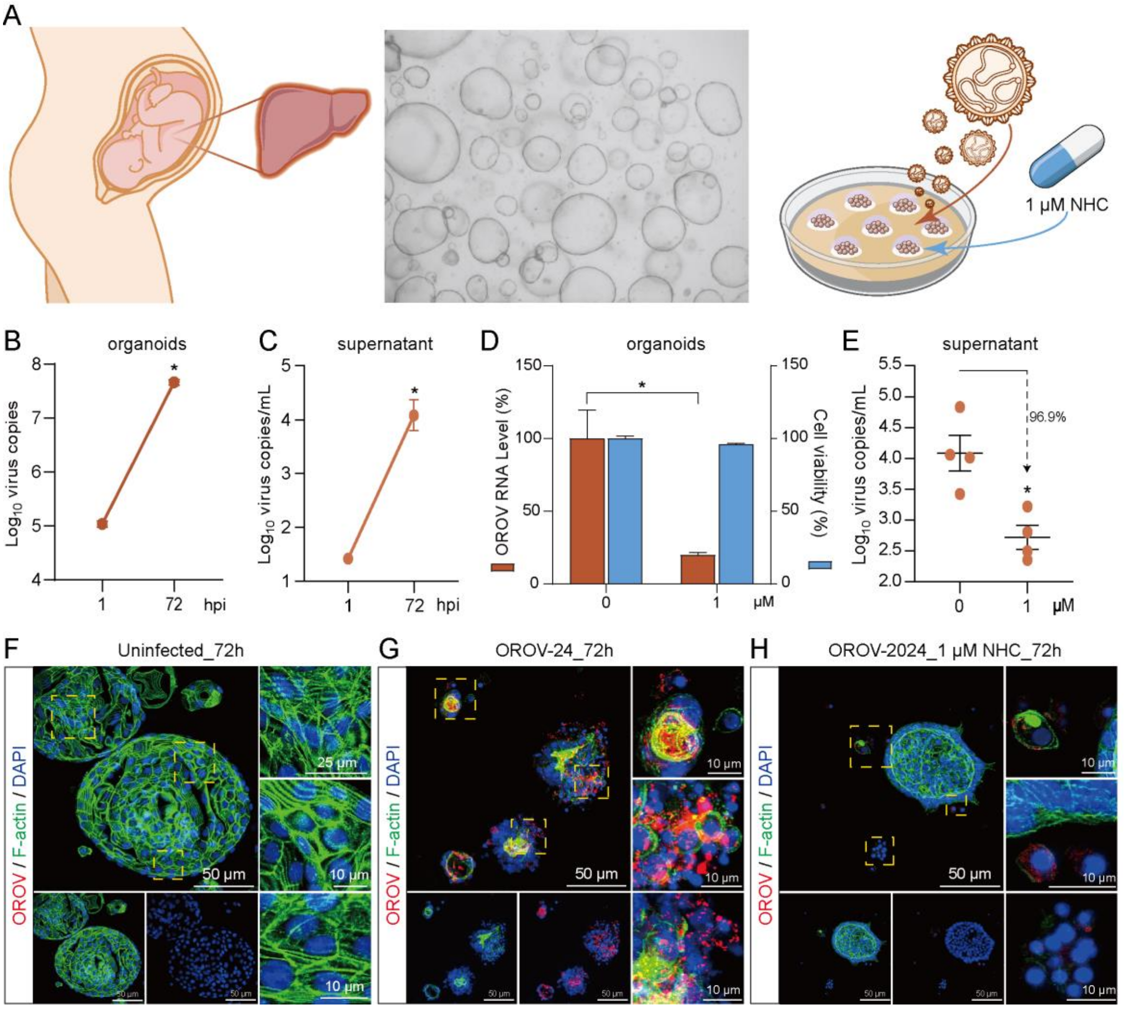

Figure S9. Human fetal liver-derived organoids support OROV infection but effectively inhibited by molnupiravir. Related to Figure 2 & 5.

(A) Schematic representation of fetal liver-derived organoids inoculated with OROV-2024 and 1 μM NHC treatment, and the bright field image of organoids. (B, C) Quantification of viral RNA copy number in organoids (B) and culture medium (C) after 1 hour and 72 hours inoculation with OROV-2024 (n = 4 biological replicates). (D) The inhibitory efficacy of 1 μM NHC on intracellular OROV RNA levels in organoids and corresponding cell viability after 72 hours treatment (n = 4 biological replicates). (E) The inhibitory efficacy of 1 μM NHC on OROV infectious titers in culture medium after 72 hours treatment (n = 4 biological replicates). (F-H) Representative images of uninfected organoids (F), OROV-2024 infected organoids without (G) or with (H) 72 hours NHC (1 μM) treatment by immunostaining with the antibody against OROV Gc glycoprotein (red), phalloidin for F-actin (green) and DAPI for nuclei (blue). Scale bar, 50, 25 and 10 μm. Data represent mean ± SEM; Statistical analysis by Mann-Whitney U test; $*p < 0.05$; ns, not significant.

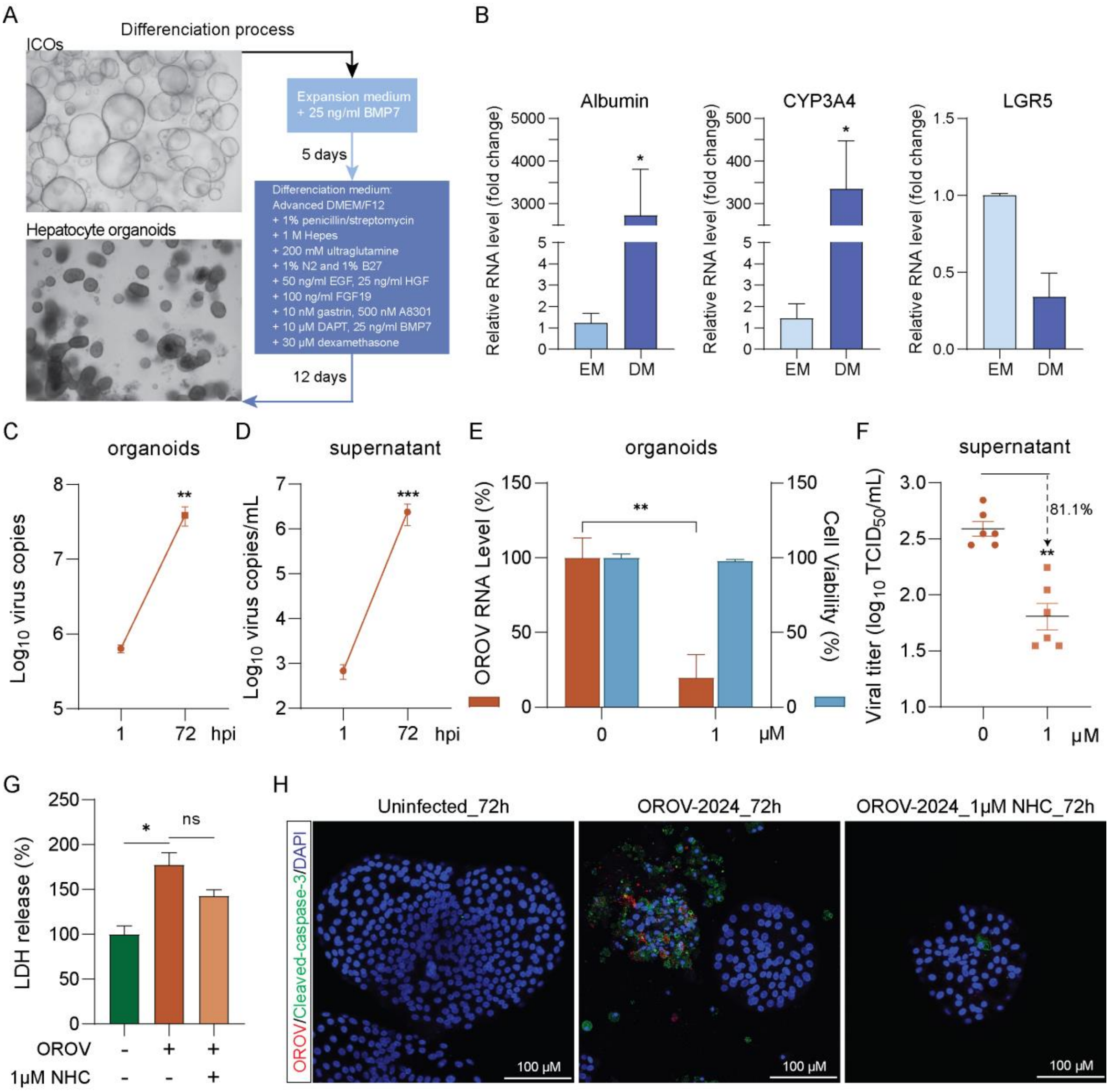

Figure S10. Hepatocyte-differentiated organoids are permissive to OROV infection and responsive to molnupiravir treatment. Related to Figure 2 & 5.

(A) Schematic of the differentiation protocol used to generate hepatocyte-differentiated organoids from intrahepatic cholangiocyte organoids (ICOs). (B) Quantification of hepatic marker genes (Albumin, CYP3A4) (n = 4 biological replicates from 2 donors) and progenitor marker (LGR5) (EM group: n = 4 biological replicates from 2 donors; DM group: n = 3 biological replicates from 2 donors) in organoids cultured in expansion medium (EM) or hepatocyte differentiation medium (DM). (C, D) Quantification of viral RNA copy number in organoids (C) (n = 6 biological replicates from 2 donors) and culture medium (D) (n = 10 biological replicates from 2 donors) after 1 hour and 72 hours inoculation with OROV-2024. (E) The inhibitory efficacy of 1 μM NHC on intracellular OROV RNA levels in organoids (n = 6 biological replicates from 2 donors) and corresponding cell viability (n = 8 biological replicates from 2 donors) after 72 hours treatment. (F) Infectious viral titers in culture supernatant from organoids treated with NHC at 48 and 96 hours (n = 6 biological replicates from 2 donors). (G) LDH release in culture supernatants of organoids at 72 hours post-inoculation, comparing uninfected, OROV-infected, and NHC-treated groups (1 μM) (n = 4 biological replicates from 2 donors). (H) Representative images

of uninfected organoids, OROV-2024 infected organoids without or with 72 hours NHC (1 μM) treatment by immunostaining with the antibody against OROV Gc glycoprotein (red), cleaved-caspase-3 (green) and DAPI for nuclei (blue). Scale bar, 100 μm. Data represent mean ± SEM; Statistical analysis by Mann-Whitney U test; $*p < 0.05$; $**p < 0.01$; $***p < 0.001$; ns, not significant.

Table S1. Characteristics and liver function tests for imported Oropouche cases in Europe. **Related to Figure 1.**

Case	Days from Symptoms Onset	OROV RT-PCR (Ct)	ALT (U/L)	AST (U/L)	GGT (U/L)	Age	Sex	Country	Reference
1	5	34.9	45	39	16	27	F	Italy	(Castilletti, Mori et al. 2024)
2	7	36.8	28	25	NA	55	F	Italy	(Castilletti, Mori et al. 2024)
3	18	35.4	22	23	NA	60	F	Italy	(Barbiero, Formica et al. 2025)
4	7	34.2	30	19	36	67	M	Italy	(Barbiero, Formica et al. 2025)
5	5	36.1	100	89	NA	42	M	Italy	Unpublished
6	6	32.4	149	111	72	41	M	Netherlands	(Igloi, Soochit et al. 2025)

Note: OROV RT-PCR was based on whole blood for the five cases in Italy, but was based on serum for the case in the Netherlands. All the cases were imported to Europe in 2024. Five of the six cases were reported in the previous studies, but the liver enzyme data were newly collected and were not reported previously. ALT: alanine aminotransferase; AST: aspartate aminotransferase; GGT: gamma-glutamyl transferase; NA: not available; F: female; M: male.

Table S2. Characteristics and liver function tests from a cohort of patients with Oropouche fever in Brazil. **Related to Figure 1.**

ID	Age	Sex	OROV RNA ct value	Alanine transaminase (ALT)	Aspartate aminotransferase (AST)	Gamma-glutamyl transferase (GGT)	Alkaline Phosphatase (ALP)	Direct Bilirubin (Conjugated)	Total Bilirubin	Indirect Bilirubin	Symptom onset date
1	53	FEMALE	25	48	55	9	41	0.01	0.1	0.09	2
2	41	MALE	24	36	38	38	65	0.03	0.24	0.21	3
3	49	FEMALE	21	96	98	47	73	0.05	0.19	0.14	2
4	55	MALE	25	55	55	13	59	0.03	0.14	0.11	0
5	31	MALE	20	27	16	27	41	0.01	0.07	0.06	1
6	27	FEMALE	22	23	23	18	69	0.03	0.09	0.06	2
7	76	MALE	18	41	38	18	48	0.02	0.13	0.11	4
8	30	FEMALE	20	89	72	64	71	0.01	0.1	0.09	2
9	40	FEMALE	26	61	52	21	67	0.03	0.13	0.1	1
10	22	FEMALE	21	35	34	18	47	0.04	0.17	0.13	2
11	60	MALE	19	21	21	23	74	0.02	0.53	0.51	3
12	54	FEMALE	18	12	14	12	30	0.02	0.11	0.09	1
13	24	MALE	20	47	41	22	53	0.03	0.13	0.1	2
14	74	FEMALE	22	71	65	106	65	0.08	0.44	0.36	1
15	73	MALE	24	31	30	43	75	0.03	0.15	0.12	3
16	65	FEMALE	25	11	15	20	38	0.04	0.19	0.15	4
17	23	MALE	25	59	74	45	54	0.03	0.11	0.08	0
18	21	FEMALE	23	31	32	24	58	0.01	0.11	0.1	0
19	66	MALE	21	73	56	236	95	0.04	0.23	0.19	1
20	31	MALE	23	21	22	16	57	0.04	0.18	0.14	1
21	31	FEMALE	23	34	21	40	43	0.01	0.06	0.05	1
22	26	MALE	27	31	33	23	59	0.03	0.14	0.11	1
23	52	MALE	23	71	64	67	112	0.06	0.47	0.41	1
24	56	MALE	24	21	23	113	109	0.05	0.2	0.15	1
25	75	FEMALE	23	96	130	73	91	0.04	0.14	0.1	2
26	60	FEMALE	25	73	73	288	100	0.03	0.14	0.11	1
27	48	MALE	25	9	25	20	36	0.01	0.09	0.08	2
28	54	FEMALE	27	68	71	31	45	0.04	0.14	0.1	3
29	55	MALE	25	NA	62	44	331	NA	NA	NA	1
30	47	MALE	18	85	70	58	225	NA	NA	NA	2

31	60	MALE	19	NA	58	39	NA	NA	NA	NA	3
32	73	MALE	24	NA	NA	530	601	NA	NA	NA	3
33	44	MALE	24	21	29	26	208	NA	NA	NA	1
34	59	MALE	27	NA	NA	52	NA	NA	NA	NA	3
35	56	MALE	24	NA	34	24	248	NA	NA	NA	1
36	74	FEMALE	22	191	215	101	362	NA	NA	NA	1
37	54	FEMALE	18	136	141	71	NA	NA	NA	NA	1
38	65	FEMALE	25	102	80	68	344	NA	NA	NA	4
39	21	FEMALE	23	57	63	10	123	NA	NA	NA	1
40	62	FEMALE	27	29	44	31	443	NA	NA	NA	1
41	31	FEMALE	23	143	106	35	346	NA	NA	NA	1
42	60	FEMALE	25	63	32	56	181	NA	NA	NA	1

Note: Clinical and laboratory characteristics of patients with Oropouche fever diagnosed during the 2024 outbreak in Brazil (July–August). Patients were matched by age, sex, and the time interval between symptom onset and sample collection. All cases were confirmed by real-time RT-qPCR targeting OROV, with a limit of detection of 2–20 copies, corresponding to Ct values ranging from 34.8 to 38.1.

Reference ranges for liver function tests were provided by Wiener Laboratories and are as follows: alanine aminotransferase (ALT), ≤41 U/L for males and ≤31 U/L for females; aspartate aminotransferase (AST), ≤38 U/L for males and ≤32 U/L for females; gamma-glutamyl transferase (GGT), 11–50 U/L for males and 7–32 U/L for females; alkaline phosphatase (ALP), ≤240 U/L for both sexes; total bilirubin, ≤1.2 mg/dL; direct bilirubin, ≤0.3 mg/dL; indirect bilirubin, ≤0.8 mg/dL. NA: not available.

Table S3. Summary of minor variant analysis in OROV genomes after serial passaging with or without NHC treatment. **Related to Figure 7.**

Segment	Position*	Nt change	AA change	Frequency in input	Frequency in CTR1	Frequency in CTR2	Frequency in CTR3	Frequency in NHC1	Frequency in NHC2	Frequency in NHC3
L	5498	T>C	-	39.5%	25.0%	27.2%	28.6%	47.5%	-	44.1%
L	5498	C>T	-	-	-	-	-	-	48.0%	-
M	556	G>A	V168I	41.9%	-	41.7%	33.7%	31.7%	47.4%	39.8%
M	556	G>A	I168V	-	40.8%	-	-	-	-	-
M	1684	G>T	A544T	42.7%	-	39.8%	42.8%	20.8%	26.3%	30.7%
M	1684	T > G	T544A	42.7%	40.2%	-	-	-	-	-
M	1892-1894	+GAC	Δ	-	27.9%	29.0%	27.0%	-	-	-
M	1892-1894	+CAA	Δ	-	-	-	-	-	21.6%	20.2%
M	2096	G>A	G680E	-	-	-	-	32.8%	28.9%	26.7%
M	3909	G>A	-	-	41.7%	42.4%	41.2%	-	-	-
M	3960	G>A	-	36.6%	35.9%	36.3%	34.3%	35.6%	40.0%	35.7%
S	66	C>T	-	-	29.0%	25.2%	22.8%	-	-	-
S	66	T>C	-	-	-	-	-	43.9%	40.2%	39.9%
S	375	T>A	-	-	25.1%	27.4%	23.4%	-	-	-
S	558	C>T	-	-	25.4%	27.5%	25.6%	-	-	-
S	567	C>T	-	-	25.2%	26.1%	24.4%	-	-	-

Note: Nucleotide and amino acid substitutions identified by Nanopore sequencing across L, M, and S segments of OROV. Only minor variants with a frequency over 20% are shown. Frequencies in the input virus, control passages (CTR1–CTR3), and NHC-treated passages (NHC1–NHC3) are presented. L, large segment encoding RNA-dependent RNA polymerase; M, medium segment encoding glycoproteins (Gn, Gc) and nonstructural protein; S, small segment encoding nucleocapsid protein N and the nonstructural protein; Nt, nucleotide; AA, amino acid; Δ, deletion

References

[1] GSEA-MSigDB. "https://www.gsea-msigdb.org/gsea/msigdb/human/geneset/MITOCHONDRIAL_PART.html."

[2] Barbiero, A., G. Formica, R. P. Mantovani, S. Accordini, F. Gobbi, M. Spinicci, M. G. Colao, S. Pollini, N. Ciccone, G. M. Rossolini, A. Bartoloni, L. Zammarchi and C. Castilletti (2025). "Persistent Oropouche virus viremia in two travellers returned to Italy from Cuba, July 2024." J Travel Med **32**(2).

[3] Castilletti, C., A. Mori, A. Matucci, N. Ronzoni, L. Van Duffel, G. Rossini, P. Sponga, M. L. D'Errico, P. Rodari, F. Cristini, R. Huits and F. G. Gobbi (2024). "Oropouche fever cases diagnosed in Italy in two epidemiologically non-related travellers from Cuba, late May to early June 2024." Euro Surveill **29**(26).

[4] Igloi, Z., W. Soochit, B. B. O. Munnink, A. A. Anas, K. J. von Eije, A. van der Linden, M. Mandigers, K. Wijnans, J. Voermans, F. D. Chandler, A. A. van der Eijk, C. GeurtsvanKessel, R. Molenkamp, R. S. Sikkema, B. Verstrepen and M. Koopmans (2025). "Oropouche Virus Genome in Semen and Other Body Fluids from Traveler." Emerg Infect Dis **31**(1): 205-206.
